# Nucleic acid drugs: recent progress and future perspectives

**DOI:** 10.1038/s41392-024-02035-4

**Published:** 2024-11-29

**Authors:** Xiaoyi Sun, Sarra Setrerrahmane, Chencheng Li, Jialiang Hu, Hanmei Xu

**Affiliations:** 1 Jiangsu Province Engineering Research Center of Synthetic Peptide Drug Discovery and Evaluation, China Pharmaceutical University, Nanjing, 210009 China; 2 NANJING ANJI BIOTECHNOLOGY CO. LTD, Nanjing, 210033 China

**Keywords:** Drug delivery, Drug delivery

## Abstract

High efficacy, selectivity and cellular targeting of therapeutic agents has been an active area of investigation for decades. Currently, most clinically approved therapeutics are small molecules or protein/antibody biologics. Targeted action of small molecule drugs remains a challenge in medicine. In addition, many diseases are considered ‘undruggable’ using standard biomacromolecules. Many of these challenges however, can be addressed using nucleic therapeutics. Nucleic acid drugs (NADs) are a new generation of gene-editing modalities characterized by their high efficiency and rapid development, which have become an active research topic in new drug development field. However, many factors, including their low stability, short half-life, high immunogenicity, tissue targeting, cellular uptake, and endosomal escape, hamper the delivery and clinical application of NADs. Scientists have used chemical modification techniques to improve the physicochemical properties of NADs. In contrast, modified NADs typically require carriers to enter target cells and reach specific intracellular locations. Multiple delivery approaches have been developed to effectively improve intracellular delivery and the in vivo bioavailability of NADs. Several NADs have entered the clinical trial recently, and some have been approved for therapeutic use in different fields. This review summarizes NADs development and evolution and introduces NADs classifications and general delivery strategies, highlighting their success in clinical applications. Additionally, this review discusses the limitations and potential future applications of NADs as gene therapy candidates.

## Introduction

The central dogma of genetics posits that nucleic acids carry human genetic information and play a crucial role in life processes, such as growth, development, and reproduction. Moreover, nucleic acids can be used to modify genetic information to treat various diseases.^1,2^ With the advancement of the life sciences, proteomics, and genomics methods, nucleic acid drugs (NADs) have been developed to translate and regulate nucleic acid functions.^3–5^ These drugs can achieve long-lasting efficacy through gene repression, replacement, and editing.^4,6^ Many studies have shown the feasibility of NADs in disease prevention and treatment.^7–9^ Thus, research and development on new classes of functional NADs are gradually emerging.

NADs are a class of gene therapy agents based on DNA, RNA, or synthetic oligonucleotide analogs. They have considerable potential for clinical applications, such as treating bacterial infections, tumors, and neuromuscular diseases.^10–12^ However, as negatively charged biological macromolecules, NADs have difficulty in crossing cellular membranes to enter cells. Additionally, they can be easily degraded by endogenous nucleases in plasma and tissues. Furthermore, few amounts of NADs that enter cells often become trapped by endosomes and subsequently degraded by lysosomes, considerably limiting their development and application.^13–15^ Currently, two main strategies exist to address the application challenges of NADs. One approach is to modify the nucleic acid structure to stabilize the properties of NADs and avoid recognition by the immune system. The other approach is to use delivery systems that facilitate their passage through cell membranes and ensure their localization to specific subcellular compartments. Consequently, the modification and transformation of NADs and the development of efficient, safe, and targeted delivery systems have become the primary focus of research and development on NADs.^16,17^

The accelerated development of the NADs field has relied on innovations and breakthroughs in foundational technologies, such as chemical synthesis, site-specific modifications, and delivery techniques. These advancements are crucial to ensure the safety, effectiveness, targeting, and applicability of NADs.^18,19^ Advancements in carrier technology and delivery systems have enhanced the biological activity of NADs, which improves their cellular targeting and uptake. Thus, the concentration and bioavailability of these drugs in target tissues are increased.^20^ Various delivery systems for NADs have been developed, including lipid nanoparticles (LNPs), cationic polymer complexes, and ligand-mediated nucleic acid molecular targeted delivery systems based on specific receptors, peptides, and other engineered carriers.^21–27^ However, these systems have several drawbacks, such as nonspecific distribution, inefficient cytoplasmic delivery, and suboptimal organelle targeting. Several studies have reported that more than one strategy is needed to address the delivery challenges. Thus, combining chemical structure modifications of nucleic acids with advanced drug delivery systems could achieve enhanced therapeutic effects.

This paper outlines the history of several significant molecular biology discoveries related to NADs, tracing key milestones from initial conceptualization to clinical applications. Then, we introduce the various NAD types and their modes of action, with an overview of both approved NADs and those currently in clinical trials. Then, we discuss the challenges associated with NADs development and explore strategies for overcoming the obstacles to in vivo delivery, including chemical modifications and delivery systems. Finally, we highlight the remaining challenges for NADs development, offering references for the design and clinical application of novel NADs.

## Concept and historical development of NADs

**Fig. 1:**
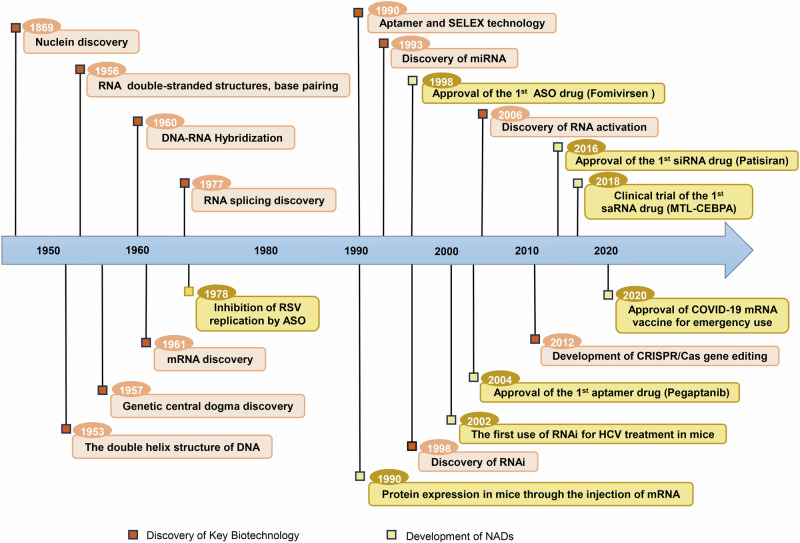
Historical timeline of essential discoveries in fundamental molecular biology theory and critical developments in NADs therapy. The orange boxes represent major biological discoveries in nucleic acids development, including the discovery of DNA and RNA, as well as researchers’ exploration of special biological phenomena such as RNA interference, nucleic acid hybridization, and gene editing. The yellow boxes show the breakthrough progress in the clinical application of NADs based on the aforementioned biological phenomena. These include successful clinical application cases of NADs, such as the first ASO drug Fomivirsen, the first siRNA drug Patisiran, the first aptamer drug Pegaptanib, the COVID-19 mRNA vaccines, as well as clinical trials for NADs in development, such as the saRNA drug MTL-CEBPA

NADs development is inseparable from the major discoveries in fundamental molecular biology and the continuous observations of life activities (Fig. 1). In 1869, Friedrich Miescher discovered a new molecule called “nuclein” from white blood cells, which marked the beginning of DNA discovery.^28^ However, owing to the lack of advanced technologies at the time, the critical role of nucleic acids was not fully understood. The subsequent revelation of DNA’s double helix structure and the formulation of the central dogma of genetics clarified that nucleic acids are crucial participants in transmitting genetic information.^1,2^ Since then, researchers have understood that genetic information is encoded within nucleic acids and translated into proteins via complex mechanisms, and it plays a vital role in all life processes, such as growth, development, and reproduction.

Additionally, there have been significant breakthroughs in NADs development including the discovery of RNA’s double-stranded structure and the phenomenon of nucleic acid hybridization.^29^ Early studies posited that single-stranded RNA could not form double-stranded structures. However, in 1956, Rich and Davies discovered that RNA could form double-stranded structures similar to those of DNA based on the principle of complementary base pairing. This result laid the foundation for developing RNA double-stranded drugs, including microRNAs (miRNAs) and small interfering RNAs (siRNAs).^30^ In 1960, Rich^31^ reported the phenomenon of DNA/RNA hybridization. In 1978, building on this discovery, Zamecnik and Stephenson^32^ used specific oligodeoxynucleotide chains that target the 35S RNA of the Rous sarcoma virus to inhibit virus replication, marking a prototype for the application of antisense oligonucleotide (ASO) drugs in disease treatment. As research on transcription and translation advanced, it was discovered that initial RNA transcripts typically require intron removal and the linkage of exons to form mature messenger RNA (mRNA), a process known as RNA splicing.^33,34^ RNA splicing is a crucial step in gene expression, and abnormalities in this process are the main cause of genetic variations and diseases.^35,36^ Dominski et al.^37^ found that ASOs targeting splicing sites can restore correct splicing of defective genes rather than only downregulating gene expression. This discovery provided a novel treatment strategy for diseases related to mis-splicing.

In 1998, Andrew Fire and Craig Mello reported that double-stranded RNA (dsRNA) had potent gene silencing effects in *Caenorhabditis elegans*,^38^ a discovery they termed RNA interference (RNAi). RNAi was quickly applied to inhibit the replication of the hepatitis C virus in mice,^39^ marking the earliest evidence of siRNA-mediated in vivo gene silencing. In 2006, they were awarded the Nobel Prize in Physiology and Medicine for their RNAi technology.^40^ Subsequently, researchers demonstrated that siRNA could be tailored to disrupt the expression of any pathogenic gene, propelling RNAi into the spotlight and fostering its active application in the treatment of various diseases. In 2010, the first human trial using RNAi technology was conducted to evaluate the therapeutic effect of siRNA for targeting the M2 subunit of ribonucleotide reductase in patients with melanoma.^41^ Since then, siRNA drugs have had issues with stability, immunogenicity, off-target effects, safety, and the delivery system. However, after advanced chemical modifications and the development of targeted delivery systems, the first siRNA drug approved by the Food and Drug Administration (FDA) in 2018 reignited interest in NADs.^42^

Notably, the discovery of RNA-dependent RNA polymerase and reverse transcriptase has been crucial for developing subsequent mRNA drugs.^43,44^ In 1984, Krieg and Melton used RNA polymerase extracted from viruses for in vitro transcription from engineered DNA templates, successfully achieving mRNA expression in cell-free systems.^45^ Thus, since the 1990s, in vitro-synthesized mRNA has been increasingly applied for protein replacement in preventive and therapeutic vaccines.^46–48^ In 2005, the discovery of pseudouridine (Ψ) modification addressed the immunogenicity issue of in vitro-synthesized mRNA,^49^ leading to the initiation of the first human trial of an mRNA vaccine against melanoma in 2008. In 2020, during the COVID-19 pandemic, the FDA authorized the emergency use of mRNA vaccines, providing effective measures for preventing and controlling the virus.^50,51^ This resulted in widespread public attention to NADs.^52^ In 2023, the Nobel Prize in Physiology and Medicine was awarded to Katalin Kariko and Drew Weissman for pioneering nucleoside base modification technology to decrease mRNA immunogenicity, further highlighting the critical role of chemical modification technologies in developing mRNA vaccines. This opened new clinical applications for NADs in treating human diseases.^53,54^

Simultaneously, discovering other types of nucleic acids and biological phenomena has further expanded the scope of NADs. Specifically, the emergence of gene-editing technology has provided a foundation for developing new therapies for genetic mutation diseases.^55–57^ Recently, the first gene therapy based on clustered regularly interspaced short palindromic repeats (CRISPR)/Cas9 technology has been approved for marketing,^58^ resulting in revolutionary changes in NADs development.

## Classification and therapeutic mechanisms of NADs

NADs can be broadly divided into three categories based on their mechanisms of action. The first category includes NADs that target nucleic acids to regulate protein expression by promoting or inhibiting translation. This category primarily consists of ASOs, siRNAs, miRNAs, small activating RNAs (saRNAs), and the CRISPR/Cas system, which enables precise gene editing of genomic DNA.

The second category includes NADs that target proteins, with aptamers as the main examples. Unlike the first category, aptamers can directly and specifically bind to target proteins, functioning similarly to antibodies by providing a targeting mechanism.

**Fig. 2:**
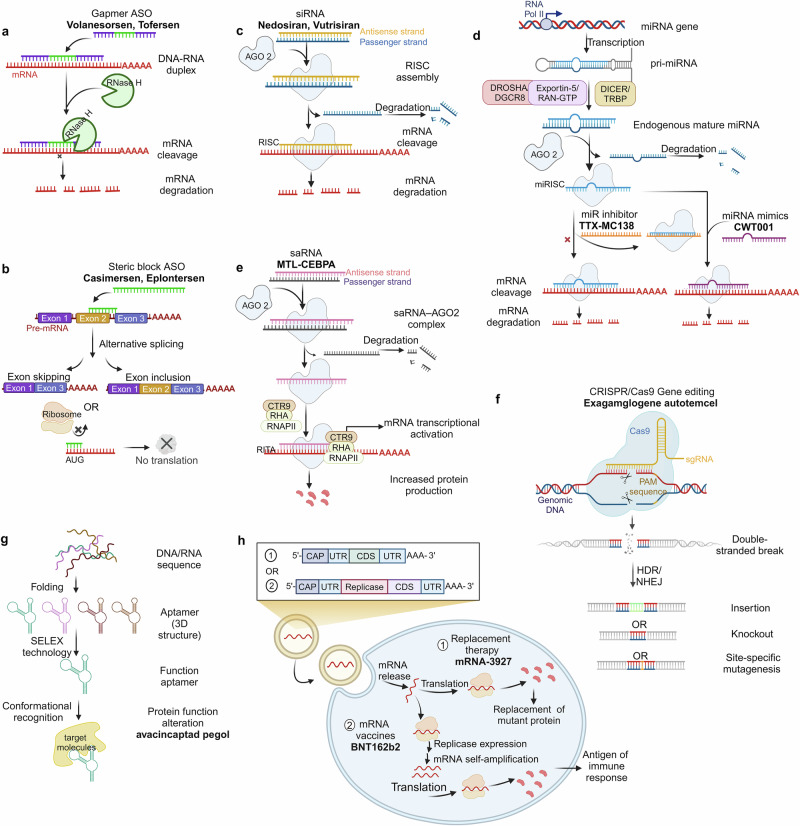
Classification and therapeutic mechanisms of NADs. **a** Gapmer ASO (consisting of a DNA-based internal gap with RNA-like flanking regions) binds to target mRNA with high affinity to form an RNA-DNA duplex and participates in RNase H-mediated mRNA degradation. **b** Steric block ASO regulates functional target gene expression through exon skipping or exon inclusion or interrupts translation initiation by targeting and masking the AUG start codon of the target mRNA. **c** siRNAs form RISC with AGO2. While the passenger strand is discarded, the antisense strand binds to the target mRNA, downregulating the translation level of the target mRNA. **d** pri-miRNAs produced by miRNA gene transcription in the non-coding region are processed to form mature miRNAs with the help of a series of complexes (Drosha/DGCR8, Exportin-5/RAN-GTP, and Dicer/TRBP). miRNAs combine with the AGO2 to form miRISC. The activity of miRNAs can be inhibited by miRNA inhibitors that either form a complex with the mature miRNA loaded in the miRISC complex or by masking a target site via interactions with the specific transcript being targeted. **e** saRNAs recruit the RITA complex (including AGO2, CTR9, RHA, and RNAP II) to stimulate the initiation and extension of transcription. **f** CRISPR-mediated gene editing mainly uses Cas9 and sgRNA to introduce DSBs at specific positions in the genome effectively. DSBs are generally repaired by HDR or NHEJ, achieving insertion, knockout, and site-specific mutagenesis. **g** Aptamers screened by SELEX technology can recognize specific proteins by forming 3D structures. **h** Exogenous mRNAs introduced into cells undergo translation to proteins and facilitate protein function through protein replacement therapy and mRNA vaccines

The third category includes NADs that express proteins, such as in vitro-transcribed mRNA, which can produce specific proteins in vivo to exert biological activity. This section briefly introduces the mechanisms of action of these different NAD types and highlights drugs successfully applied in clinical settings (Fig. 2).

### NADs that target nucleic acids

#### ASOs

ASOs are artificially synthesized single-stranded oligonucleotide chains that regulate and target RNA’s function via specific binding according to the Watson-Crick base-pairing principle.^59^ The mechanisms of action of clinically used ASOs mainly include ribonuclease H (RNase H)-mediated degradation (Fig. 2a) and steric blockage mechanisms (Fig. 2b).^60–62^ RNase H-dependent ASOs, such as gapmers, bind to complementary mRNA, recruit RNase H to cleave mRNA, and thus block translation of the target gene. This results in the inhibition or reduction of the synthesis of the target protein. Representative drugs based on this mechanism include fomivirsen (Vitravene®), mipomersen (Kynamro®, Delisted), inotersen (Tegsedi®), volanesorsen (Waylivra®), and tofersen (Qalsody®).^63–67^ Because RNase H is active in the nucleus and cytoplasm, ASOs can also target other transcripts, such as long non-coding RNAs.^68,69^ Indeed, the pathogenesis of many diseases without clear protein targets is related to non-coding RNA, which can be used to predict the advantages of ASO in treating diseases.^70,71^

Additionally, ASOs can form a double-stranded structure complementary to miRNA, leading to its degradation and causing gene upregulation.^72–74^ Additionally, ASOs regulate transcription via steric hindrance, influencing specific splicing of pre-mRNA and selectively altering the expression of specific proteins.^75,76^ ASOs that employ this mechanism are splicing-switching oligonucleotides, which modulate functional target gene expression by skipping or including exons.^77–79^ For example, Golodirsen, an ASO drug targeting the human Duchenne muscular dystrophy (DMD) gene, was introduced in the USA in 2019 by Sarepta Therapeutics to treat DMD patients with confirmed mutations amenable to exon 53 skipping.^80^ Additionally, several studies have demonstrated that ASOs can disrupt translation initiation by targeting and masking the AUG start codon of the target mRNA.^81^ These discoveries have enabled ASOs to be applied in disease treatment via more diverse mechanisms.

#### siRNA

siRNA is a dsRNA molecule that is typically 19–23 base pairs long and is found naturally in various organisms or artificially synthesized.^82,83^ As a classical effector of RNAi, siRNA induces gene silencing by blocking mRNA translation.^84,85^ Unlike ASOs, siRNA-mediated gene silencing occurs via the RNA-induced silencing complex (RISC), not RNase H. Once mature siRNA enters the cell, it forms a complex with the Argonaute-2 (AGO2) protein (Fig. 2c).^86,87^ As the passenger strand of siRNA is degraded, the antisense strand binds to the target mRNA. It guides RISC to cleave the target sequence, thus achieving therapeutic effects by downregulating the translation of specific proteins.^88–90^ Based on this mechanism, researchers have designed siRNAs that target specific pathological genes to achieve specific gene silencing using RNAi.^91,92^ However, challenges, such as stability, specificity, and delivery obstacles, hindered progress in the early stages of development.^93–96^ Advancements in carrier technology and nucleic acid modification techniques have led to significant progress in overcoming these challenges, as exemplified by the first siRNA drug, patisiran (Onpattro®), which has been approved for treating hereditary transthyretin-mediated amyloidosis by degrading mRNA encoding transthyretin (TTR).^97^ Thus far, six siRNA drugs have received international approval. With the ongoing development of novel chemical modifications and targeted delivery systems, more siRNA drugs are anticipated to enter the market soon.

#### miRNA

Endogenous non-coding RNAs that have been discovered in eukaryotic organisms can act as gene regulators. Since the 1993 discovery of the first miRNA, lin-4, in the nematode C. elegans,^98^ miRNAs have been shown to participate in various biological functions and pathological mechanisms, including cell proliferation, differentiation, migration, disease occurrence, and progression.^99^ There has been extensive research on the regulatory mechanisms of miRNAs. With the assistance of complexes, such as Drosha/DGCR, Exportin-5/RAN-GTP, and Dicer/TRBP, primary miRNAs (pri-miRNAs) transcribed from the non-coding region of miRNA genes are processed to form mature miRNAs (Fig. 2d).^100–105^ miRNAs combine with Argonaute proteins to form the miRNA-induced silencing complex (miRISC), which can silence the target transcript via base pair complementation.^106,107^ Unlike siRNA-mediated gene silencing, miRNAs can simultaneously recognize and regulate the expression of multiple target mRNAs due to their low complementarity.^108,109^ The antisense strand of miRNA can bind to the target mRNA via complete and incomplete complementarity, leading to the cleavage of target mRNA and the inhibition of target gene expression.^110,111^ Contrary to the classical gene silencing mechanism, research has shown that miRNAs can interact with 3’-UTR to upregulate gene expression, indicating the complexity and diversity of miRNA regulatory mechanisms.^112–114^

As studies on the roles of miRNAs in diseases continue to be reported, there is anticipation for the potential use of miRNAs in different pathological processes. However, thus far, no miRNA drugs have been approved for the market.^115,116^ miRNA drugs’ ongoing development primarily includes two categories: miRNA mimics and miRNA inhibitors.^117–121^ miRNA mimics are synthetic dsRNA molecules that mimic the function of endogenous miRNAs. Similarly, miRNA inhibitors are single-stranded RNAs that are complementary to endogenous miRNAs, which can reduce the effect of gene silencing by specifically inhibiting miRNA.^122,123^ The miRNA drug MRG-201 (Remlarsen), developed by Viridian Therapeutics (formerly known as miRagen Therapeutics), simulates microRNA-29b to reverse regulate fibrosis, thus inhibiting fibrous proliferation in skin wounds. Clinical trials have been conducted to evaluate the efficacy and safety of MRG-201 in treating fibrotic diseases (NCT03601052).^124^ In addition to MRG-201, Viridian Therapeutics has several other miRNA-based drugs, including MRG-106 (Cobomarsen), which targets miR-155 to inhibit tumor development, and MRG-110, which targets microRNA-92 to promote angiogenesis.^125,126^ TTX-MC138 (NCT06260774 and NCT05908773), designed by TransCode Therapeutics, targets microRNA-10b in the treatment of pancreatic cancer.^127^ Despite the relatively slow progress in miRNA-related drug development compared with other NADs, their superior performance in treating tumors, heart failure, and diabetes indicates their promising prospects in clinical treatment.

#### saRNA

In addition to the classical gene silencing mechanism, the discovery of RNA activation has provided a new perspective on gene regulation. RNA activation is a gene regulation phenomenon mediated by a small dsRNA called saRNA, which targets gene promoter sequences to enhance the transcription of the target gene.^128,129^ After entering the cell via endocytosis, saRNA binds to AGO2. The sense strand is released and degraded, as AGO2 and the antisense strand are transported into the cell nucleus, where they bind to the promoter region (Fig. 2e). Subsequently, AGO2 recruits RNA polymerase-associated protein CTR9 homolog (part of the polymerase-associated factor 1 complex) and RNA helicase II (RHA) to form the RNA-induced transcriptional activation (RITA) complex. This complex interacts with RNA polymerase II (RNAP II) and stimulates the initiation and elongation of transcription.^130,131^ saRNAs are conserved in mammals and participate in the activation of various genes, such as vascular endothelial growth factor A (VEGFA), E-cadherin, progesterone receptor, and Kruppel-like factor 4.^128,132–135^ This conservation allows for the establishment of animal models and preclinical studies for saRNA-based therapies. Targeted activation therapy using saRNA has been validated in various disease animal models.^129,136–140^ However, clinical translation of saRNAs remains challenging due to the absence of an immune system in xenograft models and the complexity of drug action in the human body.^141^

The leading saRNA drug, MTL-CEBPA, which was developed by MiNA Therapeutics, targets the CCAAT/enhancer-binding protein alpha (CEBPA). Phase I trial results (NCT05097911) have demonstrated that MTL-CEBPA has a good safety profile for the treatment of hepatocellular carcinoma and may enhance the therapeutic effects of tyrosine kinase inhibitors by modulating immune suppression.^142–144^ In addition to MTL-CEBPA, MiNA Therapeutics has several saRNA candidates in preclinical development for undisclosed tumor types, metabolic diseases, and genetic disorders. RAG-01 is another saRNA candidate drug designed to treat bladder cancer by activating the expression of the human cyclin-dependent kinase inhibitor 1 A.^145^ Ractigen Therapeutics has submitted an Investigational New Drug application for RAG-01, and it is expected to become the second saRNA drug to enter clinical trials internationally.

### CRISPR/Cas9 system

Gene editing is a genetic engineering technology that precisely modifies target genes via insertion, deletion, and site-specific mutation. The CRISPR/Cas9 system has gained widespread attention among the various genome-editing methods. As a prokaryotic adaptive immune system, CRISPR/Cas modules establish bacterial defense against exogenous nucleic acids. They have been widely used in gene editing owing to their high efficiency and accuracy.^146–149^ The CRISPR-mediated gene-editing system primarily involves Cas9 and single guide RNA (sgRNA) to introduce DNA double-strand breaks (DSBs) at specific positions in the genome. These DSBs are usually repaired by homologous directed repair (HDR) or nonhomologous end joining (NHEJ), achieving mutation or foreign gene insertion (Fig. 2f).^150,151^ CRISPR/Cas9 technology has been effective in various fields, including hematologic disorders, malignant tumors, and genetic diseases.^151–153^ However, this technology has several challenges, such as low delivery and HDR efficiency, off-target effects, and toxic side effects.^154–157^ Currently, the CRISPR/Cas9 system can enter cells for gene editing in three forms: the Cas9: sgRNA ribonucleoprotein complex, mRNA for Cas9 translation alongside a separate sgRNA, and a DNA plasmid that encodes both the Cas9 protein and sgRNA.^158–160^ Each method has its pros and cons. The plasmid system is relatively stable but has low cutting and editing efficiency, and continuous expression of Cas9 may increase off-target effects.^161^ mRNA and sgRNA are susceptible to degradation by nucleases. The Cas9/sgRNA ribonucleoprotein complex, while the most responsive mode of action, has a low off-target rate and low toxicity, but the large size of the complex complicates delivery.^160^ The delivery of the CRISPR/Cas9 system is a crucial issue limiting its clinical application. Like other NADs, researchers have focused on nanocarriers based on liposomes, chitosan, and other materials to achieve efficient delivery, laying a solid foundation for the clinical application of the CRISPR/Cas9 system.^162–164^ Exa-cel (Casgevy®) was approved in November 2023 as the first gene-editing therapeutic drug based on the CRISPR/Cas9 system for treating sickle cell disease (SCD) and transfusion-dependent β-thalassemia (TDT). Exa-cel stimulates artificial blood stem cells and progenitor cells in vitro to differentiate into red blood cells that produce high fetal hemoglobin levels.^165,166^ The launch of Exa-cel has filled the gap in gene-editing drugs and provided considerable motivation for developing other gene-editing therapies.

### NADs that target proteins

Aptamers are single-stranded oligonucleotide molecules that have specific recognition functions obtained via iterative screening from large libraries of random oligonucleotides using the systematic evolution of ligands by exponential enrichment (SELEX) technology.^167–169^ Unlike other nucleic acid-based drugs, aptamers specifically recognize and bind target molecules, such as peptides, proteins, viruses, bacteria, and cells, relying on their unique three-dimensional conformation. This is similar to the conformational recognition that mediates antibody–antigen interactions and complex formation (Fig. 2g).^170–173^ However, compared with antibodies, aptamers have several advantages, including high thermal and physiological stability, low immunogenicity, and a wider range of target specificity. Since their first report in 1990, SELEX technology has been continuously improved,^174,175^ diversifying the selection and development of high-affinity aptamers.^176,177^ Aptamers have shown promising applications in treating cancer and ophthalmic and cardiovascular diseases (CVDs).^178–180^

In 2004, the FDA approved the first aptamer drug, pegaptanib (Macugen®), to treat choroidal neovascularization caused by neovascular age-related macular degeneration (AMD).^181^ However, Pegaptanib was withdrawn from the market owing to its poor efficacy and competition from anti-VEGF antibody drugs, such as Lucentis.^64^ Despite this, research on aptamers continues. In August 2023, avacincaptad pegol was approved for treating geographic atrophy (GA) secondary to dry AMD. Complex cascade overactivity is likely instrumental in AMD pathology. A crucial complement component, C5, has become a primary therapeutic target for many inflammatory diseases, including AMD.^182^ As a C5 inhibitor, avacincaptad pegol has been shown to slow GA progression by targeting the source of retinal cell death. Additionally, AS1411 is a candidate drug that targets nucleolin, which has been confirmed to be effective for treating renal cell carcinoma,^183^ glioma,^184^ and acute myeloid leukemia.^185^ In addition, aptamers are widely used in drug delivery, clinical diagnostics, and biosensing.^186–188^ This paper primarily focuses on their application as therapeutic drugs. Thus, we will not describe their other uses in detail further.

### NADs that express proteins

mRNA, a single-stranded polynucleotide that carries genetic information, is essential for expressing encoded proteins within cells, thus exerting corresponding biological functions via these proteins. Consequently, the concept of using mRNA as NADs has been proposed. In 1990, Wolff et al.^46^ injected in vitro-synthesized mRNA into mouse skeletal muscle and successfully induced the expression of specific proteins. They demonstrated the feasibility of using in vitro-synthesized mRNA as an information carrier to guide somatic protein synthesis.

However, the development of mRNA-based drugs has resulted in several challenges. Unmodified mRNA can induce Toll-like receptor-mediated immune responses, leading to blocked protein synthesis. Kariko and Weissman discovered that nucleobase modifications could protect mRNA from triggering inflammatory responses.^49,54^ In addition, the large size of mRNA and its susceptibility to degradation by nucleases result in low cellular uptake, further limiting its application. To overcome these obstacles, researchers have developed various delivery systems, including lipids, peptides, and polymers, for mRNA delivery both in vitro and in vivo.^189–191^

In contrast to the previously mentioned NADs that exert therapeutic effects by directly binding mRNA or proteins, two strategies for the use of mRNA drugs have been attempted (Fig. 2h). One strategy is protein replacement therapy, which involves introducing exogenous mRNAs into cells to express functional proteins or supplement deficient ones.^192,193^ For example, one research group used LNPs to deliver mRNA encoding erythropoietin into mouse fetuses, thus increasing erythropoietin protein levels in the mouse bloodstream.^192^ Additionally, mRNA therapy has been applied to treat patients with deficiencies in essential enzyme genes, such as argininosuccinate lyase, ornithine transcarbamylase, and methylmalonyl-CoA mutase (MUT), therefore restoring enzyme levels and mitigating deficiencies.^194–196^ Moderna, a company dedicated to mRNA therapy, has several mRNA drugs based on enzyme replacement therapy in clinical trials. For instance, mRNA-3927, which encodes the alpha and beta subunits of the propionyl-CoA carboxylase enzyme, is designed to treat propionic acidemia. A Phase I/II trial (NCT05130437) for this indication has been initiated in pediatric patients to evaluate the long-term safety of mRNA-3927.^197^ Additionally, mRNA-3704 and mRNA-3705 encode MUT, which is designed to treat methylmalonic acidemia and is currently under investigation.

The second approach involves mRNA vaccines, which activate the body’s immune response to combat infectious diseases and tumors by directly translating mRNA-containing antigen proteins. Compared with traditional inactivated vaccines, mRNA vaccines have advantages such as cell-free production, high production efficiency, and low cost. Thus, they are quite promising for addressing sudden epidemic infectious diseases. During the COVID-19 pandemic, mRNA prophylactic vaccines were crucial among all candidate vaccine types. The FDA authorized the emergency use of BNT162b2, which was developed by Pfizer-BioNTech, and mRNA-1273, developed by Moderna.^198,199^ Moreover, the development of mRNA vaccine technology has been propelled by large-scale clinical trials of mRNA vaccines. For example, ARCov, which was jointly developed by Abogen Biosciences, the Academy of Military Medical Sciences, and Walvax Biotechnology, has addressed the issue of poor thermal stability of mRNA vaccines.^200,201^ Additionally, preventive vaccines against influenza viruses,^202,203^ respiratory syncytial virus,^204^ rabies virus,^205^ and other viruses, as well as cancer-targeted therapeutic vaccines,^206–208^ are being continuously researched and developed, which further reflect the application prospects of mRNA vaccines.

In summary, in recent decades, NADs development has undergone significant progress and achieved considerable results. More than 20 products based on ASOs, aptamers, siRNA, and mRNA have been approved for marketing to treat various rare genetic disorders (Table 1). Several companies worldwide have been active in this field, attracting substantial investment, and the market development space is expected to expand further.

**Table 1:** NADs approved for clinical application

Classification	Drug (brand name)	Company	Indication	Dose (route)	Target (organ)	Modification & delivery	Approval year	Ref(s)
ASO	Fomivirsen (Vitravene)	Ionis	CMV retinitis	330 μg per eye once every 4 weeks (ITV)	CMV UL123 (eye)	PS	1998 (delisted)	^234,578^
	Mipomersen (Kynamro)	Ionis	HoFH	200 mg once weekly (SC)	ApoB-100 (liver)	2’-MOE	2013 (delisted)	^579^
	Eteplirsen (Exondys 51)	Sarepta Therapeutics	DMD	30 mg kg^−1^ once weekly (IV)	Exon 51 of DMD (muscle)	PMO	2016	^497,498^
	Nusinersen (Spinraza)	Ionis & Biogen	SMA	12 mg once every 4 months (IT)	Exon 7 of SMN2 (CNS)	2’-MOE	2016	^580,581^
	Inotersen (Tegsedi)	Ionis	hATTR	300 mg once weekly (SC)	TTR (liver)	2’-MOE	2018	^490,491^
	Volanesorsen (Waylivra)	Ionis	FCS	300 mg once weekly (SC)	ApoC-III (liver)	2’-MOE	2019	^66,582^
	Golodirsen (Vyondy 53)	Sarepta Therapeutics	DMD	30 mg kg^−1^ once weekly (IV)	Exon 53 of DMD (muscle)	PMO	2019	^80^
	Viltolarsen (Viltepso)	Nippon Shinyaku	DMD	80 mg kg^−1^ once weekly (IV)	Exon 53 of DMD (muscle)	PMO	2020	^263,583^
	Casimersen (Amondys 45)	Sarepta Therapeutics	DMD	30 mg kg^−1^ once weekly (IV)	Exon 45 of DMD (muscle)	PMO	2021	^264,496^
	Tofersen (Qalsody)	Biogen	ALS	100 mg once every 28 days (IT)	SOD1(CNS)	PS/ 2’-MOE	2023	^67,505^
	Eplontersen (Wainua)	Ionis & AstraZeneca	ATTRv-PN	45 mg once monthly (SC)	TTR (liver)	LICA	2023	^492^
siRNA	Patisiran (Onpattro)	Alnylam	hATTR	0.3 mg kg^−1^ once every 3 weeks, max:30 mg (IV)	TTR (liver)	2’-OME & LNP	2018	^97^
	Givosiran (Givlaari)	Alnylam	AHP	2.5 mg kg^−1^ once monthly (SC)	ALAS1(liver)	PS/ 2’-OME & GalNAc	2019	^584^
	Lumasiran (Oxlumo)	Alnylam	PH1	3 or 6 mg kg^−1^ once every 3 months (SC)	HAO1(liver)	PS/ 2’-OME & GalNAc	2020	^510^
	Inclisiran (Leqvio)	Alnylam & Novaetis	primary hypercholesterolemia	284 mg at 0 and 3 months, and then once every 6 months (SC)	PCSK9(liver)	PS/ 2’-OME & GalNAc	2020	^539^
	Vutrisiran (Amvuttra)	Alnylam	hATTR-PN	25 mg once every 3 months (SC)	TTR (liver)	PS/ 2’-OME & GalNAc	2022	^493^
	Nedosiran (Rivfloza)	Novo Nordisk & Dicerna	PH1	128 or 160 mg once monthly (SC)	LDHA (liver)	PS/ 2’-OME & GalXC^TM^	2023	^330^
CRISPR/Cas	Exagamglogene autotemcel (Casgevy)	Vertex & CRISPR Therapeutics	TDT & SCD	Once (IV)	BCL11A	/	2023	^165,166^
Aptamers	Pegaptanib (Macugen)	EyeTech Pharmaceuticals & Pfizer	AMD	0.3 mg once every 6 weeks (ITV)	VEGF-165 (eye)	pegylated	2004 (delisted)	^181^
	avacincaptad pegol (Izervay)	Iveric Bio	GA	2 mg once monthly (ITV)	complement C5 (eye)	pegylated	2023	^585^
mRNA	BNT162b2 (Comirnaty)	Pfizer & BioNTech	COVID-19	IM	SARS-CoV-2(liver)	LNP	2020	^199^
	Elasomeran (Spikevax)	Modern	COVID-19	IM	SARS-CoV-2(liver)	LNP	2020	^586^
	mRNA-1345 (mRESVIA)	Modern	Respiratory syncytial virus infection	IM	Respiratory syncytial virus (lung)	LNP	2024	^587^

## Current challenges in NADs development

**Fig. 3:**
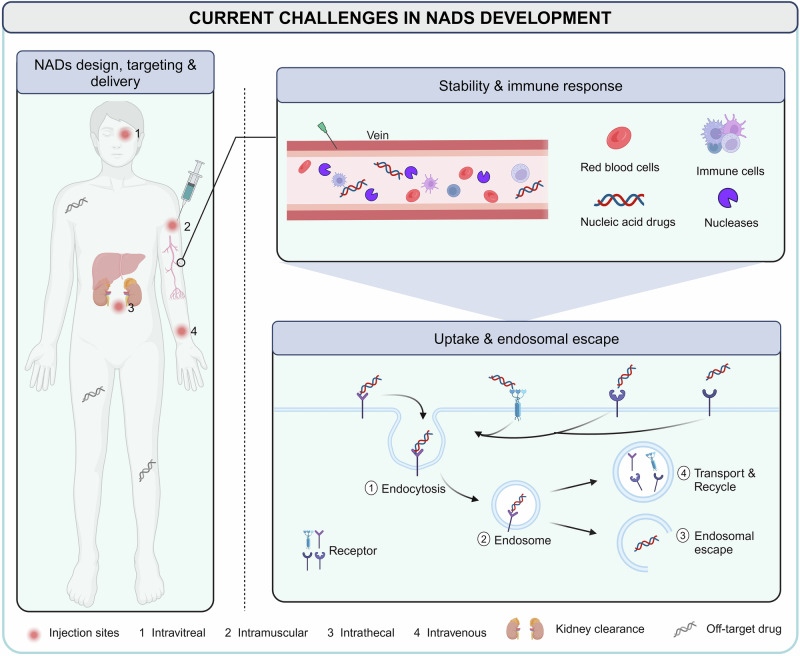
Current challenges in NADs delivery. NADs are administered in many ways, such as intravitreal, intramuscular, intrathecal, and intravenous injection. For systemic delivery, NADs must first overcome renal clearance, nuclease degradation, immune system recognition, and drug off-target until reaching target tissues and organs. Subsequently, NADs successfully reach the target cells, enter the cell via endocytosis, enter the endosomes, and escape successfully to achieve the desired therapeutic effect. It is difficult for negatively charged NADs to cross the phospholipid bilayer on the surface of the cell membrane, which usually requires the help of carriers to recognize receptors or chemical modification of NADs to change properties

Unlike traditional small molecules and antibody drugs that exert their pharmacological effects on proteins, most NADs directly regulate gene expression, offering a broader range of targets. This is particularly valuable for addressing genes with defective proteins that are difficult to be targeted with conventional drugs, showing considerable potential in treating rare, chronic, infectious diseases and other metabolic disorders. Despite these advantages, researchers must accurately identify the genetic information related to the disease and choose the appropriate type of NADs based on the mechanism of action.^209,210^ For targeted NADs, including ASO and siRNA, when the relevant genetic information of the disease is determined, lead compounds can be designed for the gene sequence to avoid off-target effects during development.^211^ The efficacy of aptamer drugs is related to their sequence and conformation. SELEX technology has been applied to better screen specific sequences with high affinity for a target from a randomly generated single-stranded nucleic acid sequence library.^212^ Understanding the relationship between the mRNA sequence, structure, function, and stability for mRNA development is important to ensure the maximum functional protein output of delivered mRNA molecules.^213^ In recent years, the NADs sequence design process has been accelerated by the development of advanced bioinformatics tools, considerably reducing the time and costs.^209,210,214,215^ Nonetheless, the major obstacle in NADs development involves how to reach target cells to fully achieve therapeutic benefits (Fig. 3).^13,216,217^ The most relevant challenges can be summarized as follows.

### Pharmacokinetics, stability, and degradation

Naked nucleic acids have poor in vivo stability and can degrade in the bloodstream. They undergo either enzymatic (nucleases and RNAse) or chemical (oxidation and hydrolysis) degradation in the blood and tissue fluids or are filtered and cleared by the kidneys.^218^ Researchers have implemented several chemical modifications to enhance NADs stability, but almost all these methods can affect the efficacy and safety.^219^

### Immunogenicity

Exogenous nucleic acids can be recognized as exogenous signals by pattern recognition receptors in the immune system, triggering immune responses that compromise the structural integrity and stability of the nucleic acids. Careful design and modification of NADs can mitigate immunogenicity, but this requires extensive testing.^220,221^

### Targeting problems

Therapeutic nucleic acids often lack sufficient targeting ability in vivo. This insufficient targeting leads to low NADs concentrations at the disease site and unintended gene silencing or activation, potentially causing safety issues by requiring higher doses. Designing particular nucleic acids and conducting thorough off-target screening are critical for the efficient clinical application of NADs.

### Uptake efficiency and endosomal escape

Efficient uptake of NADs into cells is difficult owing to their size, charge, and hydrophilicity. Negatively charged nucleic acids do not easily cross the negatively charged lipid bilayer of the cell membrane. Additionally, endosomal escape is a significant barrier as nucleic acids often become trapped in endosomes and degraded. After entering cells, NADs are often captured by endosomes. Only those NADs that successfully escape the endosomes can exert their therapeutic effects. Inadequate endosomal escape can result in reduced efficacy and increased off-target toxicity.^222^

In addition to the previous issues, NADs face manufacturing and scalability challenges. Producing NADs at scale with consistent quality requires advanced manufacturing technologies and stringent quality control measures. This results in a relatively high cost, affecting accessibility and affordability for patients.

## Strategies to improve NADs performance

Because of these challenges, accurately delivering drugs to target tissues and improving patient quality of life have become the core objectives for NADs development and research. Recently, significant advancements have been made in chemical modification technologies and delivery vehicles for NADs, considerably enhancing delivery efficiency.^16^ Thus far, dozens of NADs have benefited from successful chemical modification and carrier delivery and have earned FDA approval. For example, the success of COVID-19 vaccines is mainly due to advancements in base modification and LNP delivery systems. Therefore, the focus of NADs research and development has shifted toward improving nucleic acid modifications and developing efficient, safe, and targeted delivery systems.

### Chemical modification

Various chemical modification methods have been introduced with the continuous progress of chemical synthesis and modification technologies to provide more precise treatments.^223,224^ NADs can be precisely modified to improve their efficacy and stability while decreasing toxicity and immunogenicity.^223,224^ The most widespread modification methods include backbone, ribose, and nucleobase modifications.^225–228^

#### Backbone modification

The modification strategies used in first-generation NADs have focused on modification of the phosphate backbone, mainly using other types of groups to replace the non-bridging oxygen atoms in the phosphate backbone, such as phosphorothioate (PS), methyl phosphate, and boranophosphate.^229^ The most often used backbone modification method is PS modification, where the oxygen atom is replaced by sulfur.^230^ This modification can improve the resistance of nucleic acids to nucleases, enhance blood stability, reduce renal clearance, and prolong the circulation time of drugs in vivo by improving their binding to plasma proteins.^231–233^ However, it has been determined that while improving stability, PS modification can induce inflammatory responses and produce hepatotoxic effects.^231^ Fomivirsen, a representative drug modified by PS, was withdrawn due to its limited therapeutic effect and inflammatory reaction.^234^ This has inspired the exploration of new modification technologies to reduce the adverse effects associated with PS modification.

#### Ribose modification

Ribose modification is another common strategy that has had notable success. Changes to the 2’ position group can affect nucleic acid stability and affinity,^227^ with common modifications including 2’-fluoro (2’-F), 2’-O-methoxyethyl (2’-MOE), and 2’-O-methyl (2’-OMe).^17,228,235^ These modifications considerably increase nuclease tolerance and prolong the half-life of nucleic acids while effectively avoiding the inflammatory reactions triggered by PS modification, demonstrating a higher safety and activity. Furthermore, synergistic modifications combining PS and 2’-MOE have considerably improved the physicochemical properties and reduced NADs side effects. Dual modifications, such as 2’,4’- and 2’,5’-sugar modifications, have been employed in the development of siRNA therapeutics to further expand the potential of ribose modifications.^236^ Beyond specific site modifications, altering the sugar ring provides another NADs design strategy.^237^ A locked nucleic acid (LNA) is a nucleic acid analog with a unique bicyclic structure, where the C4’ and O2’ atoms are connected by different methylene bridges, forming a stable C3’-endo conformation.^238^ An LNA adheres to Watson-Crick base pairing and has a strong affinity for DNA and RNA. However, owing to its bicyclic backbone structure limitations, an LNA can only spontaneously form A-type hybrid duplexes with target nucleic acid strands.^239,240^ These hybrids have strong thermal stability and can activate RNase H degradation activity under specific conditions, indicating LNA’s potential as an antisense drug. Overall, LNA’s good stability, high binding specificity, and strong nuclease resistance make it advantageous for in vitro and in vivo applications.^17^ In contrast, unlocked nucleic acid (UNA) is a flexible RNA mimic that lacks chemical bonds between the ribose ring’s C2’ and C3’ atoms. A UNA can attach to oligonucleotide monomers to form hybrids, regulating their flexibility and thermal stability.^241^ A UNA supports RNase H activity, which is beneficial for antisense-based nucleic acid therapies.^242^ Additionally, UNA modification at the siRNA terminus improves siRNA stability and silencing efficacy while considerably reducing off-target effects, highlighting its potential in the development of new therapeutic siRNAs.^243,244^

#### Nucleobase modification

Nucleobases are essential components of nucleic acids, and changes to their structure can affect the stability, biological activity, and immunogenicity of nucleic acids.^245,246^ By modifying specific sites on nucleobases, the stability and affinity of nucleic acids can be greatly improved. Canonical nucleoside analogs formed from nucleobase modifications include 5-methylcytidine (m5C), 5-fluorouracil (5-FU), N7-methylguanosine (m7G), pseudouridine (Ψ), N6-methyladenosine (m6A), and 2’-deoxy-2’-fluoro-uridine (2’-FU).^247–250^ Modifications, such as m5C and Ψ, reduce the activity of cytokines and biomarkers in dendritic cells, helping mRNA evade the immune system.^49,251,252^ Yoshida et al. found that base modification could considerably reduce the hepatotoxicity of gapmer ASO,^245^ providing insights for developing new gapmer ASOs. Additionally, in RNAi processes, the 5’ nucleobase affects the binding activity of AGOs to siRNA, thus reducing target cleavage activity.^253,254^ Therefore, chemical modification at this specific position could improve siRNA binding affinity.^225^

Nucleic acid analogs offer new modification technologies, considerably enhancing the stability and reducing the immunogenicity of NADs in vitro and in vivo.^255,256^ Replacing the phosphate backbone by other group improves target affinity, nuclease resistance, and pharmacokinetic properties of nucleic acids. For peptide nucleic acids (PNAs), the sugar-phosphate backbone is replaced by a peptide backbone, which retains the specific binding ability with DNA/RNA and offers higher affinity and better stability than natural nucleic acids.^257^ Due to these backbone modifications, PNAs show improved resistance to nuclease and protease digestion.^258^ These characteristics make PNAs powerful tools for disease diagnosis and treatment.^259,260^ However, their poor distributions in vivo and low cellular uptake are challenges for their clinical application. Phosphorodiamidate morpholino oligonucleotides (PMOs) are another class of nucleic acid analogs with significant potential, which are characterized by a six-membered morpholine ring backbone.^261^ Several PMO-based NADs have been approved for treating DMD, including eteplirsen (Exondys 51®),^262^ golodirsen (Vyondys 53^®^),^80^ viltolarsen (Viltepso^®^),^263^ and casimersen (Amondys^®^).^264,265^ Both PMOs and PNAs are neutral nucleic acid analogs with weak binding to plasma proteins, allowing them to be easily cleared by the kidneys.^266^ In practice, high doses are required to maintain the therapeutic effects, which can lead to corresponding toxicities and side effects.^267^

### Delivery systems

#### LNPs

**Fig. 4:**
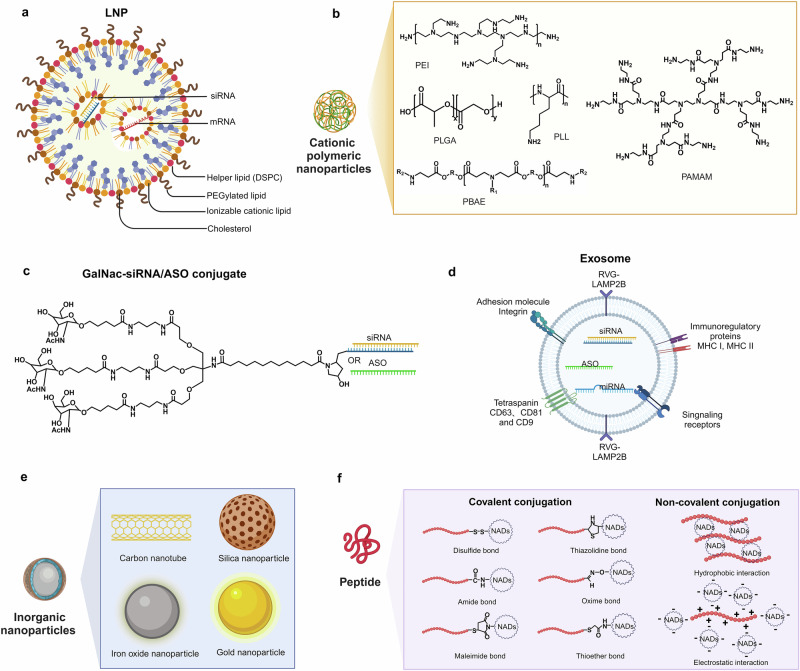
Chemical structure of NADs delivery systems. **a** There are four types of LNPs: ILs (or CLs), auxiliary lipids, cholesterol, and PEGylated lipids. **b** Schematic and molecular structural formula of cationic polymeric nanoparticles. **c** Triantennary GalNAc moiety conjugated to siRNA or ASO. **d** Engineered exosome with RVG-LAMP2B displayed on the outer surface. The exosome contains therapeutic nucleic acids, such as siRNA, microRNA, and ASO. **e** Schematic of inorganic nanoparticles. **f** Peptide-assisted NADs delivery strategies. The methods of covalent conjugation include disulfide, amide, maleimide, thiazolidine, oxime, and thioether bond. The methods of non-covalent complexation include hydrophobic and electrostatic interactions

LNPs are self-assembled nanostructures with diameters of approximately 100 nm, capable of combining with negatively charged nucleic acids through electrostatic interactions. They have been widely used for NADs delivery due to their excellent compatibility with cell membranes.^268,269^ Classical LNPs typically include ionizable lipids (ILs) or cationic lipids (CLs), auxiliary lipids, cholesterol, and Polyethylene glycol (PEGylated) lipids (Fig. 4a).^270^ These components self-assemble into monodisperse nanoparticles in specific proportions through intermolecular interactions, encapsulating NADs in their core to protect them from nuclease degradation during delivery. Furthermore, modifying the surface properties of LNPs can enhance the uptake by specific cells and alter the distribution of NADs.^271^

In early designs, CLs were permanently charged to bind the cell membrane and NADs effectively. However, their cytotoxicity limited their application in nanoparticle design.^272,273^ ILs are crucial for delivery efficacy, as they can encapsulate nucleic acids in LNPs while affecting the uptake and endosomal escape of NADs. ILs remain neutral at physiological pH, reducing the toxicity and immunogenicity of drugs. At low pH values, ILs acquire a positive charge, interacting with the negatively charged endosomal membrane and transforming its planar bilayer structure into a more hexagonal configuration. This transformation promotes the escape and release of NADs encapsulated in LNPs.^274,275^ 1,2-Dioleyloxy-3-dimethylaminopropane (DODMA) and its analog 1,2-dioleyloxy-3-(dimethylamino) propane (DODAP) were among the first ILs used for RNA delivery.^276^ Continuous optimization of ILs revealed that the pKa of ILs is a critical factor in delivery efficacy. An optimal pKa value of 6.2–6.5 maximizes NADs efficacy.^277^ Researchers have synthesized ILs, such as DLin-KC2-DMA and DLin-MC3-DMA, with the latter used as a novel component for encapsulating nucleic acids. DLin-MC3-DMA was integral to the development of the first FDA-approved siRNA drug, Onpattro®, and has been proven effective for mRNA delivery in vivo.^278,279^ In addition, this technology is used in the clinically approved vaccines ALC-0315 (BNT162b2-Comirnaty®) and SM-102 (mRNA-1273-Spikevax®), where the pKa value ranges from 6.1 to 6.7.^278,280^ Unlike DLin-MC3-DMA, these vaccines incorporate an ester-based structure in the hydrophobic tail of lipids, enabling faster lipid clearance and improving product tolerance.^281^

PEGylated lipids are another crucial component of LNPs that significantly influence their size, stability, and in vivo distribution.^282^ However, they have encountered several challenges.^283–285^ PEG modification can inhibit the binding of apolipoprotein E to LNPs, a key mechanism for LNP uptake by the liver, thereby affecting liver uptake of LNPs. Moreover, repeated administration of PEGylated products may produce PEG antibodies, potentially reducing drug efficacy.^286,287^

1,2-Distearoyl-sn-glycero-3-phosphocholine (DSPC) and 1,2-dioleoyl-sn-glycero-3-phosphoethanolamine (DOPE) are common helper lipids in commercially available LNPs. DSPC, a phospholipid molecule with a choline head group and two saturated octadecyl chains, is a critical excipient in Onpattro® and COVID-19 mRNA vaccines.^288^ Alternatively, DOPE disrupts the endosomal membrane structure by forming a hexagonal crystalline phase, facilitating the release of encapsulated RNA from endosomes.^275^ Addressing the limitations of traditional phospholipid structures, Liu et al.^289^ developed hundreds of ionizable phospholipids called iPhos, which were capable of promoting endosomal membrane fusion and inducing hexagonal phase transitions. Cholesterol possesses a strong membrane fusion capacity, facilitating the internalization of NADs and their entry into the cytoplasm. Studies indicate that apolipoprotein E can cause cholesterol to migrate from the core of LNPs to the outer lipid layer, potentially altering the surface properties of LNPs.^290,291^ Additionally, Siegwart’s team introduced the fifth component, selective organ targeting, to achieve precise and universal extrahepatic-targeted mRNA delivery strategies.^292^

Approximately 150 molecules employ LNP technology, with nearly 75% dedicated to delivering NADs in RNA therapy, including ASOs, siRNA, and mRNA.^293,294^ Several new RNA therapeutics are in late-stage clinical trials, such as mRNA-1944 (NCT03829384)^295^ and NTLA-2001 (NCT06128629).^296^ Vaccines, such as BNT162b2 and mRNA-1273, have been developed based on mRNA LNP technology against COVID-19, which demonstrate the feasibility of LNPs in delivering mRNA encoding the spike protein of syndrome coronavirus 2 (SARS-CoV-2), thus eliciting an immune response against the virus.^297^

#### Cationic polymeric nanoparticles

Delivery systems for NADs based on synthetic or natural cationic polymers are complex structures formed through electrostatic interactions between polymers carrying cationic groups and NADs. Upon cell internalization via endocytosis mechanisms, these nanoparticles disrupt endosomal membranes through the proton sponge effect, facilitating the intracellular delivery of exogenous nucleic acids. Various cationic polymers, including polyethylenimine (PEI), polyamidoamine dendrimers (PAMAM), poly-L-lysine (PLL), poly-β-aminoester (PBAE), and poly-lactic-co-glycolic acid (PLGA), have been developed for this purpose (Fig. 4b).^233,298^ As a representative cationic polymer, PEI is widely used in non-viral gene vectors in linear PEI (LPEI) or branched PEI (BPEI).^299,300^ BPEI exhibits stronger gene compression ability than LPEI due to its structure containing primary, secondary, and tertiary amine groups every two carbon atoms, enhancing electrostatic interactions with nucleic acids.^301^ With a molecular weight of 25 kDa, PEI is considered the “gold standard” for gene transfection due to its high efficiency, but it induces significant cytotoxicity and lacks biodegradability.^302,303^ PLL, another widely used cationic polymer, is synthesized through ring-opening polymerization of its monomers.^304^ Its positively charged amino acids interact electrostatically with the phosphate backbone of NADs, enabling the delivery of RNA and DNA.^305,306^ Despite the biodegradability and biocompatibility of PLL, its in vivo activity remains limited due to its high toxicity, low transfection efficiency, and poor endosomal escape ability. Functional modifications, such as chloroquine, histidine, PEG, or PEI, have been incorporated into PLL to address these limitations.^304,307,308^ Recent advancements include PLL nanoparticles coated with hyaluronic acid shells, significantly reducing cytotoxicity, enhancing cellular uptake, and improving gene expression.^305^

#### N-Acetylgalactosamine (GalNAc)

Most approved products based on bio-conjugated delivery systems use the GalNAc-coupled delivery system developed by Alnylam Pharmaceuticals. GalNAc, a carbohydrate compound, exhibits a high affinity for the asialoglycoprotein receptor (ASGPR).^309^ ASGPR is an endocytic receptor notably overexpressed on the hepatocyte membrane surface.^310,311^ Interaction between GalNAc and ASGPR facilitates the internalization of GalNAc-bound compounds from the cell surface into endosomes via clathrin-dependent receptor-mediated endocytosis.^312^ As the endosome matures and pH decreases, ASGPR dissociates from the GalNAc conjugate and recycles back to the hepatocyte surface.^313–315^ At the same time, GalNAc is degraded, releasing NADs into the cytoplasm to initiate gene regulatory activity.

Leveraging this mechanism, Alnylam has extensively explored GalNAc conjugation with NADs, particularly siRNA, to achieve liver-specific delivery (Fig. 4c).^312,316,317^ Notably, the nucleic acid molecules in these conjugates are directly exposed to serum; enhancing their stability in the physiological environment is a crucial challenge. Studies have demonstrated that stability can be significantly enhanced by extensive chemical modification at the 2’ position of nucleotide sugars and by replacing the phosphodiester bond with a thiophosphate bond.^318–320^ Furthermore, stability can be improved without compromising drug activity by optimizing the number and modification positions of 2’-F and 2’-OME groups on both strands of double-stranded siRNA, leading to substantial efficacy improvements.^319,321,322^ These advancements have accelerated the shift from standard template chemistry to enhanced stability chemistry. Rapid ligand-receptor binding and efficient uptake by target cells are critical for receptor-targeted delivery systems to avoid in vivo clearance.^315,323–325^ Multivalent ligands show significantly enhanced binding affinity for ASGPR compared with monovalent GalNAc units, with affinity rankings in the order of tetraantennary > triantennary » biantennary » monoantennary.^326^ Studies by Biessen^327^ have suggested that a 2-nm interval between GalNAc and dendritic branch points may optimize NADs transportation efficacy. Additionally, hydrophobic linkers have been found to enhance GalNAc–ASGPR interactions.^328^

With the success of Alnylam in GalNAc, other companies have focused on RNA therapy and developed their delivery systems. Dicerna Pharmaceuticals has made considerable efforts to advance GalNAc delivery technology by adopting tetraantennary GalNAc conjugates (GalXC) for NADs delivery. In this conjugate, a unique four-ring structure is introduced on the passenger chain, enhancing the stability of the conjugate.^310,329^ Additionally, it accurately targets multiple GalXC ligands and successfully delivers siRNA to hepatocytes. Many candidates that involve this technology are currently undergoing clinical evaluation. Nedosiran,^330^ a siRNA targeting the silencing of lactate dehydrogenase A in hepatocytes (LDHA) with four covalently linked GalNAc, was approved by the FDA in 2023.

#### Exosomes

Exosomes are vesicle-like structures with a diameter of 40–160 nm released after the fusion of intracellular multivesicular bodies (MVBs) with the cell membrane.^331,332^ As a bridge of intercellular communication, classical exosomes have a monolayer structure, which can encapsulate bioactive substances, such as proteins, lipids, DNA, and RNA, in the core and deliver them to effector cells to play specific biological functions (Fig. 4d).^333–336^ They have been considered good candidates for NADs delivery.^337–340^ For example, Kaban et al.^341^ used exosomes derived from natural killer (NK) cells as a carrier of siRNA targeting BCL-2 to treat patients with estrogen receptor-positive (ER + ) breast cancer, resulting in enhanced apoptosis of breast cancer cells. Additionally, another study used plasma exosomes to deliver siRNA to T cells and monocytes and caused post-transcriptional gene silencing in recipient cells.^342^ Furthermore, exosomes can easily cross biological barriers, such as the blood-brain barrier (BBB), which has good application potential in extra-hepatic-targeted NADs delivery.^343–345^

When exosomes are used as NADs delivery vehicles, it is essential to consider the strategy of efficient drug loading. As a natural barrier of exosomes, the membrane structure of the lipid bilayer can protect NADs from external influences. Still, the existence of the membrane structure makes it difficult for exosomes to load drugs efficiently. The strategies of drug loading by exosomes are mainly divided into two categories.^346–348^ The first is the exogenous route, in which the NADs are directly introduced into the obtained exosomes via electroporation, co-incubation, sonication, extrusion, and freeze-thaw cycling.^349–352^ Although cargo uploading exogenously is simple and convenient to operate, the integrity of exosomes may be damaged during the loading process, which may affect the effect and require additional purification steps to remove the unloaded drugs.^353,354^ The other method is the endogenous pathway, which uses the endogenous pathway of exosome generation to indirectly improve the production of exosomes by promoting the expression of target nucleic acids and exosome secretion in productive cell lines.^355^ However, due to our lack of understanding of exosome biology, structure, and biogenesis, the strategy of endogenous drug loading still requires further research and optimization.

One common disadvantage of exosomes is their random movement in vivo and lack of specific targeting.^355,356^ The abundant lipids and membrane-bound proteins on the surface of exosomes provide binding sites for targeting ligands, such as peptides, antibodies, and aptamers. These ligands can be stably attached to the surface of exosomes through covalent bonds to enhance their targeting ability. For example, Kim et al.^357^ modified exosomes with transferrin receptor-binding peptide (T7 peptide), which efficiently delivered AMO-21 into glioblastoma (GBM) cells in vitro. Furthermore, in vivo delivery results have shown that T7 peptide-modified exosomes effectively reduced the level of miR-21 in tumor cells and inhibited tumor growth compared with unmodified exosomes. Another study used a single-chain variable fragment (scFv) to modify exosomes derived from human umbilical cord blood mesenchymal stem cells (MSCs).^358^ In addition, exosomes can be genetically engineered to express ligands on their surfaces simultaneously. The most commonly used exosome surface protein is lysosome-associated membrane protein (LAMP). The N-terminus of LAMP-2B is located on the surface of exosomes and can specifically target given sequences.^359–362^ After screening for cell-specific binding peptides for particular organs or tissues, such as the rabies virus glycoprotein peptide, LAMP-2B can be genetically modified to achieve targeted delivery (Fig. 4d).

Thus far, approximately 40 companies worldwide, including Codiak Biosciences, Evox Therapeutics, Tavec Pharmaceuticals, Carmine Therapeutics, Anjarium, and Micromedmark Biotech, have developed exosome-based therapies, which are expected to provide cost-effective and more accurate targeted therapies in the clinic (https://bioinformant.com/companies-developing-exosome-technologies/). However, challenges, such as large-scale production, purity, and batch homogeneity of exosomes, still limit their clinical application. Additionally, there are no regulations for the control of therapeutic drugs based on exosomes that consider safety, effectiveness, and quality control, highlighting the urgent need for standardized methods and principles to manage these molecules.^363^

#### Inorganic nanoparticles (INPs)

INPs are nanocarriers based on inorganic substances that have attracted considerable attention due to their unique electrical and optical properties, biocompatibility, and low cytotoxicity. Commonly used INPs include gold nanoparticles (AuNPs), silica nanoparticles (SiNPs), magnetic nanoparticles (MNPs), and carbon nanotubes (CNTs) (Fig. 4e).^364–366^ Unlike other delivery carriers, INPs possess a stable and robust structure, a large specific surface area, and tunable surface properties, allowing precise control of the drug delivery process through surface functionalization and controlled release modifications.

AuNPs are widely developed INPs with excellent biocompatibility and low toxicity. Their flexible surfaces enable nucleic acids to bind to the gold nanoparticles directly.^367,368^ For example, Shrestha et al.^369^ developed a gold nanoparticle-mediated drug delivery platform for the co-delivery of doxorubicin and polo-like kinase 1 (Plk1) siRNA, offering an adaptable and straightforward platform for studying drug-siRNA combinations in cancer treatment. Notably, NU-0129, composed of siRNA targeting the GBM oncogene Bcl2Like12 (Bcl2L12) and a gold nanoparticle core, was the first spherical nucleic acid (SNA) drug administered systemically.^370^ The Phase 0 clinical study (NCT03020017) in eight patients with GBM showed that NU-0129 could pass through the BBB and accumulate in tumors, reducing the abundance of BCL2L12 protein and demonstrating its potential as an innovative therapy for GBM.^371^

MNPs are typically made of magnetic materials, such as iron oxide or iron platinum, and are usually coated with biocompatible materials to enhance their stability and biocompatibility. Under the influence of an external magnetic field, therapeutic drugs or molecules can be loaded onto magnetic nanoparticles and targeted to precise regions in the body.^372,373^ The unique superparamagnetic nature, lower toxicity, and site-specific targeting capabilities of MNPs make them excellent nanocarriers for NADs delivery.^374^

CNTs are cylindrical structures composed of a hexagonal arrangement of sp2 hybridized carbon atoms, also known as graphene.^375^ CNTs can be categorized into single-walled CNTs (SWCNTs) and multi-walled CNTs (MWCNTs). SWCNTs consist of a single layer of graphene sheets rolled seamlessly into a cylindrical tube. In contrast, MWCNTs are composed of multiple graphene layers wrapped around each other in a cylindrical shape. Although CNTs have poor solubility in both water and organic media, they can be chemically modified to improve their solubility, degradation ability, and drug-loading capacity while reducing toxicity.^376^ This simple surface functionalization has made CNTs promising carriers for NADs delivery in various diseases.^377^

Moreover, SiNPs are considered excellent carriers for NADs delivery.^378^ Research has shown that porous silicon nanoparticles (pSiNPs) can replace commonly used viral vectors or lipid transfection reagents as novel vectors for delivering siRNA to dendritic cells.^379^ Luo designed pSiNPs with ASO as targeted gene and drug delivery platforms for GBM treatment. These pSiNPs penetrate the BBB monolayer in vitro and target the brain after intravenous injection in an in situ GBM mouse model. This indicates that pSiNPs and their multifunctional strategies have strong potential for cancer treatment and gene delivery research.^380^

#### Peptides

**Fig. 5:**
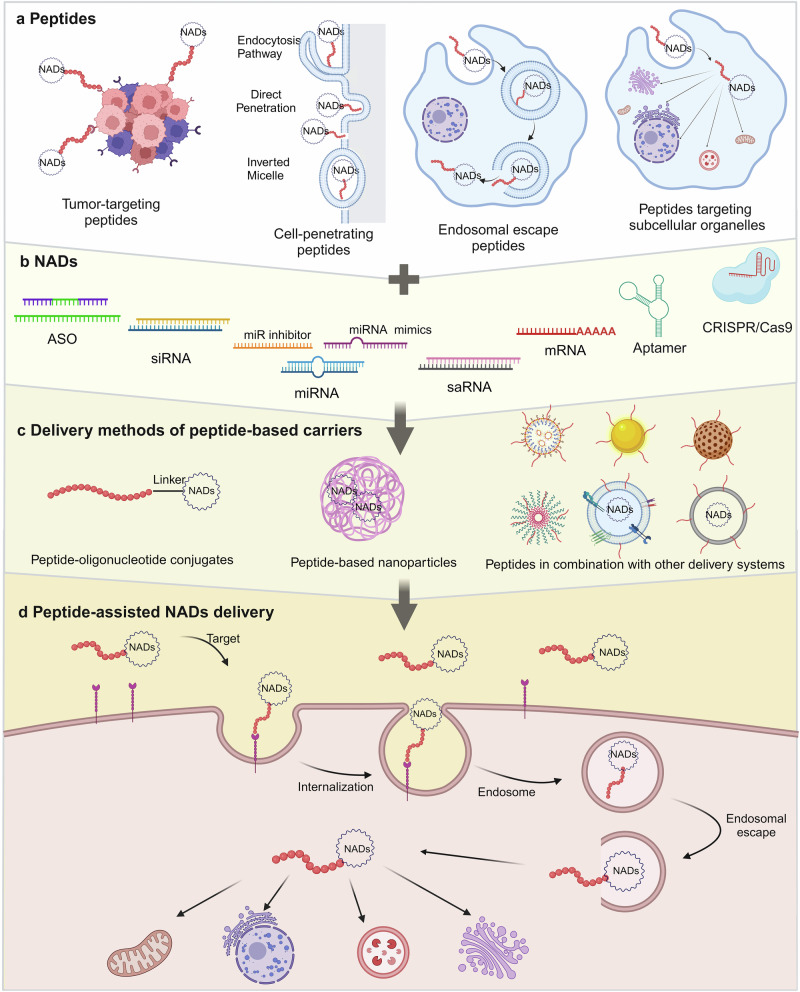
Schematic of peptide-assisted NADs delivery. **a** Classes of peptides facilitating the delivery of NADs across biological barriers. **b** Classes of NADs. **c** The delivery methods of peptide-based carriers include peptide–oligonucleotide conjugates, peptide-based nanoparticles, and peptides in combination with other delivery systems. **d** Peptides mediate the entry of NADs into cells and transfer them across the cell membrane, complete endosomal escape, and eventually release NADs in the cytoplasm, mitochondrion, nucleus, endoplasmic reticulum, lysosomes, and Golgi apparatus

Peptides comprise fewer than 100 amino acid residues, and they are between small molecules and proteins in size. Historically, peptides have been recognized for their diverse roles as hormones, signaling molecules, carriers, and supplements.^381–384^ They are easily synthesized, possess relatively stable chemical properties, and exhibit a high selectivity. Due to their low immunogenicity and strong specific targeting capacity, peptides have emerged as promising carriers for selectively delivering NADs through various modalities (Fig. 4f).^385,386^ Peptides exhibit multiple functions, including tissue targeting,^387–389^ membrane penetration,^390–392^ endosome escape,^393–395^ and nuclear localization,^396–398^ depending on their amino acid composition. Several peptides have been developed specifically for NADs delivery based on these unique properties for cell targeting, penetration, endosomal escape, and sub-organelle targeting (Fig. 5).^25,399–403^

##### Targeting peptides

Nonspecific cell binding is the leading cause of the off-target effects observed with several therapeutic molecules. The arginine-glycine-aspartic acid (RGD) sequence has been extensively applied in tumor-targeted therapy, mainly targeting αvβ_3_ receptors overexpressed on several tumor cells.^404,405^ A recent study demonstrated that siRNA conjugated to cyclic RGD (cRGD) could selectively enter cells that express αvβ_3_ integrin.^406^ Intravenously injected cRGD-siRNA molecules produced no innate immune response in mice with transplanted tumors.^407^ Additionally, researchers have found that incorporating siRNA into PEG-modified polylysine with cRGD can enhance gene silencing ability, improve cell uptake, inhibit glioma angiogenesis, and delay tumor progression after systemic administration in mice.^408^

The acidic extracellular microenvironment has become an effective disease diagnosis and treatment target.^409^ pH low insertion peptides (pHLIPs), derived from the C-helix of bacteriorhodopsin, can sense the pH value near the plasma membrane and deliver drugs to pathological tissues.^410^ In acidic environments, the elevated proton concentration increases the hydrophobicity of peptides. pHLIPs spontaneously fold to form α helices, which insert and cross the cell membrane to transport conjugated NADs into cells.^191,411^ In anti-tumor therapy, it has been demonstrated that pHLIPs can deliver PNAs to cancer cells due to their natural targeting abilities.^412,413^ For example, in a mouse model of lung adenocarcinoma, the delivery of PNAs targeting CEACAM6 using pHLIPs resulted in CEACAM6 gene silencing and tumor growth inhibition.^414^

##### Cell-penetrating peptides (CPPs)

CPPs are a short peptide class consisting of 5–30 amino acids that can cross the cell membrane directly.^399^ They can penetrate the cell membrane by themselves and effectively enhance the intracellular delivery of NADs, aiding their interaction with the target to achieve therapeutic goals.^415–417^

Despite their significant therapeutic potential, CPPs have some limitations. One major issue is their lack of selectivity, as they can carry NADs into almost all cell types. To address this, researchers have developed strategies such as local administration, targeting ligands on CPPs, or using activatable CPPs that penetrate only under specific biological stimuli.^418,419^ Ensuring the stability and biological activity of CPPs during the delivery process is also a challenge. Modifications can be made to the termini of existing CPPs or the peptide backbone can be adjusted, such as through peptide cyclization, to improve stability and cell permeability.^420,421^ Another limitation of CPPs is their limited clinical efficacy, often requiring high concentrations and doses to achieve a therapeutic effect, which can cause significant toxicity and side effects, hindering the development of clinical applications. Current research has found that inducing peptides to form multimers and using multifunctional fusion peptides can improve the delivery efficiency of CPPs to some extent.^422–424^

##### Endosomal escape peptides

Endosomal escape is a significant challenge for non-viral NADs delivery systems. Typically, after arriving at a specific cell, NADs pass through the cell membrane and reach the cytoplasm through various mechanisms, such as direct penetration or endocytic uptake.^309,422^ However, peptide carriers and their cargos often become trapped in acidified endosomes, preventing their entry into the cytoplasm, nucleus, and other subcellular compartments.^14,425^

Fusogenic peptides are short peptides that promote endosomal release by enhancing interactions with endosomal membranes.^426,427^ In a low pH environment, fusogenic peptides undergo pH-dependent conformational changes to form α-helices, which insert into the endosomal membrane, causing instability and decomposition of the membrane structure. This allows internalized NADs to dissociate from the fusogenic peptide delivery system and escape into the cytoplasm to exert their therapeutic effects.^427^ Many fusiform peptides of viral and non-viral origin, such as the INF7 family,^428^ L17E family,^429^ GALA/KALA family,^430–432^ and HA family,^433^ can disrupt endosomal membranes and increase cytoplasmic delivery. One study evaluated the ability of the H5WYG peptide to deliver NADs and promote endosomal release, confirming the elevated endosomal escape function of this clostridial peptide.^434^ Many studies have shown that self-assembled peptide carriers modified with fusogenic peptides can overcome intracellular delivery obstacles.^435,436^ For instance, the fusogenic peptide L17E was linked to peptide self-assembled disks using click chemistry. Compared with unmodified disks carrying plasmid DNA, nanodisks modified with L17E demonstrated enhanced endosomal escape and improved transfection efficiency in cell culture.^429^

##### Peptide-targeting subcellular organelles

The structural integrity and functional stability of organelles are essential for maintaining normal cellular physiological functions. Dysfunction of these organelles can lead to various diseases, including diabetes, neurodegenerative diseases, CVDs, and cancer, making them potential therapeutic targets.^437–439^ As mentioned earlier, the efficacy of NADs therapy largely depends on the efficient and safe delivery of NADs to their specific action sites within the body.^14^ After escaping from endosomes, the next challenge is transporting NADs quickly and effectively to the nucleus or specific organelles.^425^ Peptide targeting of subcellular organelles offers a promising strategy to guide NADs to dysfunctional organelles, thus achieving optimal therapeutic effects. This approach provides a potential method to overcome the intracellular delivery obstacles and enhance the efficacy of NAD-based therapies.

##### Multifunctional peptides

In previous clinical trials, using individual functional peptides for NADs delivery showed limited efficacy due to their inability to overcome various delivery obstacles.^440^ To address this issue, researchers have integrated different peptides or functional domains capable of overcoming transfer barriers into a single peptide-based carrier to enhance delivery efficiency.^441–444^ Compared with individual functional peptides, multifunctional peptide carriers considerably improve transfection efficiency and have demonstrated outstanding potential in clinical applications.^445–447^ For example, amino acid pairing (AAP) peptides are a novel class of self-assembled biomolecules comprising two main structural domains: an amino acid pairing domain and a cell permeability domain.^448^ AAP peptides possess recognition and membrane-targeting functions, facilitating gene delivery through interactions with DNA and siRNA.^448^

The discovery of peptide-based NADs delivery systems is accelerating by the use of various peptide combinations and high-throughput screening, providing new directions for optimizing and developing peptide carriers.^449–451^ For example, Li et al.^452^ designed a multifunctional peptide vector (PLD-R9-G-NLSW) containing the CPP R9, NLS, and 2,3-dimethylmaleic anhydride-modified PLL. This vector condenses with the pIRES-VEGF plasmid to form a complex for gene delivery. In vitro results showed that the complex considerably improved gene internalization and transfection efficiency while reducing cytotoxicity. However, researchers have discovered that the immediate coupling of individual peptides can affect the function and activity of the units. Given the likelihood that distinct peptides may interact with each other during the fusion process, the final functional characteristics of the peptides may not involve a simple integration.^12,453^ This phenomenon suggests that the interaction mechanism between combined peptides and the structure-activity relationship needs to be explored in more detail.

Recently, peptides have been applied as ligands to achieve delivery functions in complexes known as peptide-oligonucleotide conjugates (POCs).^454,455^ In this approach, oligonucleotides or their analogs are covalently linked to one or more peptide residues to form POCs. Various methods, including disulfide bonds, thioether bonds, thiol-maleimide bonds, phosphodiester bonds, and click chemistry, are used for this covalent connection.^456–458^ The primary advantage of covalent conjugation is that the resulting product is a single compound with defined structural and stoichiometric characteristics, aligning with the ideal properties for drug design and in vivo application.^459,460^ However, covalent conjugation also has drawbacks. Interactions between cationic peptides and negatively charged oligonucleotides can enhance toxicity, affect pharmacokinetic and pharmacological properties, and limit the efficacy of these therapies.^461^ Additionally, the interaction between anions and cations can complicate large-scale preparation and purification of these conjugates.^462^

The most advanced application of POCs is the delivery of charge-neutral oligonucleotides, such as PNA and PMO, using CPPs.^463–465^ CPP-PNA conjugates with Tat and R8 have been successfully applied to PNA delivery^466,467^ and showed sound therapeutic prospects in anti-virus, antibacterial, and anti-inflammatory therapy. Beyond CPPs, other functional peptides have also been exploited for PNA delivery. For instance, Soudah et al.^468^ used a conjugate of the cytoplasmically localized internalized peptide (CLIP6) and PNA to treat GBM cells, considerably upregulating the tumor suppressor Mnk2a and promoting cancer cell death. Kaplan et al.^457^ showed that pHLIP-αKu80 (γ), which was created by covalently conjugating pHLIPs with PNA targeting KU80 via disulfide bonds, could selectively reduce Ku80 expression under acidic conditions. In mice, intravenous injection of fluorescently labeled pHLIP-αKu80 (γ) targeted tumors and reduced Ku80 expression.

The development of peptide-phosphoryl diamine morpholino oligonucleotide (PPMO) conjugates has further advanced NADs delivery, particularly for neuromuscular diseases, such as spinal muscular atrophy (SMA) and DMD.^469,470^ A recent study demonstrated that subcutaneous injection of a CPP-DG9 and PMO conjugate improved muscle strength and innervation in mice with severe SMA, significantly extending their median survival with no apparent side effects.^471^ To enhance the delivery of neutrally charged oligonucleotides, Gait and Wood^472^ developed a series of arginine-rich CPPs named [PNA/PMO]-internalized peptides (Pip), including Pip2a, Pip2b, Pip5e, and Pip6a. Studies have shown that Pip6a-PMO effectively rescues the disease phenotype and improves the survival rate of severe SMA mice, showing strong efficacy in the central nervous system and peripheral tissues.^473,474^ Pip6a-PMO predicts a potent therapeutic option that combines the genetic precision of SSO with the systemic delivery efficacy of peptides.^475^

With technological advances, various novel POCs have demonstrated the feasibility of delivering siRNA, ASO, and miRNA in vitro.^406,476^ Studies have found that bivalent cRGD successfully transports VEGF receptor 2 (VEGFR2)-siRNA into tumor cells in a mouse non-small cell lung cancer xenograft model, downregulating VEGFR2 expression and significantly inhibiting cancer progression.^406^ Kim et al.^402^ designed a dual-targeting drug delivery system for miR-21 inhibitors, consisting of a PDL1-binding peptide covalently linked with an anti-miR-21 inhibitor via a click reaction. Pep-21 treatment specifically silenced target miR-21 in B16 melanoma cells and M2 macrophages, reducing tumor cell migration and inhibiting tumor progression. PepGen developed a POC drug, PGN-EDO51, using its enhanced delivery oligonucleotide platform, which skips exon 51 in the DMD gene. In Phase I clinical trials, PGN-EDO51 showed good safety, tolerability, and efficacy, and Phase II trials are ongoing.

Electrostatic or hydrophobic interactions between peptides and NADs create self-assembling peptide-based nanoparticles.^477–479^ These complexes protect nucleic acids from nuclease degradation and reduce the likelihood of adverse reactions.^480^ Using this strategy, Ryu et al.^481^ developed an S-R11 fusion peptide (space peptide bound to polyarginine) that forms stable nanocomposites with siRNA molecules through electrostatic attraction and hydrogen bonding, facilitating siRNA delivery to difficult-to-transfect immune cells. Sirnaomics’ histidine-lysine copolymer peptide nanoparticle (PNP) delivery platform has demonstrated clinical therapeutic potential for RNAi therapy.^482^ In contrast, non-covalent strategies have been used for delivering siRNA, plasmid DNA, and splicing correction oligonucleotides.^483,484^ The peptide p5RHH has been efficiently used to transfect siRNA and mRNA into human cartilage to reduce chondrocyte apoptosis and prevent cartilage degeneration^485–487^ as well as into cardiac tissue for treating abdominal aortic aneurysm to reduce the risk of aortic rupture and sudden death in mice.^484^

## Clinical applications of NADs

**Fig. 6:**
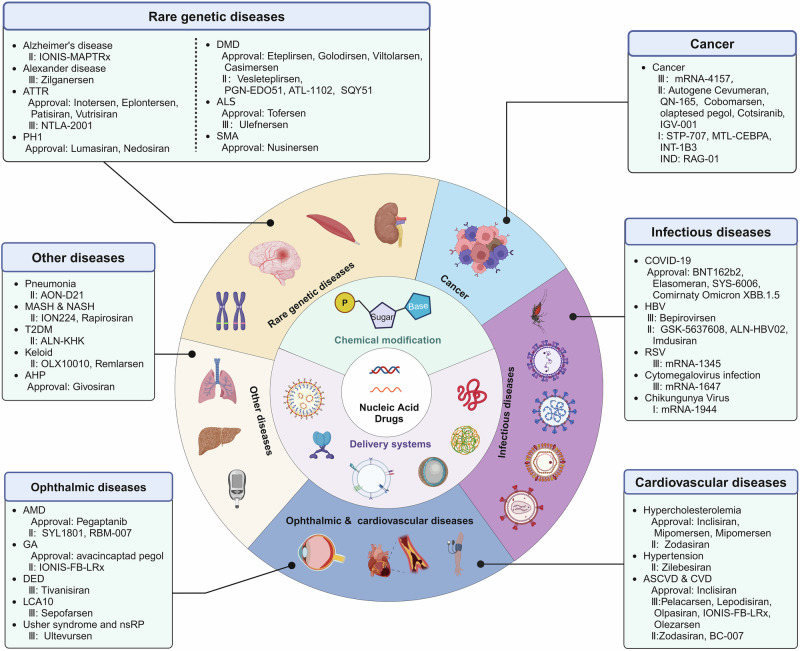
Clinical application of NADs. Several NADs have been adopted as vaccines for treating rare genetic diseases, cancer, ophthalmic diseases, cardiovascular diseases, and infection diseases, and they have shown remarkable therapeutic effects. The box summarizes the therapeutic NADs for various diseases in the clinic or on the market

Theoretically, NADs can cure any disease by allowing the selection of the correct nucleotide sequence on the target gene. Unlike conventional therapeutics, NADs induce long-lasting or curative effects due to their distinct physicochemical and biological properties. Numerous NADs have transitioned from bench to bedside and have successfully been approved for clinical trials (Fig. 6). Several NADs, including ASOs, aptamers, siRNAs, and mRNA vaccines, have been adopted as vaccines for treating rare genetic diseases, cancer, ophthalmic diseases, CVDs, and infections. Table 2 summarizes NADs successfully applied in clinical experiments for treating human diseases.

**Table 2:** Status of NADs-based therapeutics clinical trials

Drug (alternate name)	Company	Classification	Indication (Phase)	Target	Modification & Delivery	Clinical Trail Number	Ref(s)
**Rare genetic disorders**							
NTLA-2001	Intellia Therapeutics	CRISPR-Cas9	ATTR-CM (III)	TTR	LNP	NCT06128629	^296,588^
Zilganersen (ION-373)	Ionis	ASO	Alexander disease (III)	GFAP mRNA	2’-MOE	NCT04849741	^589^
Fitusiran (ALN-AT3)	Sanofi	siRNA	Hemophilia A/B (III)	Serpin C1	GalNAc	NCT03417102 NCT03417245	^590^
Donidalorsen (IONIS-PKK-LRx)	Ionis	ASO	HAE (III)	PKK	GalNAc, 2’-MOE	NCT05392114	^591,592^
Ulefnersen (ION-363)	Ionis	ASO	ALS (III)	FUS	PS, 2’-MOE	NCT04768972	^506^
Cemdisiran (ALN-CC5)	Alnylam & Regeneron	siRNA	PNH, Myasthenia gravis (III), IgAN, (II)	Complement C5	2ʹ-OME,2’-OF & GalNAc	NCT05070858 NCT05744921 NCT05133531	^593,594^
Vesleteplirsen (SRP-5051)	Sarepta Therapeutics	ASO	DMD (II)	Exon 51 of DMD	PMO/ PPMO	NCT04004065	^500^
PGN-EDO51	PepGen	ASO	DMD (II)	Exon 51 of DMD	EDO	NCT06079736	^501^
ATL-1102 (ISIS-107248)	Antisense Therapeutics	ASO	DMD (II)	ITGA4	2’-MOE	NCT05938023	^595^
Zorevunersen (STK-001)	Stoke Therapeutics	ASO	Dravet Syndrome (II)	SCN1A	2’-MOE	NCT04740476 NCT04442295	^596^
IONIS-MAPTRx (BIIB-080)	Ionis & Biogen	ASO	Mild Alzheimer’s Disease (II)	MAPT	2’-MOE, PS	NCT05399888 NCT03186989	/
SQY51	Sqy Therapeutics	ASO	DMD (I/II)	Exon 51 of DMD	tricyclo-DNA	NCT05753462	/
NTLA-2002	Intellia Therapeutics	CRISPR-Cas9	HAE(I/II)	KLKB1	LNP	NCT05120830	^597^
mRNA-3705	Moderna	mRNA	MMA (I/II)	MUT	LNP	NCT05295433 NCT04899310	^598^
**Cancer**							
mRNA-4157 (V-940)	Merck & Moderna	mRNA	NSCLC, Melanoma (III)	/	LNP	NCT03313778 NCT06077760 NCT05933577 NCT03897881	^599^
Autogene Cevumeran (BNT-122)	BioNTech	mRNA	Colorectal, PDAC (II)	unspecified TAAs	LNP	NCT03289962 NCT03815058 NCT05968326	^600^
IGV-001	Imvax	ASO	GBM (II)	IGF type 1 receptor	PS, Goldspire™	NCT04485949	^520^
Cotsiranib (STP-705)	Sirnaomics	siRNA	Keloid, BCC, hypertrophic scars, isSCC (II)	TGF-β1、COX-2	PNP	NCT04669808	^482^
QN-165 (AS1411)	Qualigen Therapeutics	Aptamer	RCC, glioma and AML (II)	Nucleolin	/	NCT00881244 NCT01034410	^183,184^
Cobomarsen (MRG-106)	miRagen Therapeutics	miRNA	MF, CTCL, DLBCL, CLL, ATLL (I/II)	miR-155 inhibitor	LNA	NCT02580552 NCT03713320 NCT03837457	^125,126^
olaptesed pegol	TME Pharma	Aptamer	Tumor, GBM (I/II)	CXCL12	/	NCT03168139 NCT01486797	^601^
TTX-MC138	TransCode	miRNA	advanced solid tumors (I/II)	miRNA-10b inhibitor	iron oxide nanocarrier	NCT06260774 NCT05908773	^127^
STP-707	Sirnaomics	siRNA	solid tumors (I)	TGF-β1、COX-2	PNP	NCT05037149	/
MTL-CEBPA	MiNA Therapeutics	saRNA	HCC (I)	CEBPA	/	NCT02716012	^142–144^
RAG-01	Ractigen Therapeutics	saRNA	NMIBC (IND)	CDKN1A	/	/	^122^
**Ophthalmic diseases**							
Tivanisiran (SYL1001)	Sylentis, S.A.	siRNA	DED (III)	TRPV1	/	NCT05310422 NCT03108664	^528^
Sepofarsen (QR-110)	ProQR Therapeutics	ASO	LCA10 (III)	CEP290	2’-OME	NCT03913143	^602^
Ultevursen (QR-421a)	ProQR Therapeutics	ASO	Usher syndrome and nsRP (II/III)	USH2A	2’-OME	NCT05158296	^533^
SYL1801	Sylentis	siRNA	AMD (II)	NRARP	/	NCT05637255	^526^
RBM-007 (APT-F2)	Ribomic	Aptamer	wet AMD (II)	FGF2	/	NCT04640272 NCT04895293	^525^
IONIS-FB-LRx (RO7434656)	Roche & Ionis	ASO	GA (II)	Complement Factor B	2’-MOE & GalNAc	NCT03815825	^603^
**Cardiovascular diseases**							
Pelacarsen (AKCEA-APO(a)-L_Rx_)	Novartis AG	ASO	CVD (III)	ApoA	2’-MOE & GalNAc	NCT05900141 NCT04023552	^542,604^
Lepodisiran	Lilly	siRNA	ASCVD (III)	ApoA	2’-MOE, 2’-F & GalNAc	NCT06292013	^546^
Olpasiran (AMG 890)	Amgen Biopharmaceuticals	siRNA	ASCVD (III)	ApoA	2’-MOE & GalNAc	NCT04270760 NCT03626662 NCT05581303	^547,548^
IONIS-FB-LRx (RO7434656)	Roche & Ionis	ASO	Primary IgA Nephropathy (III)	Complement Factor B	2’-MOE & GalNAc	NCT05797610	/
Olezarsen (IONIS-APOCIII-LRx)	Akcea Therapeutics & Ionis	ASO	SHTG & FCS (III)	ApoC-III	GalNAc	NCT05681351 NCT05552326 NCT05079919 NCT05130450	^605,606^
Zodasiran (ARO-ANG3)	Arrowhead Pharmaceuticals	siRNA	HoFH (II)	ANGPTL3	2’-OME, 2’-F & GalNAc	NCT05217667	^607^
Zilebesiran (ALN-AGT01)	Alnylam	siRNA	Hypertension (II)	Angiotensinogen	2’-MOE, 2’-F & GalNAc	NCT06272487 NCT04936035 NCT05103332	^608^
MRG-110 (S-95010)	miRagen Therapeutics	miRNA	Chronic heart failure (I)	miR-92a Inhibitors	LNA	NCT03603431	^125^
**Infection diseases**							
mRNA-1345	Moderna	mRNA	Respiratory syncytial virus infection (III)	RSV fusion	LNP	NCT06067230	^609^
mRNA-1647	Moderna	mRNA	Cytomegalovirus infection (III)	HCMVgB & HCMVgH	LNP	NCT05085366	^610^
Bepirovirsen (GSK-3228836)	GSK & Ionis	ASO	HBV (III)	HBV RNA	2’-MOE	NCT05630807 NCT05630820	^559^
GSK-5637608 (JNJ-73763989)	GSK	siRNA	HBV (II)	HBV RNA	GalNAc	NCT05275023 NCT0512359	^560^
ALN-HBV02 (VIR-2218)	Alnylam & Brii Biosciences Ltd & Vir Biotechnology	siRNA	HBV (II)	HBV RNA	2’-OME, 2’-F & GalNAc	NCT05612581	^561^
Imdusiran (ARB-270729)	Arbutus Biopharma	siRNA	HBV (II)	hepatitis B surface antigen	2’-F & GalNAc	NCT06154278 NCT06245291 NCT06277037	^562^
mRNA-1944	Moderna	mRNA	Chikungunya Virus (I)	CHKV-24	LNP	NCT03829384	^295^
**Other diseases**							
Sapablursen (IONIS-TMPRSS6-LRx)	Ionis	ASO	PV (II)	TMPRSS6	2’-MOE & GalNAc	NCT05143957	^611^
CWT001 (TenoMiR)	Causeway Therapeutics	miRNA	Lateral epicondylitis (II)	microRNA-29a mimic	2’-OME, 2’-F	NCT06192927	/
ION224	Ionis	ASO	MASH (II)	DGAT2	2’-MOE & GalNAc	NCT04932512	^612^
Rapirosiran (ALN-HSD)	Alnylam	siRNA	NASH (II)	HSD17B13	2ʹ-MOE, 2’-F & GalNAc	NCT05519475	/
ALN-KHK	Alnylam	siRNA	T2DM (I/II)	KHK	GalNAC	NCT05761301	/
OLX10010 (BMT-101)	Olix Pharmaceuticals	siRNA	Hypertrophic scars (II)	CTGF	2’-MOE	NCT04877756 NCT03569267	/
Remlarsen (MRG-201)	miRagen Therapeutics	miRNA	Keloid disorder (II)	microRNA-29 mimic	/	NCT03601052	^124^
AON-D21	Aptarion Biotech	Aptamer	Community-acquired pneumonia (II)	C5a	/	NCT05962606	^613^

### Rare genetic diseases

Transthyretin amyloidosis (ATTR) is a rare systemic disease characterized by the progressive deposition of misfolded TTR protein in the heart and peripheral nerves, leading to transthyretin amyloid polyneuropathy (ATTR-PN) and transthyretin amyloid cardiomyopathy (ATTR-CM).^488,489^ Several NADs have been approved for ATTR treatment, including ASOs, such as inotersen^490,491^ and eplontersen,^492^ as well as siRNAs, such as patisiran^97^ and vutrisiran.^493^ These therapies work by disrupting the relevant mRNA, thus inhibiting TTR synthesis. Additionally, researchers are exploring new methods to block TTR production via gene-editing techniques. For instance, a Phase III clinical trial evaluated the efficacy and safety of NTLA-2001, a CRISPR-Cas9-based therapy, in participants with ATTR-CM. In a previous Phase I trial, six patients treated with a single dose of NTLA-2001 showed significant reductions in serum TTR protein levels, with no serious adverse effects or liver damage reported.^494^

DMD is a rare genetic muscle disorder caused by mutations in the dystrophin gene, leading to the absence of the dystrophin protein. Patients typically exhibit symmetric, progressive muscle weakness and atrophy, ultimately leading to premature death due to respiratory and cardiovascular complications.^495^ Significant progress has been made in DMD therapeutic strategies, including NADs. Approved ASO drugs target exon 45 (casimersen),^264,496^ exon 51 (eteplirsen),^497,498^ and exon 53 (golodirsen and viltolarsen)^80,263^ to induce specific exon skipping in the dystrophin gene, thus delaying disease progression. Researchers have developed second-generation skipping drugs, called PPMOs, to enhance targeting and skipping activity.^499^ Sarepta’s SRP-5051, an ASO conjugated with a targeting peptide, has shown higher levels of dystrophin expression and exon skipping rates than eteplirsen. This drug is currently in Phase II clinical trials (NCT04004065).^500^ Additionally, PepGen’s PGN-EDO51, designed to skip exon 51 in the DMD gene, has shown promising results in Phase I clinical trials and is now in Phase II (NCT06079736).^501^

Amyotrophic lateral sclerosis (ALS) is a fatal neurodegenerative disease characterized by the dysfunction of upper and lower motor neurons.^502^ Approximately 10% of ALS cases are familial ALS, with various disease-causing genes identified, including superoxide dismutase 1 (SOD1), chromosome 9 open reading frame 72 (C9ORF72), and fused in sarcoma (FUS).^503,504^ The first NAD approved for ALS was tofersen (Qalsody®), a PS 2’-MOE ASO targeting SOD1 mutations.^67,505^ Ulefnersen (ION-363), an antisense therapy targeting the FUS gene, is currently in Phase III trials for FUS-ALS.^506^ However, clinical trials for C9ORF72-targeted therapies WVE-004 and Tadnersen (IONIS-BIIB5Rx) have been terminated due to poor efficacy.

Primary hyperoxaluria type 1 (PH1) is an autosomal recessive disorder caused by defects in hepatic glycoxidation metabolism, leading to excessive endogenous oxalate production.^507^ It is characterized by recurrent urinary calculi, nephrocalcinosis, and progressive renal damage. PH1 is associated with a deficiency or functional defect in alanine glyoxylate aminotransferase.^508,509^ Lumasiran, a siRNA drug that targets hydroxy acid oxidase 1 to reduce hepatic oxalate production, was the first drug approved for PH1.^510^ Nedosiran, another RNAi drug developed by Dicerna Pharmaceuticals that targets LDHA, is currently being evaluated for its efficacy in reducing oxalate production.^330^

SMA is a genetic neuromuscular disease caused by the deletion or mutation of the survival motor neuron 1 (SMN1) gene, leading to the loss of alpha motor neurons and progressive muscle atrophy.^511^ The SMN2 gene, highly similar to SMN1, produces mostly non-functional SMN protein due to differences in exon 7 splicing.^512,513^ Correcting exon 7 splicing in SMN2 can compensate for the lack of SMN1, producing functional SMN protein and improving symptoms.^514,515^ Nusinersen, a modified ASO targeting the intronic splicing silencer N1 of the SMN2 gene, promotes the production of the full-length SMN protein. Several clinical trials have confirmed the efficacy and safety of nusinersen, leading to better motor milestone responses and higher event-free survival rates.^516–518^

### Cancer

The occurrence and progression of cancer are closely tied to the activation of oncogenes and the loss of tumor suppressor genes. NADs, which target specific genes, offer new methods for cancer treatment. For example, transforming growth factor beta 1 (TGFβ1), a multifunctional cytokine, can alter the tumor microenvironment when dysregulated, promoting angiogenesis and immune suppression, thus influencing tumor progression.^482,519^ Therefore, blocking TGF-β signal transduction is a critical strategy in cancer treatment.

Cotsiranib is a siRNA therapy developed by Sirnaomics that uses the PNP delivery platform, containing two active siRNAs targeting TGFβ1 and cytochrome C oxidase subunit 2 mRNA.^482^ A Phase II clinical trial (NCT04669808) for the in situ treatment of basal cell carcinoma showed a positive therapeutic effect and achieved 100% complete clearance. Another anticancer candidate, STP707, is currently being evaluated in a Phase I trial (NCT05037149) for safety, tolerability, and anti-tumor activity in participants with advanced/metastatic or surgically unresectable solid tumors.

In addition, tumor vaccines carrying NADs are considered feasible therapeutic approaches for most solid tumors. IGV-001 is a vaccine targeting GBM, designed based on the Imvax platform Goldspire™. Unlike traditional tumor vaccines, IGV-001 is prepared by mixing tumor tissue removed from the patient’s brain with an insulin-like growth factor 1 receptor antisense oligonucleotide (IMV-001), which inhibits tumor growth. Then, this mixture is encapsulated in biodiffusion chambers with a 0.1-μm pore size, allowing large molecules to permeate and be implanted into the patient’s abdomen.^520^ This aims to induce a tumor-specific immune response. In earlier clinical trials, IGV-001 demonstrated good tolerance and safety (NCT02507583).^520^ Imvax is conducting a randomized, multicenter, double-blind, placebo-controlled Phase IIb study (NCT04485949) to evaluate IGV-001 in patients with newly diagnosed GBM.^521^ Additionally, personalized mRNA neoantigen vaccines, such as mRNA-4157, encode multiple antigens to stimulate a patient-specific immune response. Clinical trials are underway for melanoma, non-small cell lung cancer, and other solid tumors. A Phase IIb trial of mRNA-4157 in combination with pembrolizumab for patients with resected high-risk melanoma showed that this combination therapy prolonged recurrence-free survival compared with pembrolizumab monotherapy and had a manageable safety profile.^522^ Combination therapies for melanoma and non-small cell lung cancer have entered Phase III clinical trials (NCT05933577 and NCT06077760). This is expected to be the first mRNA tumor vaccine on the market.^523^

### Ophthalmic diseases

AMD is a complex eye disease primarily categorized into dry and wet/neovascular AMD.^524^ Aptamers have shown promise in AMD treatment. Pegaptanib, a VEGF antagonist with a three-dimensionally structured aptamer, was initially used to inhibit pathological neovascularization and reverse disease progression but was later removed from the market. RBM-007, an RNA aptamer designed to target fibroblast growth factor 2 (FGF2), is currently in Phase II clinical trials for treating neovascular AMD (nAMD).^178,525^ FGF2 promotes angiogenesis and retinal fibrosis by stimulating vascular endothelial cell proliferation and inducing VEGF secretion. Unlike traditional anti-angiogenic therapies that target VEGF, Sylentis developed SYL1801, an siRNA targeting NOTCH Regulated Ankyrin Repeat Protein (NRARP), to prevent and control nAMD progression. SYL1801 is administered as eye drops rather than via intravitreal injection, downregulating NRARP expression and inhibiting retinal neovascularization.^526^ Clinical trials testing various doses of SYL1801 eye drops in healthy volunteers showed good safety and tolerability. Four of the 36 patients tested experienced mild adverse events such as blepharitis, keratitis, hyperemia, and ocular irritation, all of which resolved within 72 h.^527^ Currently, the drug is in Phase II clinical recruitment to compare the safety and efficacy of three different doses in patients with nAMD (NCT05637255).

Dry eye disease (DED) is a common ocular condition characterized by tear film alterations, ocular inflammation, and neurosensory abnormalities.^528–530^ Tivanisiran (SYL1001), an unmodified siRNA eye drop developed by Sylentis, targets the transient receptor potential vanilloid 1 mRNA to alleviate ocular discomfort and pain.^528^ It has improved the quality of eye redness and tear film in human and animal models. It is undergoing Phase III trials to evaluate its safety in treating DED caused by dry eye syndrome (NCT05310422 and NCT04819269).

QR-110, an ASO drug in Phase III trials, was designed to address Leber congenital amaurosis type 10 caused by the p.Cys998X mutation in the centrosomal protein 290 gene.^531^ QR-110 binds to pre-mRNA, restoring proper splicing and producing fully functional proteins.^532^ Another candidate, ultevursen (QR-421a), is an ASO therapy for treating vision loss in patients with pigmentary retinopathy due to mutations in exon 13 of the USH2A gene. This drug aims to halt or reverse vision loss by restoring the function of the usherin protein through exon skipping in Usher syndrome type 2a and non-syndromic retinitis pigmentosa.^533^

### CVDs

CVDs remain a leading cause of mortality worldwide and pose a severe public health challenge. These diseases, including atherosclerosis, myocardial infarction, cardiac hypertrophy, and heart failure, are closely linked to genetic, metabolic, and environmental factors.^534^ Researchers continually explore new disease targets and develop precision-targeted NADs to address CVDs. Advancements in nucleic acid chemistry have considerably improved drug stability and pharmacokinetics, offering more effective long-term treatment options for reducing the incidence and mortality of CVDs.

Genetic, epidemiological, and clinical research has indicated that lipid abnormalities, such as in low-density lipoprotein cholesterol (LDL-C), are critical factors in the occurrence and progression of CVDs.^535,536^ Proprotein convertase subtilisin/kexin type 9 (PCSK9) is an essential regulator of LDL-C metabolism. By reducing the number of LDL receptors (LDLR) on the surface of liver cells, PCSK9 leads to the accumulation of LDL-C, increasing the risk of CVDs.^537,538^ NADs targeting PCSK9 work by blocking the synthesis of PCSK9 protein and preventing LDLR degradation, thus promoting LDL-C absorption and reducing its levels in the bloodstream. Inclisiran, the world’s first siRNA drug for CVDs, is administered via subcutaneous injection and conjugated with N-acetyl galactosamine to enhance its targeted uptake by liver cells.^539^ Results from several Phase III clinical trials (ORION-9/10/11, NCT03397121, NCT03399370, and NCT03400800) have shown that subcutaneous injections of inclisiran every 6 months considerably reduce LDL-C levels by 48–52%, demonstrating sustained efficacy and good tolerability. Injection site reactions were more common in the inclisiran group but were generally mild and not persistent (5.0% vs. 0.7%).^540,541^ Inclisiran has been approved for treating various cardiovascular conditions, including atherosclerotic cardiovascular disease (ASCVD), primary hypercholesterolemia, mixed dyslipidemia, and heterozygous familial hypercholesterolemia (HeFH).

Lipoprotein(a) [Lp(a)] is a cholesterol-rich LDL-like particle formed by the covalent bonding of apolipoprotein(a) [Apo(a)] and Apo B100 via disulfide bonds. Elevated Lp(a) levels are considered independent risk factors for ASCVD, heart failure, and aortic valve stenosis.^542–544^ Although no targeted drug for reducing Lp(a) levels has been approved thus far, several candidates have shown promise in clinical trials.^545^ For example, pelacarsen, an Lp(a) ASO drug developed by Ionis in collaboration with Novartis AG, effectively lowers Lp(a) levels by selectively cleaving Apo(a) gene (LPA) mRNA. In a Phase II trial, pelacarsen reduced Lp(a) levels by 35–58% depending on the dose, with no significant differences in safety indicators compared with the placebo.^542^ The Phase III trial Lp(a)-HORIZON is underway, involving 8,323 patients with established ASCVD, and is expected to be completed in 2025 (NCT04023552). In addition to ASO drugs, siRNA therapies offer unique solutions for CVDs. Olpasiran and lepodisiran are GalNAc conjugates containing siRNAs that target the LPA gene, promoting RISC-mediated degradation of Apo(a) mRNA and preventing Lp(a) particle assembly in hepatocytes.^546,547^ Olpasiran demonstrated a dose-dependent reduction in Lp(a) levels, with a single dose of 9 mg or higher reducing Lp(a) concentrations by over 90% for several months (NCT03626662).^548^ Phase II trials of olpasiran [OCEAN(a)-DOSE] (NCT04270760) showed that a higher dose (75 or 225 mg) could reduce the Lp(a) level of patients by more than 95% compared with the placebo. Regarding safety, the overall incidence of AEs was similar between the dose and placebo groups. Among them, the most common AE of olpasiran was injection site reaction (primarily pain).^547^ The Phase III trial of olpasiran aims to compare its effects with the placebo on the risk of coronary heart disease death, myocardial infarction, and urgent coronary revascularization in ASCVD patients with elevated Lp(a), with results expected by 2026 (NCT05581303). Similarly, lepodisiran has shown well-tolerated and dose-dependent reductions in serum Lp(a) concentrations, with a Phase III trial currently recruiting participants and expected to be completed by 2029 (NCT06292013 and NCT04914546).^546^

Beyond lipid-lowering therapies, there are positive developments in other areas of NADs application. Zilebesiran (ALN-AGT01), which targets angiotensinogen (AGT), is being developed for hypertension treatment. It suppresses the generation of angiotensin I and II, inhibiting the renin-angiotensin-aldosterone system to reduce blood pressure.^549^ Phase I studies showed that a single subcutaneous dose of zilebesiran of 200 mg or more considerably decreased serum AGT levels and 24 h ambulatory blood pressure for up to 24 weeks, with only mild injection site reactions observed.^550^ In Phase II trials (KARDIA-1), zilebesiran reduced systolic blood pressure in all treated patients, with effects lasting 6 months, opening new possibilities for NADs use in treating hypertension and CVDs.^551^

### Infectious diseases

The COVID-19 pandemic, caused by the severe acute respiratory SARS-CoV-2, has considerably advanced the development of mRNA vaccines. These candidate vaccines are LNP-encapsulated mRNA-based formulations that encode the full-length SARS-CoV-2 spike protein and its receptor-binding domain. They induce an immune response again st the spike protein on the virus’s surface, blocking its entry into cells and providing an antiviral effect.^552,553^ mRNA-1273 was one of the first COVID-19 vaccines to enter clinical trials, taking only 42 days from the publication of the virus’s genetic sequence to the production of the first batch of samples. The final analysis from its Phase III clinical trials showed a 94.1% efficacy in preventing COVID-19 illness and a 100% efficacy in preventing severe COVID-19.^554^ Similarly, BioNTech’s mRNA vaccine BNT162b2 demonstrated a 95% efficacy against COVID-19, with protection rates of over 94% for individuals aged 65 and older.^555^ These vaccines were designed, manufactured, evaluated, and brought to market in an extremely short period compared with traditional vaccine development timelines, highlighting the unique advantages of mRNA vaccines, including high safety, simple design, broad target range, and ease of scaling up production.

Chronic hepatitis B, caused by hepatitis B virus (HBV) infection, is a significant global public health problem. Current clinical treatment guidelines aim for a functional cure for CHB, which involves sustained negativity for hepatitis B surface antigen (HBsAg), undetectable HBV DNA, normal liver biochemical indicators, and improvement of liver tissue lesions after treatment cessation.^556^ Despite the efficacy of existing drugs, achieving a functional cure remains challenging. Bepirovirsen, developed by GlaxoSmithKline (GSK), is a 2’-MOE-modified ASO that targets all HBV RNAs, promoting RNase H-mediated RNA degradation, reducing HBV replication, and inhibiting HBsAg production.^557^ Additionally, bepirovirsen has immunostimulatory activity through Toll-like receptor 8, potentially helping the immune system permanently clear the virus from the bloodstream. Data from a Phase IIb clinical trial indicated that after 24 weeks of treatment, bepirovirsen can reduce levels of HBsAg and HBV DNA.^558^ Furthermore, another Phase II trial (B-Sure) evaluated the durability of bepirovirsen’s antiviral effect (NCT04954859). Recruitment for Phase III clinical trials to assess the efficacy and safety of bepirovirsen is also underway, with results expected in early 2026 (NCT05630807 and NCT05630820).^559^ Several candidate siRNA drugs, including GSK-5637608 (JNJ-73763989, GSK),^560^ VIR-2218 (ALN-HBV02, Alnylam & Brii Biosciences Ltd. & Vir Biotechnology),^561^ and imdusiran (ARB-270729, Arbutus Biopharma),^562^ have entered Phase II clinical trials, demonstrating a strong ability to reduce HBsAg levels. In practical clinical applications, these siRNA therapies often form combination treatments with nucleoside analogs, TLR agonists, or peginterferon, considerably enhancing the possibility of a functional cure for HBV. For example, VIR-2218, a GalNAc-siRNA conjugate that targets HBV, uses enhanced stability chemistry plus technology to stabilize the siRNA in vivo and reduce off-target effects.^561^ Currently, combination therapies involving VIR-2218 are in multiple Phase II trials for HBV patients, and results are eagerly awaited (NCT04856085 and NCT04412863).

## Future perspectives of NADs development

Although many NADs have shown promising preclinical results, the number of NADs approved by the FDA for clinical use remains limited. The complexity of nucleic acids and their types, sizes, and mechanisms of action have made the development of NADs systematically very complex. For successful NADs design, critical factors must be considered regarding sequence design, modification, and delivery, as well as the clinical translation, medical indication, and scale production feasibility.

### Sequence design of NADs

Typically, NADs are designed and screened based on the pathogenic genes of specific diseases. By leveraging existing sequence design and screening techniques, AI-assisted designs have accelerated the design and screening process, making it more accurate, and have been adapted to the needs of personalized therapy.^215,563^ Furthermore, patenting key modification sites in the selected target sequences is crucial to maintaining a competitive advantage.

### Structure modification and delivery system

Considering the inherent physicochemical properties of NADs, the critical issue for their application is whether they can reach the target site and exert the expected therapeutic effects. Much evidence supports that it is unrealistic to address all drug delivery issues through a single modification method or a “universal” delivery vector. NADs should exhibit good stability to withstand nucleases and immune system clearance to achieve sound therapeutic effects. In addition to being delivered explicitly to the required tissues, they must reach the targeted cells, efficiently released into the cytoplasm, and exhibit in vivo biocompatibility. Initially, choosing appropriate strategies for the design of NADs is necessary based on the disease type and the required functional nucleic acid. For example, from the approved NADs, ASO drugs often do not require carriers for efficient delivery and only require appropriate modifications, such as PS and 2’ position modifications. Small NADs, such as siRNA, and large ones, such as mRNA, in addition to the necessary modifications, still require the aid of delivery vectors. siRNA typically uses nucleic acid conjugations for delivery, while mRNAs often rely on LNPs for compression and encapsulation. Furthermore, understanding the structure-activity relationship between nucleic acids and the delivery vector and different interactions (hydrophobic, electrostatic, and covalent interactions) and their effect on drug stability is crucial for developing stable and effective NADs. NADs interact with various environments during delivery, and individual chemical modifications or delivery carriers may not suffice to overcome physiological barriers. Combining nucleic acid chemical modifications with drug delivery systems is promising for better therapeutic outcomes. Moreover, researchers are developing various combined carrier-use modes to facilitate effective drug loading, precise targeting, and release. The emergence of intelligent, responsive nanocarriers may help NADs interact with complex environmental changes, responding to specific in vivo changes, such as pH, redox conditions, or external stimuli (ultrasound, light, magnetic fields, and electric fields). These carriers can improve transfection efficiency in target cells and reduce toxic side effects on normal tissues and cells.^564,565^ However, hybridizing multiple carriers and modifying functional ligand molecules increase the technical difficulties and manufacturing costs.

### Clinical application

#### Safety

A serious issue in developing these therapeutics is in vivo safety. Despite the detailed understanding of the physicochemical properties of NADs, the potential immunogenicity and toxicity of carriers and related modifications may pose additional challenges to safe and effective NADs delivery.^500,566^ For example, early application of relatively mature PPMO technology showed that high doses of CPP-PMO conjugates caused kidney injury in rats.^567^ Cationic polymers, such as PLL/PEI,^305,568^ have induced cell apoptosis and inflammation in vivo.

#### Pharmacokinetic properties and adverse reactions

Advanced technologies have generated NADs complexes with different properties.^569^ Their chemical structure, dosage form, and administration route are the main determinants of their absorption, distribution, metabolism, and excretion, thereby affecting their efficacy and safety.^570,571^ For example, based on the unique mechanism of action of ASOs, the main safety issues are severe hepatotoxicity and nephrotoxicity caused by drug accumulation.^64^ Therefore, evaluating NADs delivery systems’ drug metabolism and pharmacokinetics is essential for clinical development. Various bioanalytical methods, such as chromatographic techniques for determining drug plasma concentrations and metabolism^569,572^ and imaging technology for estimating drug distribution,^573^ are used, although each has limitations. Low sensitivity and poor discrimination affect the accuracy of determination results,^574^ highlighting the importance of developing novel analytical methods. Although previous studies have used nano-fluorescent probes to detect the cellular delivery efficiency of oligonucleotide molecules,^403,457,575^ more pharmacokinetic data on biodistribution, immune compatibility, and toxicity are needed for clinical translation and practical therapeutic application of these systems.^266,576^

### Pathological indications

Most diseases exhibit variability in their phenotypes, making it difficult to achieve a curative effect by targeting a single gene. Issues, such as a small patient population and unique targets, often result in a lack of attention from pharmaceutical companies. Therefore, analyzing the pathogenesis of diseases at the genetic level and developing a personalized treatment plan may offer the possibility of a permanent cure for rare and currently incurable genetic diseases. Compared with antibody drugs and small molecule drugs, NADs are specifically favorable for personalized therapies owing to their strong specificity for diverse targets. They have been explored in the development of new therapies for various diseases. Thus far, genetic diseases have been the most approved indication category for NADs. Additionally, breakthroughs in gene therapy and gene-cell combined therapy are promising for the large-scale clinical application of NADs. For instance, Casgevy®, based on the CRISPR/Cas9 gene-editing system, was the first successful attempt at disease treatment, promising a “one-time treatment for a lifelong effect.” However, the complexity of clinical applications and the difficulty of target selection in gene editing have led to long treatment cycles and high costs. Moreover, the application range of NADs is gradually expanding from rare diseases to common diseases such as chronic diseases, infectious diseases, and ophthalmic diseases. The development of curative drugs for indications with large patient populations, such as potential cures for hepatitis B, may address broader clinical needs.

### Scale production

Like other drugs, the production of NADs involves multiple technical stages, including raw material collection, synthesis, separation, purification, transportation, and storage, requiring large-scale production capabilities and stringent quality control.^577^ Additionally, the specific requirements for sequence size, purity, and modification and delivery methods of NADs, based on different research needs, increase the demands on the production model.^214^ Therefore, to achieve large-scale clinical application, NADs must ensure easy production, quality control, and transportation. Moreover, as regulatory policies become more refined and the industry chain further develops, companies should seek collaboration opportunities while increasing research and development investments. Collaborations between internationally renowned enterprises in terms of sharing advanced technology and management experience can enhance research and development capabilities and production capacity.

In summary, the development of NADs has undergone a long process, with research on the mechanisms of action of different types of drugs and breakthroughs in chemical modification and delivery technologies. These have transformed NADs from conceptual to practical clinical treatment tools. However, some clinical promotion and application shortcomings still require further development. The organic combination of specific therapeutic nucleic acid molecules, targeted modifications, and functionalized delivery carriers may become vital to achieving personalized nucleic acid therapy and addressing unmet clinical needs.

## References

[1] Zhang, S. et al. The mechanistic, diagnostic and therapeutic novel nucleic acids for hepatocellular carcinoma emerging in past score years. *Brief. Bioinform.* **22**, 1860–1883 (2021). 32249290 10.1093/bib/bbaa023

[2] Smith, C. I. E. & Zain, R. Therapeutic oligonucleotides: state of the art. *Annu. Rev. Pharmacol. Toxicol.* **59**, 605–630 (2019). 30285540 10.1146/annurev-pharmtox-010818-021050

[3] Friedmann, T. & Roblin, R. Gene therapy for human genetic disease? *Science* **175**, 949–955 (1972). 5061866 10.1126/science.175.4025.949

[4] Vaughan, H. J., Green, J. J. & Tzeng, S. Y. Cancer-targeting nanoparticles for combinatorial nucleic acid delivery. *Adv. Mater.* **32**, e1901081 (2020). 31222852 10.1002/adma.201901081 PMC6923623

[5] Garbo, S., Maione, R., Tripodi, M. & Battistelli, C. Next RNA therapeutics: the mine of non-coding. *Int. J. Mol. Sci.* **23**, 7471 (2022). 35806476 10.3390/ijms23137471 PMC9267739

[6] Meng, F., Wang, J. & Yeo, Y. Nucleic acid and oligonucleotide delivery for activating innate immunity in cancer immunotherapy. *J. Control. Release* **345**, 586–600 (2022). 35351528 10.1016/j.jconrel.2022.03.045 PMC9133138

[7] To, K. K. W. & Cho, W. C. S. An overview of rational design of mRNA-based therapeutics and vaccines. *Expert Opin. Drug Discov.* **16**, 1307–1317 (2021). 34058918 10.1080/17460441.2021.1935859

[8] Del Pozo-Rodriguez, A. et al. Gene therapy. *Adv. Biochem. Eng. Biotechnol.* **171**, 321–368 (2020). 31492963 10.1007/10_2019_109

[9] Kumari, N. et al. Oral delivery of nucleic acid therapies for local and systemic action. *Pharm. Res.* **40**, 107–122 (2023). 36271204 10.1007/s11095-022-03415-7 PMC9589866

[10] Maishi, N. et al. Novel antiangiogenic therapy targeting biglycan using tumor endothelial cell-specific liposomal siRNA delivery system. *Cancer Sci.* **113**, 1855–1867 (2022). 35266253 10.1111/cas.15323 PMC9128192

[11] Gan, L. et al. A cell-penetrating peptide enhances delivery and efficacy of phosphorodiamidate morpholino oligomers in mdx mice. *Mol. Ther. Nucl. Acids* **30**, 17–27 (2022). 10.1016/j.omtn.2022.08.019 PMC9483789 36189424

[12] Yan, X. et al. Redox-responsive multifunctional polypeptides conjugated with Au nanoparticles for tumor-targeting gene therapy and their 1 + 1 > 2 synergistic effects. *ACS Biomater. Sci. Eng.* **6**, 463–473 (2020). 33463244 10.1021/acsbiomaterials.9b01581

[13] Lehto, T., Ezzat, K., Wood, M. J. A. & El Andaloussi, S. Peptides for nucleic acid delivery. *Adv. Drug Deliv. Rev.* **106**, 172–182 (2016). 27349594 10.1016/j.addr.2016.06.008

[14] Gokirmak, T. et al. Overcoming the challenges of tissue delivery for oligonucleotide therapeutics. *Trends Pharmacol. Sci.* **42**, 588–604 (2021). 34020790 10.1016/j.tips.2021.04.010

[15] Tan, X., Jia, F., Wang, P. & Zhang, K. Nucleic acid-based drug delivery strategies. *J. Control. Release* **323**, 240–252 (2020). 32272123 10.1016/j.jconrel.2020.03.040 PMC8079167

[16] Sousa De Almeida, M., Rothen-Rutishauser, B., Mayer, M. & Taskova, M. Multi-functionalized heteroduplex antisense oligonucleotides for targeted intracellular delivery and gene silencing in hela cells. *Biomedicines* **10**, 2096 (2022). 36140196 10.3390/biomedicines10092096 PMC9495875

[17] Zhao, Y., Shu, R. & Liu, J. The development and improvement of ribonucleic acid therapy strategies. *Mol. Ther. Nucl. Acids* **26**, 997–1013 (2021). 10.1016/j.omtn.2021.09.002 PMC8437697 34540356

[18] Sarli, S. L. & Watts, J. K. Harnessing nucleic acid technologies for human health on earth and in space. *Life Sci. Space Res.* **35**, 113–126 (2022). 10.1016/j.lssr.2022.08.006 PMC11845088 36336357

[19] Scully, M. A., Sterin, E. H. & Day, E. S. Membrane-wrapped nanoparticles for nucleic acid delivery. *Biomater. Sci.* **10**, 4378–4391 (2022). 35796319 10.1039/d2bm00447j PMC9486671

[20] Van Den Berg, A. I. S., Yun, C. O., Schiffelers, R. M. & Hennink, W. E. Polymeric delivery systems for nucleic acid therapeutics: approaching the clinic. *J. Control. Release* **331**, 121–141 (2021). 33453339 10.1016/j.jconrel.2021.01.014

[21] Panigaj, M. et al. Therapeutic immunomodulation by rationally designed nucleic acids and nucleic acid nanoparticles. *Front. Immunol.* **14**, 1053550 (2023). 36798121 10.3389/fimmu.2023.1053550 PMC9927404

[22] Hager, S. & Wagner, E. Bioresponsive polyplexes-chemically programmed for nucleic acid delivery. *Expert Opin. Drug Deliv.* **15**, 1067–1083 (2018). 30247975 10.1080/17425247.2018.1526922

[23] Zhang, C. et al. Modification of lipid-based nanoparticles: an efficient delivery system for nucleic acid-based immunotherapy. *Molecules* **27**, 1943 (2022). 35335310 10.3390/molecules27061943 PMC8949521

[24] Steffens, R. C. & Wagner, E. Directing the way-receptor and chemical targeting strategies for nucleic acid delivery. *Pharm. Res.* **40**, 47–76 (2023). 36109461 10.1007/s11095-022-03385-w PMC9483255

[25] Evers, M. J. W. et al. Functional siRNA delivery by extracellular vesicle-liposome hybrid nanoparticles. *Adv. Healthc. Mater.* **11**, e2101202 (2022). 34382360 10.1002/adhm.202101202 PMC11468224

[26] Zhao, J. et al. Polyester-based nanoparticles for nucleic acid delivery. *Biomater. Adv.* **92**, 983–994 (2018). 10.1016/j.msec.2018.07.027 30184828

[27] Wang, L. et al. Improved transfer efficiency of supercharged 36 + GFP protein mediate nucleic acid delivery. *Drug Deliv.* **29**, 386–398 (2022). 35075948 10.1080/10717544.2022.2030430 PMC8794074

[28] Thess, A. et al. Historic nucleic acids isolated by Friedrich Miescher contain RNA besides DNA. *Biol. Chem.* **402**, 1179–1185 (2021). 34523295 10.1515/hsz-2021-0226

[29] Varshavsky, A. Discovering the RNA double helix and hybridization. *Cell* **127**, 1295–1297 (2006). 17190591 10.1016/j.cell.2006.12.008

[30] Rich, A. & Davies, D. R. A new two stranded helical structure: polyadenylic acid and polyuridylic acid. *J. Am. Chem. Soc.* **78**, 3548–3549 (1956).

[31] Rich, A. A hybrid helix containing both deoxyribose and ribose polynucleotides and its relation to the transfer of information between the nucleic acids. *Proc. Natl Acad. Sci. USA* **46**, 1044–1053 (1960). 16590711 10.1073/pnas.46.8.1044 PMC222998

[32] Zamecnik, P. C. & Stephenson, M. L. Inhibition of rous sarcoma virus replication and cell transformation by a specific oligodeoxynucleotide. *Proc. Natl Acad. Sci. USA* **75**, 280–284 (1978). 75545 10.1073/pnas.75.1.280 PMC411230

[33] Berget, S. M., Moore, C. & Sharp, P. A. Spliced segments at the 5’ terminus of adenovirus 2 late mRNA. *Proc. Natl Acad. Sci. USA* **74**, 3171–3175 (1977). 269380 10.1073/pnas.74.8.3171 PMC431482

[34] Suran, M. Finding the tail end: the discovery of RNA splicing. *Proc. Natl Acad. Sci. USA* **117**, 1829–1832 (2020). 31871165 10.1073/pnas.1919416116 PMC6994983

[35] Li, Y. I. et al. RNA splicing is a primary link between genetic variation and disease. *Science* **352**, 600–604 (2016). 27126046 10.1126/science.aad9417 PMC5182069

[36] Tian, J. et al. Aberrant RNA splicing is a primary link between genetic variation and pancreatic cancer risk. *Cancer Res* **82**, 2084–2096 (2022). 35363263 10.1158/0008-5472.CAN-21-4367

[37] Dominski, Z. & Kole, R. Restoration of correct splicing in thalassemic pre-mRNA by antisense oligonucleotides. *Proc. Natl Acad. Sci. USA* **90**, 8673–8677 (1993). 8378346 10.1073/pnas.90.18.8673 PMC47420

[38] Fire, A. et al. Potent and specific genetic interference by double-stranded RNA in caenorhabditis elegans. *Nature* **391**, 806–811 (1998). 9486653 10.1038/35888

[39] Mccaffrey, A. P. et al. RNA interference in adult mice. *Nature* **418**, 38–39 (2002). 12097900 10.1038/418038a

[40] Zamore, P. D. RNA interference: big applause for silencing in stockholm. *Cell* **127**, 1083–1086 (2006). 17174883 10.1016/j.cell.2006.12.001

[41] Davis, M. E. et al. Evidence of RNAi in humans from systemically administered siRNA via targeted nanoparticles. *Nature* **464**, 1067–1070 (2010). 20305636 10.1038/nature08956 PMC2855406

[42] Maraganore, J. Reflections on Alnylam. *Nat. Biotechnol.* **40**, 641–650 (2022). 35534556 10.1038/s41587-022-01304-3

[43] Baltimore, D. RNA-dependent DNA polymerase in virions of RNA tumour viruses. *Nature* **226**, 1209–1211 (1970). 4316300 10.1038/2261209a0

[44] Reich, E., Franklin, R. M., Shatkin, A. J. & Tatumel Action of actinomycin D on animal cells and viruses. *Proc. Natl Acad. Sci. USA* **48**, 1238–1245 (1962). 14491128 10.1073/pnas.48.7.1238 PMC220938

[45] Krieg, P. A. & Melton, D. A. Functional messenger RNAs are produced by SP6 in vitro transcription of cloned cDNAs. *Nucleic Acids Res* **12**, 7057 (1984). 6207484 10.1093/nar/12.18.7057 PMC320142

[46] Wolff, J. A. et al. Direct gene transfer into mouse muscle in vivo. *Science* **247**, 1465–1468 (1990). 1690918 10.1126/science.1690918

[47] Jirikowski, G. F., Sanna, P. P., Maciejewski-Lenoir, D. & Bloom, F. E. Reversal of diabetes insipidus in brattleboro rats: intrahypothalamic injection of vasopressin mRNA. *Science* **255**, 996–998 (1992). 1546298 10.1126/science.1546298

[48] Conry, R. M. et al. Characterization of a messenger RNA polynucleotide vaccine vector. *Cancer Res* **55**, 1397–1400 (1995). 7882341

[49] Kariko, K., Buckstein, M., Ni, H. & Weissman, D. Suppression of RNA recognition by Toll-like receptors: the impact of nucleoside modification and the evolutionary origin of RNA. *Immunity* **23**, 165–175 (2005). 16111635 10.1016/j.immuni.2005.06.008

[50] Jackson, L. A. et al. An mRNA vaccine against SARS-CoV-2 - preliminary report. *N. Engl. J. Med.* **383**, 1920–1931 (2020). 32663912 10.1056/NEJMoa2022483 PMC7377258

[51] Sahin, U. et al. COVID-19 vaccine BNT162b1 elicits human antibody and T(H)1 T cell responses. *Nature* **586**, 594–599 (2020). 32998157 10.1038/s41586-020-2814-7

[52] Rossi, J. J. & Rossi, D. Oligonucleotides and the COVID-19 pandemic: a perspective. *Nucleic Acid Ther.* **30**, 129–132 (2020). 32297843 10.1089/nat.2020.0868 PMC7249453

[53] Dolgin, E. The tangled history of mRNA vaccines. *Nature* **597**, 318–324 (2021). 34522017 10.1038/d41586-021-02483-w

[54] Kariko, K. et al. Incorporation of pseudouridine into mRNA yields superior nonimmunogenic vector with increased translational capacity and biological stability. *Mol. Ther.* **16**, 1833–1840 (2008). 18797453 10.1038/mt.2008.200 PMC2775451

[55] Gostimskaya, I. CRISPR-Cas9: a history of its discovery and ethical considerations of its use in genome editing. *Biochemistry* Mosc **87**, 777–788 (2022). 10.1134/S0006297922080090 PMC9377665 36171658

[56] Zhang, H. et al. Application of the CRISPR/Cas9-based gene editing technique in basic research, diagnosis, and therapy of cancer. *Mol. Cancer* **20**, 126 (2021). 34598686 10.1186/s12943-021-01431-6 PMC8484294

[57] Boti, M. A. et al. Recent advances in genome-engineering strategies. *Genes* **14**, 129 (2023). 36672870 10.3390/genes14010129 PMC9859587

[58] Frangoul, H., Ho, T. W. & Corbacioglu, S. CRISPR-Cas9 gene editing for sickle cell disease and β-thalassemia. Reply. *N. Engl. J. Med.* **384**, e91 (2021). 34107197 10.1056/NEJMc2103481

[59] Lu, Z. G. et al. Nucleic acid drug vectors for diagnosis and treatment of brain diseases. *Signal Transduct. Target Ther.* **8**, 604–656 (2023). 10.1038/s41392-022-01298-z PMC9844208 36650130

[60] Matsui, M. & Corey, D. R. Non-coding RNAs as drug targets. *Nat. Rev. Drug Discov.* **16**, 167–179 (2017). 27444227 10.1038/nrd.2016.117 PMC5831170

[61] Bennett, C. F. & Swayze, E. E. RNA targeting therapeutics: molecular mechanisms of antisense oligonucleotides as a therapeutic platform. *Annu. Rev. Pharmacol. Toxicol.* **50**, 259–293 (2010). 20055705 10.1146/annurev.pharmtox.010909.105654

[62] Dhuri, K. et al. Antisense oligonucleotides: an emerging area in drug discovery and development. *J. Clin. Med.* **9**, 2004 (2020). 32604776 10.3390/jcm9062004 PMC7355792

[63] Stein, C. A. & Castanotto, D. FDA-approved oligonucleotide therapies in 2017. *Mol. Ther.* **25**, 1069–1075 (2017). 28366767 10.1016/j.ymthe.2017.03.023 PMC5417833

[64] Alhamadani, F. et al. Adverse drug reactions and toxicity of the food and drug administration-approved antisense oligonucleotide drugs. *Drug Metab. Dispos.* **50**, 879–887 (2022). 35221289 10.1124/dmd.121.000418 PMC11022857

[65] Gales, L. Tegsedi (Inotersen): an antisense oligonucleotide approved for the treatment of adult patients with hereditary transthyretin amyloidosis. *Pharm. (Basel)* **12**, 78 (2019). 10.3390/ph12020078 PMC6631675 31117178

[66] Paik, J. & Duggan, S. Volanesorsen: first global approval. *Drugs* **79**, 1349–1354 (2019). 31301033 10.1007/s40265-019-01168-z

[67] Blair, H. A. Tofersen: first approval. *Drugs* **83**, 1039–1043 (2023). 37316681 10.1007/s40265-023-01904-6

[68] Adewunmi, O., Shen, Y., Zhang, X. H. & Rosen, J. M. Targeted inhibition of lncRNA malat1 alters the tumor immune microenvironment in preclinical syngeneic mouse models of triple-negative breast cancer. *Cancer Immunol. Res.* **11**, 1462–1479 (2023). 37603945 10.1158/2326-6066.CIR-23-0045 PMC10618655

[69] Amodio, N. et al. Drugging the lncRNA MALAT1 via LNA gapmeR ASO inhibits gene expression of proteasome subunits and triggers anti-multiple myeloma activity. *Leukemia* **32**, 1948–1957 (2018). 29487387 10.1038/s41375-018-0067-3 PMC6127082

[70] Esposito, R. et al. Multi-hallmark long noncoding RNA maps reveal non-small cell lung cancer vulnerabilities. *Cell Genomics* **2**, 100171 (2022). 36778670 10.1016/j.xgen.2022.100171 PMC9903773

[71] Chen, Y., Li, Z., Chen, X. & Zhang, S. Long non-coding RNAs: from disease code to drug role. *Acta Pharm. Sin. B* **11**, 340–354 (2021). 33643816 10.1016/j.apsb.2020.10.001 PMC7893121

[72] De Santi, C. et al. Precise targeting of miRNA sites restores CFTR activity in CF bronchial epithelial cells. *Mol. Ther.* **28**, 1190–1199 (2020). 32059764 10.1016/j.ymthe.2020.02.001 PMC7132615

[73] Sun, Q. et al. Expression and significance of miRNA-21 and BTG2 in lung cancer. *Tumour Biol.* **34**, 4017–4026 (2013). 23857284 10.1007/s13277-013-0992-8

[74] Wang, P. Y. et al. Regulating A549 cells growth by ASO inhibiting miRNA expression. *Mol. Cell. Biochem.* **339**, 163–171 (2010). 20049626 10.1007/s11010-009-0380-2

[75] Havens, M. A. & Hastings, M. L. Splice-switching antisense oligonucleotides as therapeutic drugs. *Nucleic Acids Res* **44**, 6549–6563 (2016). 27288447 10.1093/nar/gkw533 PMC5001604

[76] Aung-Htut, M. T. et al. Systematic approach to developing splice modulating antisense oligonucleotides. *Int. J. Mol. Sci.* **20**, 5030 (2019). 31614438 10.3390/ijms20205030 PMC6834167

[77] Mogilevsky, M. et al. Modulation of MKNK2 alternative splicing by splice-switching oligonucleotides as a novel approach for glioblastoma treatment. *Nucleic Acids Res* **46**, 11396–11404 (2018). 30329087 10.1093/nar/gky921 PMC6265459

[78] Li, D. et al. Neurodegenerative diseases: a hotbed for splicing defects and the potential therapies. *Transl. Neurodegener.* **10**, 16 (2021). 34016162 10.1186/s40035-021-00240-7 PMC8136212

[79] Balachandran, A. A., Raguraman, P., Rahimizadeh, K. & Veedu, R. N. Splice-switching antisense oligonucleotides targeting extra- and intracellular domains of epidermal growth factor receptor in cancer cells. *Biomedicines* **11**, 3299 (2023). 38137520 10.3390/biomedicines11123299 PMC10741442

[80] Heo, Y. A. Golodirsen: first approval. *Drugs* **80**, 329–333 (2020). 32026421 10.1007/s40265-020-01267-2

[81] Boiziau, C. et al. Inhibition of translation initiation by antisense oligonucleotides via an RNase-H independent mechanism. *Nucleic Acids Res* **19**, 1113–1119 (1991). 1850511 10.1093/nar/19.5.1113 PMC333789

[82] Dong, Y., Siegwart, D. J. & Anderson, D. G. Strategies, design, and chemistry in siRNA delivery systems. *Adv. Drug Deliv. Rev.* **144**, 133–147 (2019). 31102606 10.1016/j.addr.2019.05.004 PMC6745264

[83] Hu, B. et al. Therapeutic siRNA: state of the art. *Signal Transduct. Target Ther.* **5**, 101 (2020). 32561705 10.1038/s41392-020-0207-x PMC7305320

[84] Wittrup, A. & Lieberman, J. Knocking down disease: a progress report on siRNA therapeutics. *Nat. Rev. Genet.* **16**, 543–552 (2015). 26281785 10.1038/nrg3978 PMC4756474

[85] Elbashir, S. M. et al. Duplexes of 21-nucleotide RNAs mediate RNA interference in cultured mammalian cells. *Nature* **411**, 494–498 (2001). 11373684 10.1038/35078107

[86] Piatek, M. J. & Werner, A. Endogenous siRNAs: regulators of internal affairs. *Biochem. Soc. Trans.* **42**, 1174–1179 (2014). 25110021 10.1042/BST20140068 PMC4289821

[87] Nikam, R. R. & Gore, K. R. Journey of siRNA: clinical developments and targeted delivery. *Nucleic Acid Ther.* **28**, 209–224 (2018). 29584585 10.1089/nat.2017.0715

[88] Rossi, J. J. & Rossi, D. J. siRNA drugs: here to stay. *Mol. Ther.* **29**, 431–432 (2021). 33472033 10.1016/j.ymthe.2021.01.015 PMC7854346

[89] Robb, G. B. & Rana, T. M. RNA helicase A interacts with RISC in human cells and functions in RISC loading. *Mol. Cell* **26**, 523–537 (2007). 17531811 10.1016/j.molcel.2007.04.016

[90] Sarisozen, C., Salzano, G. & Torchilin, V. P. Recent advances in siRNA delivery. *Biomol. Concepts* **6**, 321–341 (2015). 26609865 10.1515/bmc-2015-0019

[91] Isazadeh, H. et al. Advances in siRNA delivery approaches in cancer therapy: challenges and opportunities. *Mol. Biol. Rep.* **50**, 9529–9543 (2023). 37741808 10.1007/s11033-023-08749-y

[92] Kurakula, H., Vaishnavi, S., Sharif, M. Y. & Ellipilli, S. Emergence of small interfering RNA-based gene drugs for various diseases. *ACS Omega* **8**, 20234–20250 (2023). 37323391 10.1021/acsomega.3c01703 PMC10268023

[93] Joshi, B. H. & Pachchigar, K. P. siRNA: novel therapeutics from functional genomics. *Biotechnol. Genet. Eng. Rev.* **30**, 1–30 (2014). 25023460 10.1080/02648725.2014.921495

[94] Ranasinghe, P., Addison, M. L., Dear, J. W. & Webb, D. J. Small interfering RNA: discovery, pharmacology and clinical development-an introductory review. *Br. J. Pharmacol.* **180**, 2697–2720 (2023). 36250252 10.1111/bph.15972

[95] Sajid, M. I. et al. Overcoming barriers for siRNA therapeutics: from bench to bedside. *Pharm. (Basel)* **13**, 294 (2020). 10.3390/ph13100294 PMC7600125 33036435

[96] Gatta, A. K. et al. Strategies for improving the specificity of siRNAs for enhanced therapeutic potential. *Expert Opin. Drug Discov.* **13**, 709–725 (2018). 29902093 10.1080/17460441.2018.1480607

[97] Hoy, S. M. Patisiran: first global approval. *Drugs* **78**, 1625–1631 (2018). 30251172 10.1007/s40265-018-0983-6

[98] Lee, R. C., Feinbaum, R. L. & Ambros, V. TheC. elegans heterochronic gene lin-4 encodes small RNAs with antisense complementarity to lin-14. *Cell* **75**, 843–854 (1993). 8252621 10.1016/0092-8674(93)90529-y

[99] Bhatnagar, D., Ladhe, S. & Kumar, D. Discerning the prospects of miRNAs as a multi-target therapeutic and diagnostic for alzheimer’s disease. *Mol. Neurobiol.* **60**, 5954–5974 (2023). 37386272 10.1007/s12035-023-03446-0

[100] Gregory, R. I. et al. The microprocessor complex mediates the genesis of microRNAs. *Nature* **432**, 235–240 (2004). 15531877 10.1038/nature03120

[101] Han, J. et al. Molecular basis for the recognition of primary microRNAs by the Drosha-DGCR8 complex. *Cell* **125**, 887–901 (2006). 16751099 10.1016/j.cell.2006.03.043

[102] Clancy, J. W., Zhang, Y., Sheehan, C. & D’souza-Schorey, C. An ARF6-Exportin-5 axis delivers pre-miRNA cargo to tumour microvesicles. *Nat. Cell Biol.* **21**, 856–866 (2019). 31235936 10.1038/s41556-019-0345-y PMC6697424

[103] Wang, J. et al. XPO5 promotes primary miRNA processing independently of RanGTP. *Nat. Commun.* **11**, 1845 (2020). 32296071 10.1038/s41467-020-15598-x PMC7160132

[104] Li, Y. et al. The ubiquitin-specific protease USP36 associates with the microprocessor complex and regulates miRNA biogenesis by SUMOylating DGCR8. *Cancer Res. Commun.* **3**, 459–470 (2023). 36950067 10.1158/2767-9764.CRC-22-0344 PMC10026737

[105] Wilson, R. C. et al. Dicer-TRBP complex formation ensures accurate mammalian microRNA biogenesis. *Mol. Cell* **57**, 397–407 (2015). 25557550 10.1016/j.molcel.2014.11.030 PMC4320653

[106] Jungers, C. F. & Djuranovic, S. Modulation of miRISC-mediated gene silencing in eukaryotes. *Front. Mol. Biosci.* **9**, 832916 (2022). 35237661 10.3389/fmolb.2022.832916 PMC8882679

[107] Song, X., Li, Y., Cao, X. & Qi, Y. MicroRNAs and their regulatory roles in plant-environment interactions. *Annu. Rev. Plant Biol.* **70**, 489–525 (2019). 30848930 10.1146/annurev-arplant-050718-100334

[108] Gebauer, F., Schwarzl, T., Valcarcel, J. & Hentze, M. W. RNA-binding proteins in human genetic disease. *Nat. Rev. Genet.* **22**, 185–198 (2021). 33235359 10.1038/s41576-020-00302-y

[109] Gebert, L. F. R. & Macrae, I. J. Regulation of microRNA function in animals. *Nat. Rev. Mol. Cell Biol.* **20**, 21–37 (2019). 30108335 10.1038/s41580-018-0045-7 PMC6546304

[110] Naeli, P. et al. The intricate balance between microRNA-induced mRNA decay and translational repression. *FEBS J.* **290**, 2508–2524 (2023). 35247033 10.1111/febs.16422

[111] Dalmay, T. Mechanism of miRNA-mediated repression of mRNA translation. *Essays Biochem* **54**, 29–38 (2013). 23829525 10.1042/bse0540029

[112] Jame-Chenarboo, F., Ng, H. H., Macdonald, D. & Mahal, L. K. High-throughput analysis reveals miRNA upregulating alpha-2,6-sialic acid through direct miRNA-mRNA interactions. *ACS Cent. Sci.* **8**, 1527–1536 (2022). 36439307 10.1021/acscentsci.2c00748 PMC9686205

[113] Laitinen, P. et al. Nuclear microRNA-466c regulates Vegfa expression in response to hypoxia. *PLoS One* **17**, e0265948 (2022). 35358280 10.1371/journal.pone.0265948 PMC8975276

[114] Chipman, L. B. & Pasquinelli, A. E. miRNA targeting: growing beyond the seed. *Trends Genet* **35**, 215–222 (2019). 30638669 10.1016/j.tig.2018.12.005 PMC7083087

[115] Bonneau, E. et al. How close are miRNAs from clinical practice? A perspective on the diagnostic and therapeutic market. *EJIFCC* **30**, 114–127 (2019). 31263388 PMC6599191

[116] Schmidt, M. F. miRNA targeting drugs: the next blockbusters? *Methods Mol. Biol.* **1517**, 3–22 (2017). 27924471 10.1007/978-1-4939-6563-2_1

[117] Damase, T. R. et al. The limitless future of RNA therapeutics. *Front. Bioeng. Biotech.* **9**, 628137 (2021). 10.3389/fbioe.2021.628137 PMC8012680 33816449

[118] Kim, T. & Croce, C. M. MicroRNA: trends in clinical trials of cancer diagnosis and therapy strategies. *Exp. Mol. Med.* **55**, 1314–1321 (2023). 37430087 10.1038/s12276-023-01050-9 PMC10394030

[119] Chakraborty, C., Sharma, A. R., Sharma, G. & Lee, S. S. Therapeutic advances of miRNAs: a preclinical and clinical update. *J. Adv. Res.* **28**, 127–138 (2021). 33364050 10.1016/j.jare.2020.08.012 PMC7753224

[120] Daige, C. L. et al. Systemic delivery of a miR34a mimic as a potential therapeutic for liver cancer. *Mol. Cancer Ther.* **13**, 2352–2360 (2014). 25053820 10.1158/1535-7163.MCT-14-0209

[121] Gambari, R., Brognara, E., Spandidos, D. A. & Fabbri, E. Targeting oncomiRNAs and mimicking tumor suppressor miRNAs: new trends in the development of miRNA therapeutic strategies in oncology (Review). *Int. J. Oncol.* **49**, 5–32 (2016). 27175518 10.3892/ijo.2016.3503 PMC4902075

[122] Thomson, D. W., Bracken, C. P., Szubert, J. M. & Goodall, G. J. On measuring miRNAs after transient transfection of mimics or antisense inhibitors. *PLoS One* **8**, e55214 (2013). 23358900 10.1371/journal.pone.0055214 PMC3554668

[123] Holjencin, C. & Jakymiw, A. MicroRNAs and their big therapeutic impacts: delivery strategies for cancer intervention. *Cells* **11**, 2332 (2022). 35954176 10.3390/cells11152332 PMC9367537

[124] Gallant-Behm, C. L. et al. A microRNA-29 mimic (Remlarsen) represses extracellular matrix expression and fibroplasia in the skin. *J. Investig. Dermatol.* **139**, 1073–1081 (2019). 30472058 10.1016/j.jid.2018.11.007

[125] Gallant-Behm, C. L. et al. A synthetic microRNA-92a inhibitor (MRG-110) accelerates angiogenesis and wound healing in diabetic and nondiabetic wounds. *Wound Repair Regen.* **26**, 311–323 (2018). 30118158 10.1111/wrr.12660

[126] Seto, A. G. et al. Cobomarsen, an oligonucleotide inhibitor of miR-155, co-ordinately regulates multiple survival pathways to reduce cellular proliferation and survival in cutaneous T-cell lymphoma. *Br. J. Haematol.* **183**, 428–444 (2018). 30125933 10.1111/bjh.15547

[127] Ghosh, S. et al. The microRNA-10b targeted therapeutic, TTX-MC138, is effective in preclinical pancreatic adenocarcinoma. *Cancer Res.* **83**, 548 (2023).

[128] Wang, X. et al. Induction of NANOG expression by targeting promoter sequence with small activating RNA antagonizes retinoic acid-induced differentiation. *Biochem. J.* **443**, 821–828 (2012). 22339500 10.1042/BJ20111491 PMC3327998

[129] Wang, C. et al. Targeted p53 activation by saRNA suppresses human bladder cancer cells growth and metastasis. *J. Exp. Clin. Cancer Res.* **35**, 53 (2016). 27012825 10.1186/s13046-016-0329-8 PMC4807596

[130] Portnoy, V. et al. saRNA-guided Ago2 targets the RITA complex to promoters to stimulate transcription. *Cell Res* **26**, 320–335 (2016). 26902284 10.1038/cr.2016.22 PMC4783471

[131] Gregory, G. L. & Copple, I. M. Modulating the expression of tumor suppressor genes using activating oligonucleotide technologies as a therapeutic approach in cancer. *Mol. Ther. Nucl. Acids* **31**, 211–223 (2023). 10.1016/j.omtn.2022.12.016 PMC9840112 36700046

[132] Janowski, B. A. et al. Activating gene expression in mammalian cells with promoter-targeted duplex RNAs. *Nat. Chem. Biol.* **3**, 166–173 (2007). 17259978 10.1038/nchembio860

[133] Huang, V. et al. RNAa is conserved in mammalian cells. *PLoS One* **5**, e8848 (2010). 20107511 10.1371/journal.pone.0008848 PMC2809750

[134] Li, C. et al. Upregulation of E‑cadherin expression mediated by a novel dsRNA suppresses the growth and metastasis of bladder cancer cells by inhibiting beta-catenin/TCF target genes. *Int. J. Oncol.* **52**, 1815–1826 (2018). 29620261 10.3892/ijo.2018.4346 PMC5919711

[135] Voutila, J. et al. Gene expression profile changes after short-activating RNA-mediated induction of endogenous pluripotency factors in human mesenchymal stem cells. *Mol. Ther. Nucl. Acids* **1**, e35 (2012). 10.1038/mtna.2012.20 PMC3437803 23344177

[136] Zhang, M. et al. saKLK1-374 is more difficult to induce KLK1 expression in normal prostate cell lines than that in prostate cancer cell lines: Rethinking the universality of RNA activation. *Biochem. Biophys. Res. Commun.* **643**, 157–168 (2023). 36610381 10.1016/j.bbrc.2022.12.075

[137] Li, B. & Li, C. Suppression of prostate cancer metastasis by DPYSL3-targeted saRNA. *Adv. Exp. Med. Biol.* **983**, 207–216 (2017). 28639202 10.1007/978-981-10-4310-9_15

[138] Yang, K. et al. Antitumor activity of small activating RNAs induced PAWR gene activation in human bladder cancer cells. *Int. J. Med. Sci.* **18**, 3039–3049 (2021). 34220332 10.7150/ijms.60399 PMC8241776

[139] Kang, M. R. et al. Development of therapeutic dsP21-322 for cancer treatment. *Adv. Exp. Med. Biol.* **983**, 217–229 (2017). 28639203 10.1007/978-981-10-4310-9_16

[140] Zhang, Q. et al. p21CIP/WAF1 saRNA inhibits proliferative vitreoretinopathy in a rabbit model. *PLoS One* **18**, e0282063 (2023). 36821623 10.1371/journal.pone.0282063 PMC9949646

[141] Tan, C. P. et al. RNA activation-a novel approach to therapeutically upregulate gene transcription. *Molecules* **26**, 6530 (2021). 34770939 10.3390/molecules26216530 PMC8586927

[142] Sarker, D. et al. MTL-CEBPA, a small activating RNA therapeutic upregulating C/EBP-alpha, in patients with advanced liver cancer: a first-in-human, multicenter, open-label, phase I trial. *Clin. Cancer Res.* **26**, 3936–3946 (2020). 32357963 10.1158/1078-0432.CCR-20-0414

[143] Hashimoto, A. et al. Upregulation of C/EBPalpha inhibits suppressive activity of myeloid cells and potentiates antitumor response in mice and patients with cancer. *Clin. Cancer Res.* **27**, 5961–5978 (2021). 34407972 10.1158/1078-0432.CCR-21-0986 PMC8756351

[144] Reebye, V. et al. Gene activation of CEBPA using saRNA: preclinical studies of the first in human saRNA drug candidate for liver cancer. *Oncogene* **37**, 3216–3228 (2018). 29511346 10.1038/s41388-018-0126-2 PMC6013054

[145] Jarvelainen, H. et al. Preclinical development of RAG1-40-31L: a novel small activating RNA-lipid conjugate targeting tumor suppressor gene p21 for treatment of non-muscle invasive bladder cancer. *J. Clin. Oncol.* **41**, e16620 (2023).

[146] Ishino, Y. et al. Nucleotide sequence of the iap gene, responsible for alkaline phosphatase isozyme conversion in Escherichia coli, and identification of the gene product. *J. Bacteriol.* **169**, 5429–5433 (1987). 3316184 10.1128/jb.169.12.5429-5433.1987 PMC213968

[147] Barrangou, R. et al. CRISPR provides acquired resistance against viruses in prokaryotes. *Science* **315**, 1709–1712 (2007). 17379808 10.1126/science.1138140

[148] Makarova, K. S., Wolf, Y. I. & Koonin, E. V. Comparative genomics of defense systems in archaea and bacteria. *Nucleic Acids Res* **41**, 4360–4377 (2013). 23470997 10.1093/nar/gkt157 PMC3632139

[149] Charpentier, E. & Marraffini, L. A. Harnessing CRISPR-Cas9 immunity for genetic engineering. *Curr. Opin. Microbiol.* **19**, 114–119 (2014). 25048165 10.1016/j.mib.2014.07.001 PMC4155128

[150] Cox, D. B. T. et al. RNA editing with CRISPR-Cas13. *Science* **358**, 1019–1027 (2017). 29070703 10.1126/science.aaq0180 PMC5793859

[151] Ghaemi, A. et al. CRISPR-cas9 genome editing delivery systems for targeted cancer therapy. *Life Sci.* **267**, 118969 (2021). 33385410 10.1016/j.lfs.2020.118969

[152] Herrera-Carrillo, E., Gao, Z. & Berkhout, B. CRISPR therapy towards an HIV cure. *Brief. Funct. Genomics* **19**, 201–208 (2020). 31711197 10.1093/bfgp/elz021 PMC7239311

[153] Li, Y. et al. Ex vivo cell-based CRISPR/Cas9 genome editing for therapeutic applications. *Biomaterials* **234**, 119711 (2020). 31945616 10.1016/j.biomaterials.2019.119711 PMC7035593

[154] Hsu, P. D. et al. DNA targeting specificity of RNA-guided Cas9 nucleases. *Nat. Biotechnol.* **31**, 827–832 (2013). 23873081 10.1038/nbt.2647 PMC3969858

[155] Zhang, F., Wen, Y. & Guo, X. CRISPR/Cas9 for genome editing: progress, implications and challenges. *Hum. Mol. Genet.* **23**, R40–R46 (2014). 24651067 10.1093/hmg/ddu125

[156] Savic, N. & Schwank, G. Advances in therapeutic CRISPR/Cas9 genome editing. *Transl. Res.* **168**, 15–21 (2016). 26470680 10.1016/j.trsl.2015.09.008

[157] Lino, C. A., Harper, J. C., Carney, J. P. & Timlin, J. A. Delivering CRISPR: a review of the challenges and approaches. *Drug Deliv.* **25**, 1234–1257 (2018). 29801422 10.1080/10717544.2018.1474964 PMC6058482

[158] Wang, J. Y. & Doudna, J. A. CRISPR technology: a decade of genome editing is only the beginning. *Science* **379**, eadd8643 (2023). 36656942 10.1126/science.add8643

[159] Zhang, X. et al. Robust genome editing in adult vascular endothelium by nanoparticle delivery of CRISPR-Cas9 plasmid DNA. *Cell Rep.* **38**, 110196 (2022). 34986352 10.1016/j.celrep.2021.110196 PMC8769807

[160] Zhang, S., Shen, J., Li, D. & Cheng, Y. Strategies in the delivery of Cas9 ribonucleoprotein for CRISPR/Cas9 genome editing. *Theranostics* **11**, 614–648 (2021). 33391496 10.7150/thno.47007 PMC7738854

[161] Taha, E. A., Lee, J. & Hotta, A. Delivery of CRISPR-Cas tools for in vivo genome editing therapy: trends and challenges. *J. Control. Release* **342**, 345–361 (2022). 35026352 10.1016/j.jconrel.2022.01.013

[162] Chen, G. et al. A biodegradable nanocapsule delivers a Cas9 ribonucleoprotein complex for in vivo genome editing. *Nat. Nanotechnol.* **14**, 974–980 (2019). 31501532 10.1038/s41565-019-0539-2 PMC6778035

[163] Mirjalili Mohanna, S. Z. et al. LNP-mediated delivery of CRISPR RNP for wide-spread in vivo genome editing in mouse cornea. *J. Control. Release* **350**, 401–413 (2022). 36029893 10.1016/j.jconrel.2022.08.042

[164] Luther, D. C. et al. Delivery approaches for CRISPR/Cas9 therapeutics in vivo: advances and challenges. *Expert Opin. Drug Deliv.* **15**, 905–913 (2018). 30169977 10.1080/17425247.2018.1517746 PMC6295289

[165] Hoy, S. M. Exagamglogene autotemcel: first approval. *Mol. Diagn. Ther.* **28**, 133–139 (2024). 38228954 10.1007/s40291-024-00696-z

[166] Philippidis, A. CASGEVY makes history as FDA approves first CRISPR/Cas9 genome edited therapy. *Hum. Gene Ther.* **35**, 1–4 (2024). 38231658 10.1089/hum.2023.29263.bfs

[167] Wang, T. et al. Three decades of nucleic acid aptamer technologies: lessons learned, progress and opportunities on aptamer development. *Biotechnol. Adv.* **37**, 28–50 (2019). 30408510 10.1016/j.biotechadv.2018.11.001

[168] Perret, G. & Boschetti, E. Aptamer-based affinity chromatography for protein extraction and purification. *Adv. Biochem. Eng. Biotechnol.* **174**, 93–139 (2020). 31485702 10.1007/10_2019_106

[169] Bunka, D. H., Platonova, O. & Stockley, P. G. Development of aptamer therapeutics. *Curr. Opin. Pharmacol.* **10**, 557–562 (2010). 20638902 10.1016/j.coph.2010.06.009

[170] Costello, A. M. et al. Selection and characterization of vimentin-binding aptamer motifs for ovarian cancer. *Molecules* **26**, 6525 (2021). 34770931 10.3390/molecules26216525 PMC8588432

[171] Ren, W. et al. Nanotechnology lighting the way for gene therapy in ophthalmopathy: from opportunities toward applications. *Molecules* **28**, 3500 (2023). 37110734 10.3390/molecules28083500 PMC10141718

[172] Hermann, T. & Patel, D. J. Adaptive recognition by nucleic acid aptamers. *Science* **287**, 820–825 (2000). 10657289 10.1126/science.287.5454.820

[173] Nimjee, S. M., Rusconi, C. P. & Sullenger, B. A. Aptamers: an emerging class of therapeutics. *Annu. Rev. Med.* **56**, 555–583 (2005). 15660527 10.1146/annurev.med.56.062904.144915

[174] Tuerk, C. & Gold, L. Systematic evolution of ligands by exponential enrichment: RNA ligands to bacteriophage T4 DNA polymerase. *Science* **249**, 505–510 (1990). 2200121 10.1126/science.2200121

[175] Ellington, A. D. & Szostak, J. W. In vitro selection of RNA molecules that bind specific ligands. *Nature* **346**, 818–822 (1990). 1697402 10.1038/346818a0

[176] Wang, Q. et al. An efficient and universal in silico screening strategy for acquisition of high-affinity aptamer and its application in analytical utility. *Talanta* **269**, 125535 (2024). 38091739 10.1016/j.talanta.2023.125535

[177] Lin, Y. et al. A modified SELEX approach to identify DNA aptamers with binding specificity to the major histocompatibility complex presenting ovalbumin model antigen. *RSC Adv.* **13**, 32681–32693 (2023). 37936644 10.1039/d3ra04686a PMC10626974

[178] Cao, J., Zhang, F. & Xiong, W. Discovery of aptamers and the acceleration of the development of targeting research in ophthalmology. *Int. J. Nanomed.* **18**, 4421–4430 (2023). 10.2147/IJN.S418115 PMC10404440 37551274

[179] Doherty, C., Wilbanks, B., Khatua, S. & Maher, L. J. Aptamers in neuro-oncology: an emerging therapeutic modality. *Neuro Oncol.* **26**, 38–54 (2024). 37619244 10.1093/neuonc/noad156 PMC10768989

[180] Thomas, B. J. et al. Targeting lung cancer with clinically relevant EGFR mutations using anti-EGFR RNA aptamer. *Mol. Ther. Nucl. Acids* **34**, 102046 (2023). 10.1016/j.omtn.2023.102046 PMC10589377 37869258

[181] Ng, E. W. et al. Pegaptanib, a targeted anti-VEGF aptamer for ocular vascular disease. *Nat. Rev. Drug. Disc.* **5**, 123–132 (2006). 10.1038/nrd1955 16518379

[182] Kim, B. J. et al. Targeting complement components C3 and C5 for the retina: key concepts and lingering questions. *Prog. Retin. Eye. Res.* **83**, 100936 (2021). 33321207 10.1016/j.preteyeres.2020.100936 PMC8197769

[183] Rosenberg, J. E. et al. A phase II trial of AS1411 (a novel nucleolin-targeted DNA aptamer) in metastatic renal cell carcinoma. *Investig. N. Drugs* **32**, 178–187 (2014). 10.1007/s10637-013-0045-6 PMC4560460 24242861

[184] Cheng, Y. et al. AS1411-induced growth inhibition of glioma cells by up-regulation of p53 and down-regulation of Bcl-2 and Akt1 via nucleolin. *PLoS One* **11**, e0167094 (2016). 27907160 10.1371/journal.pone.0167094 PMC5132312

[185] Rizzieri, D. et al. Long-term outcomes of responders in a randomized, controlled phase II trial of aptamer AS1411 in AML. *J. Clin. Oncol.* **28**, 6557 (2010).

[186] Ali, G. K., Algethami, F. K. & Omer, K. M. Gold single atom-based aptananozyme as an ultrasensitive and selective colorimetric probe for detection of thrombin and C-reactive protein. *Mikrochim Acta* **191**, 59 (2023). 38153560 10.1007/s00604-023-06147-6

[187] Tavassoli, M. et al. Aptamer-modified metal organic frameworks for measurement of food contaminants: a review. *Microchim. Acta* **190**, 371 (2023). 10.1007/s00604-023-05937-2 37646854

[188] Narwade, M. et al. Advanced cancer targeting using aptamer functionalized nanocarriers for site-specific cargo delivery. *Biomater. Res.* **27**, 42 (2023). 37149607 10.1186/s40824-023-00365-y PMC10164340

[189] Vlatkovic, I. Non-immunotherapy application of LNP-mRNA: maximizing efficacy and safety. *Biomedicines* **9**, 530 (2021). 34068715 10.3390/biomedicines9050530 PMC8151051

[190] Lee, Y. et al. Immunogenicity of lipid nanoparticles and its impact on the efficacy of mRNA vaccines and therapeutics. *Exp. Mol. Med.* **55**, 2085–2096 (2023). 37779140 10.1038/s12276-023-01086-x PMC10618257

[191] Ali, S. et al. Design of a new cell penetrating peptide for DNA, siRNA and mRNA delivery. *J. Gene Med.* **24**, e3401 (2022). 34856643 10.1002/jgm.3401

[192] Riley, R. S. et al. Ionizable lipid nanoparticles for in utero mRNA delivery. *Sci. Adv.* **7**, eaba1028 (2021). 33523869 10.1126/sciadv.aba1028 PMC7806221

[193] Swingle, K. L. et al. Amniotic fluid stabilized lipid nanoparticles for in utero intra-amniotic mRNA delivery. *J. Control. Release* **341**, 616–633 (2022). 34742747 10.1016/j.jconrel.2021.10.031 PMC8776620

[194] An, D. et al. Systemic messenger RNA therapy as a treatment for methylmalonic acidemia. *Cell Rep.* **21**, 3548–3558 (2017). 29262333 10.1016/j.celrep.2017.11.081 PMC9667413

[195] Gurung, S. et al. mRNA therapy corrects defective glutathione metabolism and restores ureagenesis in preclinical argininosuccinic aciduria. *Sci. Transl. Med.* **16**, eadh1334 (2024). 38198573 10.1126/scitranslmed.adh1334 PMC7615535

[196] Seker Yilmaz, B. & Gissen, P. Genetic therapy approaches for ornithine transcarbamylase deficiency. *Biomedicines* **11**, 2227 (2023). 37626723 10.3390/biomedicines11082227 PMC10452060

[197] Attarwala, H. et al. Translational pharmacokinetic/pharmacodynamic model for mRNA-3927, an investigational therapeutic for the treatment of propionic acidemia. *Nucleic Acid Ther.* **33**, 141–147 (2023). 36577040 10.1089/nat.2022.0036 PMC10066765

[198] Verbeke, R., Lentacker, I., De Smedt, S. C. & Dewitte, H. The dawn of mRNA vaccines: the COVID-19 case. *J. Control. Release* **333**, 511–520 (2021). 33798667 10.1016/j.jconrel.2021.03.043 PMC8008785

[199] Lamb, Y. N. BNT162b2 mRNA COVID-19 vaccine: first approval. *Drugs* **81**, 495–501 (2021). 33683637 10.1007/s40265-021-01480-7 PMC7938284

[200] Zhang, N. N. et al. A thermostable mRNA vaccine against COVID-19. *Cell* **182**, 1271–1283 (2020). 32795413 10.1016/j.cell.2020.07.024 PMC7377714

[201] Liu, X. et al. Safety and superior immunogenicity of heterologous boosting with an RBD-based SARS-CoV-2 mRNA vaccine in Chinese adults. *Cell Res* **32**, 777–780 (2022). 35701541 10.1038/s41422-022-00681-3 PMC9197092

[202] Bahl, K. et al. Preclinical and clinical demonstration of immunogenicity by mRNA vaccines against H10N8 and H7N9 influenza viruses. *Mol. Ther.* **30**, 2874 (2022). 35921837 10.1016/j.ymthe.2022.07.013 PMC9372314

[203] Feldman, R. A. et al. mRNA vaccines against H10N8 and H7N9 influenza viruses of pandemic potential are immunogenic and well tolerated in healthy adults in phase 1 randomized clinical trials. *Vaccine* **37**, 3326–3334 (2019). 31079849 10.1016/j.vaccine.2019.04.074

[204] Aliprantis, A. O. et al. A phase 1, randomized, placebo-controlled study to evaluate the safety and immunogenicity of an mRNA-based RSV prefusion F protein vaccine in healthy younger and older adults. *Hum. Vaccines Immunother.* **17**, 1248–1261 (2021). 10.1080/21645515.2020.1829899 PMC8078688 33121346

[205] Aldrich, C. et al. Proof-of-concept of a low-dose unmodified mRNA-based rabies vaccine formulated with lipid nanoparticles in human volunteers: a phase 1 trial. *Vaccine* **39**, 1310–1318 (2021). 33487468 10.1016/j.vaccine.2020.12.070 PMC7825876

[206] Lorentzen, C. L., Haanen, J. B., Met, O. & Svane, I. M. Clinical advances and ongoing trials on mRNA vaccines for cancer treatment. *Lancet Oncol.* **23**, e450–e458 (2022). 36174631 10.1016/S1470-2045(22)00372-2 PMC9512276

[207] Vishweshwaraiah, Y. L. & Dokholyan, N. V. mRNA vaccines for cancer immunotherapy. *Front. Immunol.* **13**, 1029069 (2022). 36591226 10.3389/fimmu.2022.1029069 PMC9794995

[208] Wei, J. & Hui, A. M. The paradigm shift in treatment from Covid-19 to oncology with mRNA vaccines. *Cancer Treat. Rev.* **107**, 102405 (2022). 35576777 10.1016/j.ctrv.2022.102405 PMC9068246

[209] Dou, H. H. et al. An automated high-throughput fluorescence in situ hybridization (FISH) assay platform for use in the identification and optimization of siRNA-based therapeutics. *SLAS Discov.* **26**, 281–291 (2021). 33016168 10.1177/2472555220960045

[210] Sherman, M. & Contreras, L. Computational approaches in design of nucleic acid-based therapeutics. *Curr. Opin. Biotech.* **53**, 232–239 (2018). 29562215 10.1016/j.copbio.2017.12.001

[211] Dai, H. et al. Pancreatic cancer: nucleic acid drug discovery and targeted therapy. *Front. Cell Dev. Biol.* **10**, 855474 (2022). 35652096 10.3389/fcell.2022.855474 PMC9149368

[212] Kohlberger, M. & Gadermaier, G. SELEX: critical factors and optimization strategies for successful aptamer selection. *Biotechnol. Appl. Bioc.* **69**, 1771–1792 (2022). 10.1002/bab.2244 PMC9788027 34427974

[213] Metkar, M., Pepin, C. S. & Moore, M. J. Tailor made: the art of therapeutic mRNA design. *Nat. Rev. Drug. Disc.* **23**, 67–83 (2024). 10.1038/s41573-023-00827-x 38030688

[214] Kawamoto, Y., Wu, Y., Takahashi, Y. & Takakura, Y. Development of nucleic acid medicines based on chemical technology. *Adv. Drug Deliv. Rev.* **199**, 114872 (2023). 37244354 10.1016/j.addr.2023.114872

[215] Leppek, K. et al. Combinatorial optimization of mRNA structure, stability, and translation for RNA-based therapeutics. *Nat. Commun.* **13**, 1536 (2022). 35318324 10.1038/s41467-022-28776-w PMC8940940

[216] Jiang, X. et al. Oral delivery of nucleic acid therapeutics: challenges, strategies, and opportunities. *Drug. Discov. Today* **28**, 103507 (2023). 36690175 10.1016/j.drudis.2023.103507

[217] Lu, M. et al. Overcoming pharmaceutical bottlenecks for nucleic acid drug development. *Acc. Chem. Res.* **56**, 224–236 (2023). 36624086 10.1021/acs.accounts.2c00464

[218] Zhang, Z. et al. Nucleic acid-based therapy for brain cancer: challenges and strategies. *J. Control. Release* **350**, 80–92 (2022). 35970297 10.1016/j.jconrel.2022.08.014

[219] Weng, Y. et al. The challenge and prospect of mRNA therapeutics landscape. *Biotechnol. Adv.* **40**, 107534 (2020). 32088327 10.1016/j.biotechadv.2020.107534

[220] Kawabata, K., Takakura, Y. & Hashida, M. The fate of plasmid DNA after intravenous injection in mice: involvement of scavenger receptors in its hepatic uptake. *Pharm. Res.* **12**, 825–830 (1995). 7667185 10.1023/a:1016248701505

[221] Huang, X. et al. The landscape of mRNA nanomedicine. *Nat. Med.* **28**, 2273–2287 (2022). 36357682 10.1038/s41591-022-02061-1

[222] Lechardeur, D. & Lukacs, G. L. Intracellular barriers to non-viral gene transfer. *Curr. Gene Ther.* **2**, 183–194 (2002). 12109215 10.2174/1566523024605609

[223] Eygeris, Y., Gupta, M., Kim, J. & Sahay, G. Chemistry of lipid nanoparticles for RNA delivery. *Acc. Chem. Res.* **55**, 2–12 (2022). 34850635 10.1021/acs.accounts.1c00544

[224] Sasso, J. M. et al. The progress and promise of RNA medicine horizontal line an arsenal of targeted treatments. *J. Med. Chem.* **65**, 6975–7015 (2022). 35533054 10.1021/acs.jmedchem.2c00024 PMC9115888

[225] Shinohara, F. et al. siRNA potency enhancement via chemical modifications of nucleotide bases at the 5’-end of the siRNA guide strand. *RNA* **27**, 163–173 (2021). 33177188 10.1261/rna.073783.119 PMC7812868

[226] Mckenzie, L. K. et al. Recent progress in non-native nucleic acid modifications. *Chem. Soc. Rev.* **50**, 5126–5164 (2021). 33644787 10.1039/d0cs01430c

[227] Crooke, S. T., Baker, B. F., Crooke, R. M. & Liang, X. H. Antisense technology: an overview and prospectus. *Nat. Rev. Drug Discov.* **20**, 427–453 (2021). 33762737 10.1038/s41573-021-00162-z

[228] Wang, J., Tian, T., Li, X. & Zhang, Y. Noncoding RNAs emerging as drugs or drug targets: their chemical modification, bio-conjugation and intracellular regulation. *Molecules* **27**, 6717 (2022). 36235253 10.3390/molecules27196717 PMC9573214

[229] Bege, M. & Borbas, A. The medicinal chemistry of artificial nucleic acids and therapeutic oligonucleotides. *Pharm. (Basel)* **15**, 909 (2022). 10.3390/ph15080909 PMC9330994 35893733

[230] Zheng, Y. Y., Wu, Y., Begley, T. J. & Sheng, J. Sulfur modification in natural RNA and therapeutic oligonucleotides. *RSC Chem. Biol.* **2**, 990–1003 (2021). 34458821 10.1039/d1cb00038a PMC8341892

[231] Herkt, M. & Thum, T. Pharmacokinetics and proceedings in clinical application of nucleic acid therapeutics. *Mol. Ther.* **29**, 521–539 (2021). 33188937 10.1016/j.ymthe.2020.11.008 PMC7854291

[232] Crooke, S. T., Vickers, T. A. & Liang, X. H. Phosphorothioate modified oligonucleotide-protein interactions. *Nucleic Acids Res* **48**, 5235–5253 (2020). 32356888 10.1093/nar/gkaa299 PMC7261153

[233] Vasquez, G. et al. Evaluation of phosphorus and non-phosphorus neutral oligonucleotide backbones for enhancing therapeutic index of gapmer antisense oligonucleotides. *Nucleic Acid Ther.* **32**, 40–50 (2022). 34698585 10.1089/nat.2021.0064

[234] Vitravene Study, G. Safety of intravitreous fomivirsen for treatment of cytomegalovirus retinitis in patients with AIDS. *Am. J. Ophthalmol.* **133**, 484–498 (2002). 11931782 10.1016/s0002-9394(02)01332-6

[235] Chen, S. et al. Systematic evaluation of 2’-fluoro modified chimeric antisense oligonucleotide-mediated exon skipping in vitro. *Sci. Rep.* **9**, 6078 (2019). 30988454 10.1038/s41598-019-42523-0 PMC6465270

[236] Gangopadhyay, S. & Gore, K. R. Advances in siRNA therapeutics and synergistic effect on siRNA activity using emerging dual ribose modifications. *RNA Biol.* **19**, 452–467 (2022). 35352626 10.1080/15476286.2022.2052641 PMC8973385

[237] Goswami, A., Prasad, A. K., Maity, J. & Khaneja, N. Synthesis and applications of bicyclic sugar modified locked nucleic acids: a review. *Nucleosides Nucleotides Nucleic Acids* **41**, 503–529 (2022). 35319343 10.1080/15257770.2022.2052316

[238] Nielsen, K. E. et al. NMR studies of fully modified locked nucleic acid (LNA) hybrids: solution structure of an LNA:RNA hybrid and characterization of an LNA:DNA hybrid. *Bioconjugate Chem.* **15**, 449–457 (2004). 10.1021/bc034145h 15149171

[239] Kamali, M. J. et al. Locked nucleic acid (LNA): a modern approach to cancer diagnosis and treatment. *Exp. Cell Res.* **423**, 113442 (2023). 36521777 10.1016/j.yexcr.2022.113442

[240] Pal, R., Deb, I., Sarzynska, J. & Lahiri, A. LNA-induced dynamic stability in a therapeutic aptamer: insights from molecular dynamics simulations. *J. Biomol. Struct. Dyn.* **41**, 2221–2230 (2023). 35100936 10.1080/07391102.2022.2029567

[241] Roxo, C. & Pasternak, A. Changes in physicochemical and anticancer properties modulated by chemically modified sugar moieties within sequence-related G-quadruplex structures. *PLoS One* **17**, e0273528 (2022). 35998148 10.1371/journal.pone.0273528 PMC9397905

[242] Pasternak, A. & Wengel, J. Unlocked nucleic acid-an RNA modification with broad potential. *Org. Biomol. Chem.* **9**, 3591–3597 (2011). 21431171 10.1039/c0ob01085e

[243] Werk, D. et al. Application of small interfering RNAs modified by unlocked nucleic acid (UNA) to inhibit the heart-pathogenic coxsackievirus B3. *FEBS Lett.* **584**, 591–598 (2010). 20005874 10.1016/j.febslet.2009.12.007

[244] Snead, N. M., Escamilla-Powers, J. R., Rossi, J. J. & Mccaffrey, A. P. 5’ unlocked nucleic acid modification improves siRNA targeting. *Mol. Ther. Nucl. Acids* **2**, e103 (2013). 10.1038/mtna.2013.36 PMC3732871 23820891

[245] Yoshida, T. et al. Identification of nucleobase chemical modifications that reduce the hepatotoxicity of gapmer antisense oligonucleotides. *Nucleic Acids Res* **50**, 7224–7234 (2022). 35801870 10.1093/nar/gkac562 PMC9303313

[246] Svitkin, Y. V. et al. N1-methyl-pseudouridine in mRNA enhances translation through eIF2alpha-dependent and independent mechanisms by increasing ribosome density. *Nucleic Acids Res* **45**, 6023–6036 (2017). 28334758 10.1093/nar/gkx135 PMC5449617

[247] Nance, K. D. et al. Cytidine acetylation yields a hypoinflammatory synthetic messenger RNA. *Cell Chem. Bio.* **29**, 312–320 (2022). 35180432 10.1016/j.chembiol.2021.07.003 PMC10370389

[248] Wang, Y. et al. Detection and application of 5-formylcytosine and 5-formyluracil in DNA. *Acc. Chem. Res.* **52**, 1016–1024 (2019). 30666870 10.1021/acs.accounts.8b00543

[249] Koseki, J. et al. Theoretical analyses and experimental validation of the effects caused by the fluorinated substituent modification of DNA. *Sci. Rep.* **10**, 1138 (2020). 31980685 10.1038/s41598-020-57899-7 PMC6981298

[250] Sun, H., Li, K., Liu, C. & Yi, C. Regulation and functions of non-m(6)A mRNA modifications. *Nat. Rev. Mol. Cell Biol.* **24**, 714–731 (2023). 37369853 10.1038/s41580-023-00622-x

[251] Kariko, K., Muramatsu, H., Ludwig, J. & Weissman, D. Generating the optimal mRNA for therapy: HPLC purification eliminates immune activation and improves translation of nucleoside-modified, protein-encoding mRNA. *Nucleic Acids Res* **39**, e142 (2011). 21890902 10.1093/nar/gkr695 PMC3241667

[252] Andries, O. et al. N(1)-methylpseudouridine-incorporated mRNA outperforms pseudouridine-incorporated mRNA by providing enhanced protein expression and reduced immunogenicity in mammalian cell lines and mice. *J. Control. Release* **217**, 337–344 (2015). 26342664 10.1016/j.jconrel.2015.08.051

[253] Pantazopoulou, V. I. et al. From the argonauts mythological sailors to the argonautes RNA-silencing navigators: their emerging roles in human-cell pathologies. *Int. J. Mol. Sci.* **21**, 4007 (2020). 32503341 10.3390/ijms21114007 PMC7312461

[254] Choung, S. et al. Chemical modification of siRNAs to improve serum stability without loss of efficacy. *Biochem. Biophys. Res. Commun.* **342**, 919–927 (2006). 16598842 10.1016/j.bbrc.2006.02.049

[255] Hammond, S. M. et al. Delivery of oligonucleotide-based therapeutics: challenges and opportunities. *EMBO Mol. Med.* **13**, e13243 (2021). 33821570 10.15252/emmm.202013243 PMC8033518

[256] Chen, C., Yang, Z. & Tang, X. Chemical modifications of nucleic acid drugs and their delivery systems for gene-based therapy. *Med. Res. Rev.* **38**, 829–869 (2018). 29315675 10.1002/med.21479

[257] Kuwayama, H. Peptide nucleic acid as a template for Taq DNA polymerase. *Biochem. Biophys. Res. Commun.* **579**, 76–80 (2021). 34592573 10.1016/j.bbrc.2021.09.057

[258] Gupta, A., Mishra, A. & Puri, N. Peptide nucleic acids: advanced tools for biomedical applications. *J. Biotechnol.* **259**, 148–159 (2017). 28764969 10.1016/j.jbiotec.2017.07.026 PMC7114329

[259] Singh, K. R., Sridevi, P. & Singh, R. P. Potential applications of peptide nucleic acid in biomedical domain. *Eng. Rep.* **2**, e12238 (2020). 32838227 10.1002/eng2.12238 PMC7404446

[260] Chen, W., Dong, B., Liu, W. & Liu, Z. Recent advances in peptide nucleic acids as antibacterial agents. *Curr. Med. Chem.* **28**, 1104–1125 (2021). 32484766 10.2174/0929867327666200602132504

[261] Maksudov, F. et al. Therapeutic phosphorodiamidate morpholino oligonucleotides: physical properties, solution structures, and folding thermodynamics. *Mol. Ther. Nucl. Acids* **31**, 631–647 (2023). 10.1016/j.omtn.2023.02.007 PMC9996446 36910708

[262] Mcdonald, C. M. et al. Open-label evaluation of eteplirsen in patients with duchenne muscular dystrophy amenable to exon 51 skipping: PROMOVI Trial. *J. Neuromuscul. Dis.* **8**, 989–1001 (2021). 34120909 10.3233/JND-210643 PMC8673535

[263] Dhillon, S. Viltolarsen: first approval. *Drugs* **80**, 1027–1031 (2020). 32519222 10.1007/s40265-020-01339-3

[264] Shirley, M. Casimersen: first approval. *Drugs* **81**, 875–879 (2021). 33861387 10.1007/s40265-021-01512-2

[265] Egli, M. & Manoharan, M. Chemistry, structure and function of approved oligonucleotide therapeutics. *Nucleic Acids Res* **51**, 2529–2573 (2023). 36881759 10.1093/nar/gkad067 PMC10085713

[266] Roberts, T. C., Langer, R. & Wood, M. J. A. Advances in oligonucleotide drug delivery. *Nat. Rev. Drug Discov.* **19**, 673–694 (2020). 32782413 10.1038/s41573-020-0075-7 PMC7419031

[267] Gait, M. J. & Agrawal, S. Introduction and history of the chemistry of nucleic acids therapeutics. *Methods Mol. Biol.* **2434**, 3–31 (2022). 35213007 10.1007/978-1-0716-2010-6_1 PMC7612508

[268] Ku, S. H. et al. Chemical and structural modifications of RNAi therapeutics. *Adv. Drug Deliv. Rev.* **104**, 16–28 (2016). 26549145 10.1016/j.addr.2015.10.015

[269] Zong, Y., Lin, Y., Wei, T. & Cheng, Q. Lipid nanoparticle (LNP) enables mRNA delivery for cancer therapy. *Adv. Mater.* **35**, e2303261 (2023). 37196221 10.1002/adma.202303261

[270] Jung, H. N. et al. Lipid nanoparticles for delivery of RNA therapeutics: current status and the role of in vivo imaging. *Theranostics* **12**, 7509–7531 (2022). 36438494 10.7150/thno.77259 PMC9691360

[271] Hald Albertsen, C. et al. The role of lipid components in lipid nanoparticles for vaccines and gene therapy. *Adv. Drug Deliv. Rev.* **188**, 114416 (2022). 35787388 10.1016/j.addr.2022.114416 PMC9250827

[272] Samaridou, E., Heyes, J. & Lutwyche, P. Lipid nanoparticles for nucleic acid delivery: current perspectives. *Adv. Drug Deliv. Rev.* **154-155**, 37–63 (2020). 32526452 10.1016/j.addr.2020.06.002

[273] Vhora, I. et al. Lipid-nucleic acid nanoparticles of novel ionizable lipids for systemic BMP-9 gene delivery to bone-marrow mesenchymal stem cells for osteoinduction. *Int. J. Pharm.* **563**, 324–336 (2019). 30954673 10.1016/j.ijpharm.2019.04.006

[274] Wang, C., Zhang, Y. & Dong, Y. Lipid nanoparticle-mRNA formulations for therapeutic applications. *Acc. Chem. Res.* **54**, 4283–4293 (2021). 34793124 10.1021/acs.accounts.1c00550 PMC10068911

[275] Zhang, Y. et al. Lipids and lipid derivatives for RNA delivery. *Chem. Rev.* **121**, 12181–12277 (2021). 34279087 10.1021/acs.chemrev.1c00244 PMC10088400

[276] Heyes, J., Palmer, L., Bremner, K. & Maclachlan, I. Cationic lipid saturation influences intracellular delivery of encapsulated nucleic acids. *J. Control. Release* **107**, 276–287 (2005). 16054724 10.1016/j.jconrel.2005.06.014

[277] Jayaraman, M. et al. Maximizing the potency of siRNA lipid nanoparticles for hepatic gene silencing in vivo. *Angew. Chem. Int. Ed.* **51**, 8529–8533 (2012). 10.1002/anie.201203263 PMC3470698 22782619

[278] Ferraresso, F. et al. Comparison of DLin-MC3-DMA and ALC-0315 for siRNA delivery to hepatocytes and hepatic stellate cells. *Mol. Pharmaceutics* **19**, 2175–2182 (2022). 10.1021/acs.molpharmaceut.2c00033 PMC9621687 35642083

[279] Zhang, M., Sun, J., Li, M. & Jin, X. Modified mRNA-LNP vaccines confer protection against experimental DENV-2 infection in mice. *Mol. Ther. Methods Clin. Dev.* **18**, 702–712 (2020). 32913878 10.1016/j.omtm.2020.07.013 PMC7452130

[280] Escalona-Rayo, O. et al. In vitro and in vivo evaluation of clinically-approved ionizable cationic lipids shows divergent results between mRNA transfection and vaccine efficacy. *Biomed. Pharmacother.* **165**, 115065 (2023). 37406506 10.1016/j.biopha.2023.115065

[281] Han, X. et al. An ionizable lipid toolbox for RNA delivery. *Nat. Commun.* **12**, 7233 (2021). 34903741 10.1038/s41467-021-27493-0 PMC8668901

[282] Mui, B. L. et al. Influence of polyethylene glycol lipid desorption rates on pharmacokinetics and pharmacodynamics of siRNA lipid nanoparticles. *Mol. Ther. Nucl. Acids* **2**, e139 (2013). 10.1038/mtna.2013.66 PMC3894582 24345865

[283] Miao, L. et al. Synergistic lipid compositions for albumin receptor mediated delivery of mRNA to the liver. *Nat. Commun.* **11**, 2424 (2020). 32415122 10.1038/s41467-020-16248-y PMC7229004

[284] Song, L. Y. et al. Characterization of the inhibitory effect of PEG-lipid conjugates on the intracellular delivery of plasmid and antisense DNA mediated by cationic lipid liposomes. *Biochim. Biophys. Acta* **1558**, 1–13 (2002). 11750259 10.1016/s0005-2736(01)00399-6

[285] Kong, L., Campbell, F. & Kros, A. DePEGylation strategies to increase cancer nanomedicine efficacy. *Nanoscale Horiz.* **4**, 378–387 (2019). 32254090 10.1039/c8nh00417j

[286] Kulkarni, J. A. et al. Lipid nanoparticle technology for clinical translation of siRNA therapeutics. *Acc. Chem. Res.* **52**, 2435–2444 (2019). 31397996 10.1021/acs.accounts.9b00368

[287] Shi, D. et al. To PEGylate or not to PEGylate: immunological properties of nanomedicine’s most popular component, polyethylene glycol and its alternatives. *Adv. Drug Deliv. Rev.* **180**, 114079 (2022). 34902516 10.1016/j.addr.2021.114079 PMC8899923

[288] Kim, J., Eygeris, Y., Gupta, M. & Sahay, G. Self-assembled mRNA vaccines. *Adv. Drug Deliv. Rev.* **170**, 83–112 (2021). 33400957 10.1016/j.addr.2020.12.014 PMC7837307

[289] Liu, S. et al. Membrane-destabilizing ionizable phospholipids for organ-selective mRNA delivery and CRISPR-Cas gene editing. *Nat. Mater.* **20**, 701–710 (2021). 33542471 10.1038/s41563-020-00886-0 PMC8188687

[290] Paunovska, K. et al. Nanoparticles containing oxidized cholesterol deliver mRNA to the liver microenvironment at clinically relevant doses. *Adv. Mater.* **31**, e1807748 (2019). 30748040 10.1002/adma.201807748 PMC6445717

[291] Sebastiani, F. et al. Apolipoprotein E binding drives structural and compositional rearrangement of mRNA-containing lipid nanoparticles. *ACS Nano* **15**, 6709–6722 (2021). 33754708 10.1021/acsnano.0c10064 PMC8155318

[292] Cheng, Q. et al. Selective organ targeting (SORT) nanoparticles for tissue-specific mRNA delivery and CRISPR-Cas gene editing. *Nat. Nanotechnol.* **15**, 313–320 (2020). 32251383 10.1038/s41565-020-0669-6 PMC7735425

[293] Wang, Y. S. et al. mRNA-based vaccines and therapeutics: an in-depth survey of current and upcoming clinical applications. *J. Biomed. Sci.* **30**, 84 (2023). 37805495 10.1186/s12929-023-00977-5 PMC10559634

[294] Kenjo, E. et al. Low immunogenicity of LNP allows repeated administrations of CRISPR-Cas9 mRNA into skeletal muscle in mice. *Nat. Commun.* **12**, 7101 (2021). 34880218 10.1038/s41467-021-26714-w PMC8654819

[295] August, A. et al. A phase 1 trial of lipid-encapsulated mRNA encoding a monoclonal antibody with neutralizing activity against Chikungunya virus. *Nat. Med.* **27**, 2224–2233 (2021). 34887572 10.1038/s41591-021-01573-6 PMC8674127

[296] Kotit, S. Lessons from the first-in-human in vivo CRISPR/Cas9 editing of the TTR gene by NTLA-2001 trial in patients with transthyretin amyloidosis with cardiomyopathy. *Glob. Cardiol. Sci. Pract.* **2023**, e202304 (2023). 37928601 10.21542/gcsp.2023.4 PMC10624232

[297] Zhang, L. et al. Effect of mRNA-LNP components of two globally-marketed COVID-19 vaccines on efficacy and stability. *NPJ Vaccines* **8**, 156 (2023). 37821446 10.1038/s41541-023-00751-6 PMC10567765

[298] Rai, R., Alwani, S. & Badea, I. Polymeric nanoparticles in gene therapy: new avenues of design and optimization for delivery applications. *Polym. (Basel)* **11**, 745 (2019). 10.3390/polym11040745 PMC6523186 31027272

[299] Patnaik, S. & Gupta, K. C. Novel polyethylenimine-derived nanoparticles for in vivo gene delivery. *Expert Opin. Drug Deliv.* **10**, 215–228 (2013). 23252504 10.1517/17425247.2013.744964

[300] Jiang, C. et al. Recent advances in the development of polyethylenimine-based gene vectors for safe and efficient gene delivery. *Expert Opin. Drug Deliv.* **16**, 363–376 (2019). 31007095 10.1080/17425247.2019.1604681

[301] Wang, X., Niu, D., Hu, C. & Li, P. Polyethyleneimine-based nanocarriers for gene delivery. *Curr. Pharm. Des.* **21**, 6140–6156 (2015). 26503146 10.2174/1381612821666151027152907

[302] Zhang, Y. H. et al. Recycling gene carrier with high efficiency and low toxicity mediated by L-cystine-bridged bis(beta-cyclodextrin)s. *Sci. Rep.* **4**, 7471 (2014). 25503268 10.1038/srep07471 PMC4265772

[303] Ke, X. et al. Surface-functionalized PEGylated nanoparticles deliver messenger rna to pulmonary immune cells. *ACS Appl. Mater. Interfaces* **12**, 35835–35844 (2020). 32659078 10.1021/acsami.0c08268 PMC9762545

[304] Chen, J. et al. Peptide-based and polypeptide-based gene delivery systems. *Top. Curr. Chem.* **375**, 32 (2017). 10.1007/s41061-017-0115-x 28281201

[305] Souri, M. et al. Poly-L-lysine/hyaluronan nanocarriers as a novel nanosystem for gene delivery. *J. Microsc.* **287**, 32–44 (2022). 35443072 10.1111/jmi.13107

[306] Djafari, J. et al. Study and preparation of multifunctional poly(L-Lysine)@hyaluronic acid nanopolyplexes for the effective delivery of tumor suppressive mir-34a into triple-negative breast cancer cells. *Mater. (Basel)* **13**, 5309 (2020). 10.3390/ma13235309 PMC7727712 33255217

[307] Li, J. et al. Copolymer of poly(ethylene glycol) and poly(L-lysine) grafting polyethylenimine through a reducible disulfide linkage for siRNA delivery. *Nanoscale* **6**, 1732–1740 (2014). 24346086 10.1039/c3nr05024f

[308] Yang, W. et al. Polymeric Micelles with pH-responsive cross-linked core enhance in vivo mrna delivery. *Pharmaceutics* **14**, 1205 (2022). 35745778 10.3390/pharmaceutics14061205 PMC9231146

[309] Jafari, S., Maleki Dizaj, S. & Adibkia, K. Cell-penetrating peptides and their analogues as novel nanocarriers for drug delivery. *Bioimpacts* **5**, 103–111 (2015). 26191505 10.15171/bi.2015.10 PMC4492185

[310] Debacker, A. J. et al. Delivery of oligonucleotides to the liver with GalNAc: from research to registered therapeutic drug. *Mol. Ther.* **28**, 1759–1771 (2020). 32592692 10.1016/j.ymthe.2020.06.015 PMC7403466

[311] Nair, J. K. et al. Multivalent N-acetylgalactosamine-conjugated siRNA localizes in hepatocytes and elicits robust RNAi-mediated gene silencing. *J. Am. Chem. Soc.* **136**, 16958–16961 (2014). 25434769 10.1021/ja505986a

[312] Cui, H. et al. Liver-targeted delivery of oligonucleotides with N-acetylgalactosamine conjugation. *ACS Omega* **6**, 16259–16265 (2021). 34235295 10.1021/acsomega.1c01755 PMC8246477

[313] Brown, C. R. et al. Investigating the pharmacodynamic durability of GalNAc-siRNA conjugates. *Nucleic Acids Res* **48**, 11827–11844 (2020). 32808038 10.1093/nar/gkaa670 PMC7708070

[314] Abdelaal, A. M. & Kasinski, A. L. Ligand-mediated delivery of RNAi-based therapeutics for the treatment of oncological diseases. *NAR Cancer* **3**, zcab030 (2021). 34316717 10.1093/narcan/zcab030 PMC8291076

[315] Huang, X., Leroux, J. C. & Castagner, B. Well-defined multivalent ligands for hepatocytes targeting via asialoglycoprotein receptor. *Bioconjugate Chem.* **28**, 283–295 (2017). 10.1021/acs.bioconjchem.6b00651 27966887

[316] Scharner, J. et al. Delivery of GalNAc-conjugated splice-switching ASOs to non-hepatic cells through ectopic expression of asialoglycoprotein receptor. *Mol. Ther. Nucl. Acids* **16**, 313–325 (2019). 10.1016/j.omtn.2019.02.024 PMC6453860 30965276

[317] Thangamani, L. et al. GalNAc-siRNA conjugates: prospective tools on the frontier of anti-viral therapeutics. *Pharmacol. Res.* **173**, 105864 (2021). 34474100 10.1016/j.phrs.2021.105864 PMC8405237

[318] Nair, J. K. et al. Impact of enhanced metabolic stability on pharmacokinetics and pharmacodynamics of GalNAc-siRNA conjugates. *Nucleic Acids Res* **45**, 10969–10977 (2017). 28981809 10.1093/nar/gkx818 PMC5737438

[319] Foster, D. J. et al. Advanced siRNA designs further improve in vivo performance of GalNAc-siRNA conjugates. *Mol. Ther.* **26**, 708–717 (2018). 29456020 10.1016/j.ymthe.2017.12.021 PMC5910670

[320] Hassler, M. R. et al. Comparison of partially and fully chemically-modified siRNA in conjugate-mediated delivery in vivo. *Nucleic Acids Res* **46**, 2185–2196 (2018). 29432571 10.1093/nar/gky037 PMC5861422

[321] Parmar, R. G. et al. Facile synthesis, geometry, and 2’-substituent-dependent in vivo activity of 5’-(E)- and 5’-(Z)-vinylphosphonate-modified siRNA conjugates. *J. Med. Chem.* **61**, 734–744 (2018). 29376650 10.1021/acs.jmedchem.7b01147

[322] Janas, M. M. et al. Safety evaluation of 2’-deoxy-2’-fluoro nucleotides in GalNAc-siRNA conjugates. *Nucleic Acids Res* **47**, 3306–3320 (2019). 30820542 10.1093/nar/gkz140 PMC6468299

[323] Westerlind, U. et al. Ligands of the asialoglycoprotein receptor for targeted gene delivery, part 1: synthesis of and binding studies with biotinylated cluster glycosides containing N-acetylgalactosamine. *Glycoconj. J.* **21**, 227–241 (2004). 15486455 10.1023/B:GLYC.0000045095.86867.c0

[324] Rensen, P. C. et al. Determination of the upper size limit for uptake and processing of ligands by the asialoglycoprotein receptor on hepatocytes in vitro and in vivo. *J. Biol. Chem.* **276**, 37577–37584 (2001). 11479285 10.1074/jbc.M101786200

[325] Schmidt, K. et al. Characterizing the effect of GalNAc and phosphorothioate backbone on binding of antisense oligonucleotides to the asialoglycoprotein receptor. *Nucleic Acids Res* **45**, 2294–2306 (2017). 28158620 10.1093/nar/gkx060 PMC5389643

[326] Lee, Y. C. et al. Binding of synthetic oligosaccharides to the hepatic Gal/GalNAc lectin. dependence on fine structural features. *J. Biol. Chem.* **258**, 199–202 (1983). 6848494

[327] Biessen, E. A. et al. Synthesis of cluster galactosides with high affinity for the hepatic asialoglycoprotein receptor. *J. Med. Chem.* **38**, 1538–1546 (1995). 7739012 10.1021/jm00009a014

[328] Kichler, A. & Schuber, F. Versatile synthesis of bi- and tri-antennary galactose ligands: interaction with the Gal/GalNAc receptor of human hepatoma cells. *Glycoconj. J.* **12**, 275–281 (1995). 7496142 10.1007/BF00731330

[329] Huang, Y. Preclinical and clinical advances of GalNAc-decorated nucleic acid therapeutics. *Mol. Ther. Nucl. Acids* **6**, 116–132 (2017). 10.1016/j.omtn.2016.12.003 PMC5363494 28325278

[330] Syed, Y. Y. Nedosiran: first approval. *Drugs* **83**, 1729–1733 (2023). 38060091 10.1007/s40265-023-01976-4 PMC10803381

[331] Xu, H., Liao, C., Liang, S. & Ye, B. C. A novel peptide-equipped exosomes platform for delivery of antisense oligonucleotides. *ACS Appl. Mater. Interfaces* **13**, 10760–10767 (2021). 33621039 10.1021/acsami.1c00016

[332] Crescitelli, R., Lasser, C. & Lotvall, J. Isolation and characterization of extracellular vesicle subpopulations from tissues. *Nat. Protoc.* **16**, 1548–1580 (2021). 33495626 10.1038/s41596-020-00466-1

[333] Delorme-Axford, E. et al. Human placental trophoblasts confer viral resistance to recipient cells. *Proc. Natl Acad. Sci. USA* **110**, 12048–12053 (2013). 23818581 10.1073/pnas.1304718110 PMC3718097

[334] Yang, T. et al. Exosome delivered anticancer drugs across the blood-brain barrier for brain cancer therapy in danio rerio. *Pharm. Res.* **32**, 2003–2114 (2015). 25609010 10.1007/s11095-014-1593-y PMC4520542

[335] Su, S. A. et al. Emerging role of exosome-mediated intercellular communication in vascular remodeling. *Oncotarget* **8**, 25700–25712 (2017). 28147325 10.18632/oncotarget.14878 PMC5421963

[336] Pegtel, D. M. & Gould, S. J. Exosomes. *Annu. Rev. Biochem.* **88**, 487–514 (2019). 31220978 10.1146/annurev-biochem-013118-111902

[337] Wang, J. J. et al. Macrophage-secreted exosomes delivering miRNA-21 inhibitor can regulate BGC-823 cell proliferation. *Asian Pac. J. Cancer Prev.* **16**, 4203–4209 (2015). 26028073 10.7314/apjcp.2015.16.10.4203

[338] Zheng, H. et al. Exosome‑encapsulated miR‑26a attenuates aldosterone‑induced tubulointerstitial fibrosis by inhibiting the CTGF/SMAD3 signaling pathway. *Int. J. Mol. Med.* **51**, 11 (2023). 36524378 10.3892/ijmm.2022.5214 PMC9848436

[339] Hashemi, Z. S. et al. Novel delivery of sorafenib by natural killer cell-derived exosomes-enhanced apoptosis in triple-negative breast cancer. *Nanomed. (Lond.)* **18**, 437–453 (2023). 10.2217/nnm-2022-0237 37199259

[340] Kamerkar, S. et al. Exosome-mediated genetic reprogramming of tumor-associated macrophages by exoASO-STAT6 leads to potent monotherapy antitumor activity. *Sci. Adv.* **8**, eabj7002 (2022). 35179953 10.1126/sciadv.abj7002 PMC8856615

[341] Kaban, K. et al. Therapeutic silencing of BCL-2 using NK cell-derived exosomes as a novel therapeutic approach in breast cancer. *Cancers (Basel)* **13**, 2397 (2021). 34063475 10.3390/cancers13102397 PMC8156181

[342] Wahlgren, J. et al. Plasma exosomes can deliver exogenous short interfering RNA to monocytes and lymphocytes. *Nucleic Acids Res* **40**, e130 (2012). 22618874 10.1093/nar/gks463 PMC3458529

[343] Alvarez-Erviti, L. et al. Delivery of siRNA to the mouse brain by systemic injection of targeted exosomes. *Nat. Biotechnol.* **29**, 341–345 (2011). 21423189 10.1038/nbt.1807

[344] Yuan, D. et al. Macrophage exosomes as natural nanocarriers for protein delivery to inflamed brain. *Biomaterials* **142**, 1–12 (2017). 28715655 10.1016/j.biomaterials.2017.07.011 PMC5603188

[345] Zheng, M. et al. Harnessing exosomes for the development of brain drug delivery systems. *Bioconjugate Chem.* **30**, 994–1005 (2019). 10.1021/acs.bioconjchem.9b00085 30855944

[346] Bunggulawa, E. J. et al. Recent advancements in the use of exosomes as drug delivery systems. *J. Nanobiotechnol.* **16**, 81 (2018). 10.1186/s12951-018-0403-9 PMC6190562 30326899

[347] Rajput, A., Varshney, A., Bajaj, R. & Pokharkar, V. Exosomes as new generation vehicles for drug delivery: biomedical applications and future perspectives. *Molecules* **27**, 7289 (2022). 36364116 10.3390/molecules27217289 PMC9658823

[348] Asadirad, A. et al. Dendritic cell immunotherapy with miR-155 enriched tumor-derived exosome suppressed cancer growth and induced antitumor immune responses in murine model of colorectal cancer induced by CT26 cell line. *Int. Immunopharmacol.* **104**, 108493 (2022). 35032826 10.1016/j.intimp.2021.108493

[349] Xi, X. M., Xia, S. J. & Lu, R. Drug loading techniques for exosome-based drug delivery systems. *Pharmazie* **76**, 61–67 (2021). 33714281 10.1691/ph.2021.0128

[350] Kim, M. S. et al. Development of exosome-encapsulated paclitaxel to overcome MDR in cancer cells. *Nanomedicine* **12**, 655–664 (2016). 26586551 10.1016/j.nano.2015.10.012 PMC4809755

[351] Han, S. et al. Delivery of anti-miRNA-221 for colorectal carcinoma therapy using modified cord blood mesenchymal stem cells-derived exosomes. *Front. Mol. Biosci.* **8**, 743013 (2021). 34616773 10.3389/fmolb.2021.743013 PMC8488275

[352] Luan, X. et al. Engineering exosomes as refined biological nanoplatforms for drug delivery. *Acta Pharmacol. Sin.* **38**, 754–763 (2017). 28392567 10.1038/aps.2017.12 PMC5520184

[353] Rehman, F. U., Liu, Y., Zheng, M. & Shi, B. Exosomes based strategies for brain drug delivery. *Biomaterials* **293**, 121949 (2023). 36525706 10.1016/j.biomaterials.2022.121949

[354] Johnsen, K. B. et al. Evaluation of electroporation-induced adverse effects on adipose-derived stem cell exosomes. *Cytotechnology* **68**, 2125–2138 (2016). 26856590 10.1007/s10616-016-9952-7 PMC5023584

[355] Liang, Y., Duan, L., Lu, J. & Xia, J. Engineering exosomes for targeted drug delivery. *Theranostics* **11**, 3183–3195 (2021). 33537081 10.7150/thno.52570 PMC7847680

[356] Zhang, H. et al. Exosome-mediated targeted delivery of miR-210 for angiogenic therapy after cerebral ischemia in mice. *J. Nanobiotechnol.* **17**, 29 (2019). 10.1186/s12951-019-0461-7 PMC6379944 30782171

[357] Kim, G. et al. Systemic delivery of microRNA-21 antisense oligonucleotides to the brain using T7-peptide decorated exosomes. *J. Control. Release* **317**, 273–281 (2020). 31730913 10.1016/j.jconrel.2019.11.009

[358] Mahati, S. et al. Delivery of miR-26a using an exosomes-based nanosystem inhibited proliferation of hepatocellular carcinoma. *Front. Mol. Biosci.* **8**, 738219 (2021). 34552961 10.3389/fmolb.2021.738219 PMC8450326

[359] Xu, X. et al. Exosome-mediated delivery of kartogenin for chondrogenesis of synovial fluid-derived mesenchymal stem cells and cartilage regeneration. *Biomaterials* **269**, 120539 (2021). 33243424 10.1016/j.biomaterials.2020.120539

[360] Cooper, J. M. et al. Systemic exosomal siRNA delivery reduced alpha-synuclein aggregates in brains of transgenic mice. *Mov. Disord.* **29**, 1476–1485 (2014). 25112864 10.1002/mds.25978 PMC4204174

[361] Ren, X. et al. Exosomal DNA aptamer targeting alpha-synuclein aggregates reduced neuropathological deficits in a mouse parkinson’s disease model. *Mol. Ther. Nucl. Acids* **17**, 726–740 (2019). 10.1016/j.omtn.2019.07.008 PMC6709346 31437653

[362] Fu, Z. et al. In vivo self-assembled small RNAs as a new generation of RNAi therapeutics. *Cell Res* **31**, 631–648 (2021). 33782530 10.1038/s41422-021-00491-z PMC8169669

[363] Couch, Y. et al. A brief history of nearly EV-erything - the rise and rise of extracellular vesicles. *J. Extracell. Vesicles* **10**, e12144 (2021). 34919343 10.1002/jev2.12144 PMC8681215

[364] Erathodiyil, N. & Ying, J. Y. Functionalization of inorganic nanoparticles for bioimaging applications. *Acc. Chem. Res.* **44**, 925–935 (2011). 21648430 10.1021/ar2000327

[365] Meena, J. et al. Inorganic nanoparticles for natural product delivery: a review. *Environ. Chem. Lett.* **18**, 2107–2118 (2020).

[366] Luther, D. C. et al. Delivery of drugs, proteins, and nucleic acids using inorganic nanoparticles. *Adv. Drug Deliv. Rev.* **156**, 188–213 (2020). 32610061 10.1016/j.addr.2020.06.020 PMC8559718

[367] Wang, Z. et al. Gold nanoparticle‑mediated delivery of paclitaxel and nucleic acids for cancer therapy (Review). *Mol. Med. Rep.* **22**, 4475–4484 (2020). 33173972 10.3892/mmr.2020.11580 PMC7646735

[368] Graczyk, A., Pawlowska, R., Jedrzejczyk, D. & Chworos, A. Gold nanoparticles in conjunction with nucleic acids as a modern molecular system for cellular delivery. *Molecules* **25**, 204 (2020). 31947834 10.3390/molecules25010204 PMC6982881

[369] Shrestha, B. et al. Gold nanoparticles mediated drug-gene combinational therapy for breast cancer treatment. *Int. J. Nanomed.* **15**, 8109–8119 (2020). 10.2147/IJN.S258625 PMC7585780 33116521

[370] Jensen, S. A. et al. Spherical nucleic acid nanoparticle conjugates as an RNAi-based therapy for glioblastoma. *Sci. Transl. Med.* **5**, 209ra152 (2013). 24174328 10.1126/scitranslmed.3006839 PMC4017940

[371] Kumthekar, P. et al. A first-in-human phase 0 clinical study of RNA interference-based spherical nucleic acids in patients with recurrent glioblastoma. *Sci. Transl. Med.* **13**, eabb3945 (2021). 33692132 10.1126/scitranslmed.abb3945 PMC8272521

[372] Wu, K. et al. Magnetic nanoparticles in nanomedicine: a review of recent advances. *Nanotechnology* **30**, 502003 (2019). 31491782 10.1088/1361-6528/ab4241

[373] Dash, S. et al. Emerging trends in the nanomedicine applications of functionalized magnetic nanoparticles as novel therapies for acute and chronic diseases. *J. Nanobiotechnol.* **20**, 393 (2022). 10.1186/s12951-022-01595-3 PMC9428876 36045375

[374] Chavan, N., Dharmaraj, D., Sarap, S. & Surve, C. Magnetic nanoparticles–new era in nanotechnology. *J. Drug Deliv. Sci. Technol.* **77**, 103899 (2022).

[375] Luo, M. et al. Delivering the promise of gene therapy with nanomedicines in treating central nervous system diseases. *Adv. Sci.* **9**, e2201740 (2022). 10.1002/advs.202201740 PMC9475540 35851766

[376] Rahamathulla, M. et al. Carbon nanotubes: current perspectives on diverse applications in targeted drug delivery and therapies. *Mater. (Basel)* **14**, 6707 (2021). 10.3390/ma14216707 PMC8588285 34772234

[377] Ren, X. et al. Photoactivatable RNAi for cancer gene therapy triggered by near-infrared-irradiated single-walled carbon nanotubes. *Int. J. Nanomed.* **12**, 7885–7896 (2017). 10.2147/IJN.S141882 PMC5666115 29138556

[378] Levina, A. S., Repkova, M. N., Ismagilov, Z. R. & Zarytova, V. F. Methods of the synthesis of silicon-containing nanoparticles intended for nucleic acid delivery. *Eurasia. Chem.-Techno* **20**, 177–194 (2018).

[379] Stead, S. O. et al. siRNA gene knockdown with functionalised porous silicon nanoparticles. *Transplantation* **104**, s158 (2020).

[380] Luo, M. et al. A multifunctional porous silicon nanocarrier for glioblastoma treatment. *Mol. Pharmaceutics* **20**, 545–560 (2023). 10.1021/acs.molpharmaceut.2c00763 36484477

[381] Goyal, R. et al. Peptide-based delivery vectors with pre-defined geometrical locks. *RSC Med. Chem.* **11**, 1303–1313 (2020). 34095842 10.1039/d0md00229a PMC8126890

[382] Lian, Z. & Ji, T. Functional peptide-based drug delivery systems. *J. Mater. Chem. B* **8**, 6517–6529 (2020). 32350489 10.1039/d0tb00713g

[383] Jeon, B. W. et al. Recent advances in peptide signaling during arabidopsis root development. *J. Exp. Bot.* **72**, 2889–2902 (2021). 33595615 10.1093/jxb/erab050

[384] Kim, J. et al. Oral supplementation of low-molecular-weight collagen peptides reduces skin wrinkles and improves biophysical properties of skin: a randomized, double-blinded, placebo-controlled study. *J. Med. Food* **25**, 1146–1154 (2022). 36516059 10.1089/jmf.2022.K.0097

[385] Lindberg, J., Nilvebrant, J., Nygren, P, A. & Lehmann, F. Progress and future directions with peptide-drug conjugates for targeted cancer therapy. *Molecules* **26**, 6042 (2021). 34641586 10.3390/molecules26196042 PMC8512983

[386] Hao, C. et al. Renovation of old drugs’ – can peptide drug conjugates lead the post-ADC era? *Aust. J. Chem.* **76**, 318–336 (2023).

[387] Wang, J., Tripathy, N. & Chung, E. J. Targeting and therapeutic peptide-based strategies for polycystic kidney disease. *Adv. Drug Deliv. Rev.* **161-162**, 176–189 (2020). 32866560 10.1016/j.addr.2020.08.011 PMC7736157

[388] Feldman, K. S., Pavlou, M. P. & Zahid, M. Cardiac targeting peptide: from identification to validation to mechanism of transduction. *Methods Mol. Biol.* **2211**, 97–112 (2021). 33336273 10.1007/978-1-0716-0943-9_8 PMC8067068

[389] Lu, L. et al. A novel blood-brain barrier-penetrating and vascular-targeting chimeric peptide inhibits glioma angiogenesis. *Int. J. Mol. Sci.* **24**, 8753 (2023). 37240099 10.3390/ijms24108753 PMC10218704

[390] Suzuki, M. et al. Characterization of the membrane penetration-enhancing peptide S19 derived from human syncytin-1 for the intracellular delivery of TAT-fused proteins. *Biochem. Biophys. Res. Commun.* **586**, 63–67 (2022). 34826702 10.1016/j.bbrc.2021.11.065

[391] Vijakumaran, U. et al. Development of cell penetrating peptides for effective delivery of recombinant factors into target cells. *Protein Pept. Lett.* **27**, 1092–1101 (2020). 32484079 10.2174/0929866527666200525164135

[392] Maraming, P. et al. The cationic cell-penetrating KT2 peptide promotes cell membrane defects and apoptosis with autophagy inhibition in human HCT 116 colon cancer cells. *J. Cell. Physiol.* **234**, 22116–22129 (2019). 31073999 10.1002/jcp.28774

[393] Klipp, A., Burger, M. & Leroux, J. C. Get out or die trying: peptide- and protein-based endosomal escape of RNA therapeutics. *Adv. Drug Deliv. Rev.* **200**, 115047 (2023). 37536508 10.1016/j.addr.2023.115047

[394] Guo, Y. et al. Self-assembled peptide nanoparticles with endosome escaping permits for co-drug delivery. *Talanta* **221**, 121572 (2021). 33076119 10.1016/j.talanta.2020.121572

[395] Zhao, Y. et al. Engineered histidine-rich peptides enhance endosomal escape for antibody-targeted intracellular delivery of functional proteins. *Angew. Chem. Int. Ed*. e202304692 (2023). 10.1002/anie.202304692 37283024

[396] Lu, J. et al. Types of nuclear localization signals and mechanisms of protein import into the nucleus. *Cell Commun. Signal.* **19**, 60 (2021). 34022911 10.1186/s12964-021-00741-y PMC8140498

[397] Huang, S. et al. Design of acid-activated cell-penetrating peptides with nuclear localization capacity for anticancer drug delivery. *J. Pept. Sci.* **27**, e3354 (2021). 34101293 10.1002/psc.3354

[398] Kanazawa, T. et al. Electroporation-based ex vivo gene delivery into dendritic cells by anionic polymer-coated versatile nuclear localization signal/pDNA complex. *Biol. Pharm. Bull.* **44**, 1866–1871 (2021). 34853269 10.1248/bpb.b21-00559

[399] Urandur, S. & Sullivan, M. O. Peptide-based vectors: a biomolecular engineering strategy for gene delivery. *Annu. Rev. Chem. Biomol. Eng.* **14**, 243–264 (2023). 36888991 10.1146/annurev-chembioeng-101121-070232

[400] Varanko, A., Saha, S. & Chilkoti, A. Recent trends in protein and peptide-based biomaterials for advanced drug delivery. *Adv. Drug Deliv. Rev.* **156**, 133–187 (2020). 32871201 10.1016/j.addr.2020.08.008 PMC7456198

[401] Falato, L., Gestin, M. & Langel, U. Cell-penetrating peptides delivering siRNAs: an overview. *Methods Mol. Biol.* **2282**, 329–352 (2021). 33928583 10.1007/978-1-0716-1298-9_18

[402] Kim, E. H. et al. PDL1-binding peptide/anti-miRNA21 conjugate as a therapeutic modality for PD-L1(high) tumors and TAMs. *J. Control. Release* **345**, 62–74 (2022). 35263615 10.1016/j.jconrel.2022.02.031

[403] Yang, G. et al. Improved cellular delivery of antisense oligonucleotide for miRNA-21 imaging in vivo using cell-penetrating peptide-based nanoprobes. *Mol. Pharmaceutics* **18**, 787–795 (2021). 10.1021/acs.molpharmaceut.0c00160 33480702

[404] Liu, Q. et al. iRGD-modified exosomes-delivered BCL6 siRNA inhibit the progression of diffuse large B-cell lymphoma. *Front. Oncol.* **12**, 822805 (2022). 35982974 10.3389/fonc.2022.822805 PMC9378967

[405] Khabazian, E. et al. Cationic liposome decorated with cyclic RGD peptide for targeted delivery of anti-STAT3 siRNA to melanoma cancer cells. *J. Drug Target.* **30**, 522–533 (2022). 34482780 10.1080/1061186X.2021.1973481

[406] Liao, L. et al. A bivalent cyclic RGD-siRNA conjugate enhances the antitumor effect of apatinib via co-inhibiting VEGFR2 in non-small cell lung cancer xenografts. *Drug Deliv.* **28**, 1432–1442 (2021). 34236267 10.1080/10717544.2021.1937381 PMC8274511

[407] Liu, X. et al. Tumor-targeted in vivo gene silencing via systemic delivery of cRGD-conjugated siRNA. *Nucleic Acids Res* **42**, 11805–11817 (2014). 25223783 10.1093/nar/gku831 PMC4191406

[408] Wang, Y. et al. Tumor-targeted anti-VEGF RNAi capable of sequentially responding to intracellular microenvironments for potent systemic tumor suppression. *ACS Appl. Bio Mater.* **3**, 9145–9155 (2020). 35019592 10.1021/acsabm.0c01427

[409] Moshnikova, A. et al. Targeting bladder urothelial carcinoma with pHLIP-ICG and inhibition of urothelial cancer cell proliferation by pHLIP-amanitin. *Front Urol.* **2**, 868919 (2022). 36439552 10.3389/fruro.2022.868919 PMC9691284

[410] Zhang, M. et al. In vivo distribution and therapeutic efficacy of radioiodine-labeled pH-low insertion peptide variant 3 in a mouse model of breast cancer. *Mol. Imaging* **2022**, 7456365 (2022). 35903249 10.1155/2022/7456365 PMC9281440

[411] Wyatt, L. C. et al. Applications of pHLIP technology for cancer imaging and therapy. *Trends Biotechnol.* **35**, 653–664 (2017). 28438340 10.1016/j.tibtech.2017.03.014 PMC5492987

[412] Dupont, M. et al. Tumor treatment by pHLIP-targeted antigen delivery. *Front. Bioeng. Biotech.* **10**, 1082290 (2022). 10.3389/fbioe.2022.1082290 PMC9853002 36686229

[413] Visca, H. et al. pHLIP peptides target acidity in activated macrophages. *Mol. Imaging Biol.* **24**, 874–885 (2022). 35604527 10.1007/s11307-022-01737-x PMC9681937

[414] Son, S. M. et al. Therapeutic effect of pHLIP-mediated CEACAM6 gene silencing in lung adenocarcinoma. *Sci. Rep.* **9**, 11607 (2019). 31474761 10.1038/s41598-019-48104-5 PMC6717735

[415] Luna Velez, M. V. et al. Delivery of antisense oligonucleotides for splice-correction of androgen receptor pre-mRNA in castration-resistant prostate cancer models using cell-penetrating peptides. *Prostate* **82**, 657–665 (2022). 35098567 10.1002/pros.24309 PMC9303360

[416] Ervin, E. H. et al. Targeted gene silencing in human embryonic stem cells using cell-penetrating peptide PepFect 14. *Stem Cell Res. Ther.* **10**, 43 (2019). 30678718 10.1186/s13287-019-1144-x PMC6345057

[417] Kurrikoff, K., Vunk, B. & Langel, U. Status update in the use of cell-penetrating peptides for the delivery of macromolecular therapeutics. *Expert Opin. Biol. Ther.* **21**, 361–370 (2021). 32938243 10.1080/14712598.2021.1823368

[418] Taniguchi, K. et al. Alpha-aminoisobutyric acid-containing amphipathic helical peptide-cyclic RGD conjugation as a potential drug delivery system for microRNA replacement therapy in vitro. *Mol. Pharmaceutics* **16**, 4542–4550 (2019). 10.1021/acs.molpharmaceut.9b00680 31596588

[419] Tarvirdipour, S. et al. A self-assembling peptidic platform to boost the cellular uptake and nuclear delivery of oligonucleotides. *Biomater. Sci.* **10**, 4309–4323 (2022). 35771211 10.1039/d2bm00826b

[420] Ji, K., Xiao, Y. & Zhang, W. Acid-activated nonviral peptide vector for gene delivery. *J. Pept. Sci.* **26**, e3230 (2020). 31696619 10.1002/psc.3230

[421] Kwon, E. J., Ko, H. & Bhatia, S. N. Peptide spiders: peptide-polymer conjugates to traffic nucleic acids. *Mol. Pharmaceutics* **17**, 3633–3642 (2020). 10.1021/acs.molpharmaceut.0c00714 PMC7986012 32786959

[422] Kim, G. C., Cheon, D. H. & Lee, Y. Challenge to overcome current limitations of cell-penetrating peptides. *BBA Proteins Proteom.* **1869**, 140604 (2021). 10.1016/j.bbapap.2021.140604 33453413

[423] Hadianamrei, R. & Zhao, X. Current state of the art in peptide-based gene delivery. *J. Control. Release* **343**, 600–619 (2022). 35157938 10.1016/j.jconrel.2022.02.010

[424] Buyanova, M. et al. Discovery of a cyclic cell-penetrating peptide with improved endosomal escape and cytosolic delivery efficiency. *Mol. Pharmaceutics* **19**, 1378–1388 (2022). 10.1021/acs.molpharmaceut.1c00924 PMC9175492 35405068

[425] Molle, L. M., Smyth, C. H., Yuen, D. & Johnston, A. P. R. Nanoparticles for vaccine and gene therapy: overcoming the barriers to nucleic acid delivery. *WIREs Nanomed. Nanobiotechnol.* **14**, e1809 (2022). 10.1002/wnan.1809 PMC9786906 36416028

[426] Alhakamy, N. A., Nigatu, A. S., Berkland, C. J. & Ramsey, J. D. Noncovalently associated cell-penetrating peptides for gene delivery applications. *Ther. Deliv.* **4**, 741–757 (2013). 23738670 10.4155/tde.13.44 PMC4207642

[427] Samec, T. et al. Peptide-based delivery of therapeutics in cancer treatment. *Mater. Today Bio* **14**, 100248 (2022). 35434595 10.1016/j.mtbio.2022.100248 PMC9010702

[428] Burks, S. R. et al. Co-encapsulating the fusogenic peptide INF7 and molecular imaging probes in liposomes increases intracellular signal and probe retention. *PLoS One* **10**, e0120982 (2015). 25816348 10.1371/journal.pone.0120982 PMC4376389

[429] Feng, R., Ni, R. & Chau, Y. Fusogenic peptide modification to enhance gene delivery by peptide-DNA nano-coassemblies. *Biomater. Sci.* **10**, 5116–5120 (2022). 35975695 10.1039/d2bm00705c

[430] Hagino, Y. et al. GALA-modified lipid nanoparticles for the targeted delivery of plasmid dna to the lungs. *Mol. Pharmaceutics* **18**, 878–888 (2021). 10.1021/acs.molpharmaceut.0c00854 33492961

[431] Li, C., Cao, X. W., Zhao, J. & Wang, F. J. Effective therapeutic drug delivery by GALA3, an endosomal escape peptide with reduced hydrophobicity. *J. Membr. Biol.* **253**, 139–152 (2020). 32002589 10.1007/s00232-020-00109-2

[432] Miura, N. et al. A KALA-modified lipid nanoparticle containing CpG-free plasmid DNA as a potential DNA vaccine carrier for antigen presentation and as an immune-stimulative adjuvant. *Nucleic Acids Res* **43**, 1317–1331 (2015). 25605799 10.1093/nar/gkv008 PMC4330373

[433] Dastpeyman, M. et al. Endosomal escape cell-penetrating peptides significantly enhance pharmacological effectiveness and CNS activity of systemically administered antisense oligonucleotides. *Int. J. Pharm.* **599**, 120398 (2021). 33640427 10.1016/j.ijpharm.2021.120398

[434] Alipour, M., Hosseinkhani, S., Sheikhnejad, R. & Cheraghi, R. Nano-biomimetic carriers are implicated in mechanistic evaluation of intracellular gene delivery. *Sci. Rep.* **7**, 41507 (2017). 28128339 10.1038/srep41507 PMC5269746

[435] Samec, T. et al. Fusogenic peptide delivery of bioactive siRNAs targeting CSNK2A1 for treatment of ovarian cancer. *Mol. Ther. Nucl. Acids* **30**, 95–111 (2022). 10.1016/j.omtn.2022.09.012 PMC9530961 36213692

[436] Lu, S. et al. Multi-functional self-assembled nanoparticles for pVEGF-shRNA loading and anti-tumor targeted therapy. *Int. J. Pharm.* **575**, 118898 (2020). 31846730 10.1016/j.ijpharm.2019.118898

[437] Luo, Y., Ma, J. & Lu, W. The significance of mitochondrial dysfunction in cancer. *Int. J. Mol. Sci.* **21**, 5598 (2020). 32764295 10.3390/ijms21165598 PMC7460667

[438] Yao, R. Q., Ren, C., Xia, Z. F. & Yao, Y. M. Organelle-specific autophagy in inflammatory diseases: a potential therapeutic target underlying the quality control of multiple organelles. *Autophagy* **17**, 385–401 (2021). 32048886 10.1080/15548627.2020.1725377 PMC8007140

[439] Machado-Oliveira, G., Ramos, C., Marques, A. R. A. & Vieira, O. V. Cell senescence, multiple organelle dysfunction and atherosclerosis. *Cells* **9**, 2146 (2020). 32977446 10.3390/cells9102146 PMC7598292

[440] Hu, C., Huang, Y. & Chen, Y. Targeted modification of the cationic anticancer peptide HPRP-A1 with iRGD to improve specificity, penetration, and tumor-tissue accumulation. *Mol. Pharmaceutics* **16**, 561–572 (2019). 10.1021/acs.molpharmaceut.8b00854 30592418

[441] Nakamura, M., Fujiwara, K. & Doi, N. Cytoplasmic delivery of siRNA using human-derived membrane penetration-enhancing peptide. *J. Nanobiotechnol.* **20**, 458 (2022). 10.1186/s12951-022-01667-4 PMC9615171 36303212

[442] Bjorge, J. D., Pang, A. & Fujita, D. J. Delivery of gene targeting siRNAs to breast cancer cells using a multifunctional peptide complex that promotes both targeted delivery and endosomal release. *PLoS One* **12**, e0180578 (2017). 28666009 10.1371/journal.pone.0180578 PMC5493434

[443] Ruan, R. et al. Topical and targeted delivery of sirnas to melanoma cells using a fusion peptide carrier. *Sci. Rep.* **6**, 29159 (2016). 27374619 10.1038/srep29159 PMC4931591

[444] Cerrato, C. P. et al. Intracellular delivery of therapeutic antisense oligonucleotides targeting mRNA coding mitochondrial proteins by cell-penetrating peptides. *J. Mater. Chem. B* **8**, 10825–10836 (2020). 33174901 10.1039/d0tb01106a

[445] Kuang, Y. et al. Dual functional peptide-driven nanoparticles for highly efficient glioma-targeting and drug codelivery. *Mol. Pharmaceutics* **13**, 1599–1607 (2016). 10.1021/acs.molpharmaceut.6b00051 27058780

[446] Bulut, S. et al. Slow release and delivery of antisense oligonucleotide drug by self-assembled peptide amphiphile nanofibers. *Biomacromolecules* **12**, 3007–3014 (2011). 21707109 10.1021/bm200641e

[447] Nirasawa, K. et al. Development of A2G80 peptide-gene complex for targeted delivery to muscle cells. *J. Control. Release* **329**, 988–996 (2021). 33091529 10.1016/j.jconrel.2020.10.029

[448] Jafari, M. & Chen, P. Peptide mediated siRNA delivery. *Curr. Top. Med. Chem.* **9**, 1088–1097 (2009). 19860709 10.2174/156802609789630839

[449] Yan Y. Q. et al. Localized instillation enables in vivo screening of targeting peptides using one-bead one-compound technology. *ACS Nano*. 10.1021/acsnano.2c09894 (2023). 10.1021/acsnano.2c09894 36596220

[450] Paray, B. A. et al. The role of the multifunctional antimicrobial peptide melittin in gene delivery. *Drug. Discov. Today* **26**, 1053–1059 (2021). 33450177 10.1016/j.drudis.2021.01.004

[451] Govindarajan, S. et al. Targeting human epidermal growth factor receptor 2 by a cell-penetrating peptide-affibody bioconjugate. *Biomaterials* **33**, 2570–2582 (2012). 22192536 10.1016/j.biomaterials.2011.12.003

[452] Li, Q. et al. Multifunctional peptide-conjugated nanocarriers for pulp regeneration in a full-length human tooth root. *Acta Biomater.* **127**, 252–265 (2021). 33813092 10.1016/j.actbio.2021.03.059 PMC8154711

[453] Wan, Y., Moyle, P. M., Christie, M. P. & Toth, I. Nanosized, peptide-based multicomponent DNA delivery systems: optimization of endosome escape activity. *Nanomed. (Lond.)* **11**, 907–919 (2016). 10.2217/nnm.16.27 26979574

[454] Rohira, H., Arora, A., Kaur, P. & Chugh, A. Peptide cargo administration: current state and applications. *Appl. Microbiol. Biotechnol.* **107**, 3153–3181 (2023). 37052636 10.1007/s00253-023-12512-5 PMC10099029

[455] Liu, Y. et al. Development and characterization of high efficacy cell-penetrating peptide via modulation of the histidine and arginine ratio for gene therapy. *Mater. (Basel)* **14**, 4674 (2021). 10.3390/ma14164674 PMC8399742 34443195

[456] Tsvetkov, V. B. et al. Anticoagulant oligonucleotide-peptide conjugates: identification of thrombin aptamer conjugates with improved characteristics. *Int. J. Mol. Sci.* **23**, 3820 (2022). 35409180 10.3390/ijms23073820 PMC8998821

[457] Kaplan, A. R. et al. Ku80-targeted pH-sensitive peptide-PNA conjugates are tumor selective and sensitize cancer cells to ionizing radiation. *Mol. Cancer Res.* **18**, 873–882 (2020). 32098827 10.1158/1541-7786.MCR-19-0661 PMC7272299

[458] Dutta, K., Das, R., Medeiros, J. & Thayumanavan, S. Disulfide bridging strategies in viral and nonviral platforms for nucleic acid delivery. *Biochemistry* **60**, 966–990 (2021). 33428850 10.1021/acs.biochem.0c00860 PMC8753971

[459] Taskova, M., Mantsiou, A. & Astakhova, K. Synthetic nucleic acid analogues in gene therapy: an update for peptide-oligonucleotide conjugates. *Chembiochem* **18**, 1671–1682 (2017). 28614621 10.1002/cbic.201700229

[460] Klabenkova, K., Fokina, A. & Stetsenko, D. Chemistry of peptide-oligonucleotide conjugates: a review. *Molecules* **26**, 5420 (2021). 34500849 10.3390/molecules26175420 PMC8434111

[461] Cerrato, C. P., Lehto, T. & Langel, U. Peptide-based vectors: recent developments. *Biomol. Concepts* **5**, 479–488 (2014). 25429600 10.1515/bmc-2014-0024

[462] Tomassi, S. et al. Cationic nucleopeptides as novel non-covalent carriers for the delivery of peptide nucleic acid (PNA) and RNA oligomers. *Bioorg. Med. Chem.* **26**, 2539–2550 (2018). 29656988 10.1016/j.bmc.2018.04.017

[463] Hansen, A. M., Shaikh, A. Y. & Franzyk, H. Facile preparation of pna-peptide conjugates with a polar maleimide-thioether linkage. *Methods Mol. Biol.* **2105**, 97–118 (2020). 32088866 10.1007/978-1-0716-0243-0_6

[464] Schissel, C. K. et al. Cell-penetrating d-peptides retain antisense morpholino oligomer delivery activity. *ACS Bio Med Chem. Au* **2**, 150–160 (2022). 37101743 10.1021/acsbiomedchemau.1c00053 PMC10114648

[465] Hakata, Y. et al. Intracellular delivery of a peptide nucleic acid-based hybrid of an autophagy inducing peptide with a cell-penetrating peptide. *Org. Biomol. Chem.* **18**, 1978–1986 (2020). 32104826 10.1039/c9ob02559f

[466] Linden, G. et al. Efficient antisense inhibition reveals microRNA-155 to restrain a late-myeloid inflammatory programme in primary human phagocytes. *RNA Biol.* **18**, 604–618 (2021). 33622174 10.1080/15476286.2021.1885209 PMC8078538

[467] Barkowsky, G. et al. Antimicrobial activity of peptide-coupled antisense peptide nucleic acids in streptococcus pneumoniae. *Microbiol Spectr.* **10**, e0049722 (2022). 36321914 10.1128/spectrum.00497-22 PMC9784828

[468] Soudah, T., Mogilevsky, M., Karni, R. & Yavin, E. CLIP6-PNA-peptide conjugates: non-endosomal delivery of splice switching oligonucleotides. *Bioconjugate Chem.* **28**, 3036–3042 (2017). 10.1021/acs.bioconjchem.7b00638 29211451

[469] Sheng, L. et al. Comparison of the efficacy of MOE and PMO modifications of systemic antisense oligonucleotides in a severe SMA mouse model. *Nucleic Acids Res* **48**, 2853–2865 (2020). 32103257 10.1093/nar/gkaa126 PMC7102994

[470] Gushchina, L. V. et al. Systemic PPMO-mediated dystrophin expression in the Dup2 mouse model of duchenne muscular dystrophy. *Mol. Ther. Nucl. Acids* **30**, 479–492 (2022). 10.1016/j.omtn.2022.10.025 PMC9678653 36420217

[471] Aslesh, T. et al. DG9-conjugated morpholino rescues phenotype in SMA mice by reaching the CNS via a subcutaneous administration. *JCI Insight* **8**, e160516 (2023). 36719755 10.1172/jci.insight.160516 PMC10077475

[472] Gait, M. J. et al. Cell-penetrating peptide conjugates of steric blocking oligonucleotides as therapeutics for neuromuscular diseases from a historical perspective to current prospects of treatment. *Nucleic Acid Ther.* **29**, 1–12 (2019). 30307373 10.1089/nat.2018.0747 PMC6386087

[473] Hammond, S. M. et al. Systemic peptide-mediated oligonucleotide therapy improves long-term survival in spinal muscular atrophy. *Proc. Natl Acad. Sci. USA* **113**, 10962–10967 (2016). 27621445 10.1073/pnas.1605731113 PMC5047168

[474] Blain, A. M. et al. Peptide-conjugated phosphodiamidate oligomer-mediated exon skipping has benefits for cardiac function in mdx and cmah-/-mdx mouse models of duchenne muscular dystrophy. *PLoS One* **13**, e0198897 (2018). 29912990 10.1371/journal.pone.0198897 PMC6005479

[475] Klein, A. F. et al. Peptide-conjugated oligonucleotides evoke long-lasting myotonic dystrophy correction in patient-derived cells and mice. *J. Clin. Investig.* **129**, 4739–4744 (2019). 31479430 10.1172/JCI128205 PMC6819114

[476] Chioccioli, M. et al. A lung targeted miR-29 mimic as a therapy for pulmonary fibrosis. *EBioMedicine* **85**, 104304 (2022). 36265417 10.1016/j.ebiom.2022.104304 PMC9587275

[477] Jana, A., Narula, P., Chugh, A. & Kulshreshtha, R. Efficient delivery of anti-miR-210 using Tachyplesin, a cell penetrating peptide, for glioblastoma treatment. *Int. J. Pharm.* **572**, 118789 (2019). 31726199 10.1016/j.ijpharm.2019.118789

[478] Schachner-Nedherer, A. L. et al. Biological activity of miRNA-27a using peptide-based drug delivery systems. *Int. J. Nanomed.* **14**, 7795–7808 (2019). 10.2147/IJN.S208446 PMC6768125 31576124

[479] Xu, W. et al. The mirrored cationic peptide as miRNA vehicle for efficient lung cancer therapy. *MedComm* **4**, e273 (2023). 37521428 10.1002/mco2.273 PMC10382604

[480] Wang, J. et al. Strategies for improving the safety and RNAi efficacy of noncovalent peptide/siRNA nanocomplexes. *Adv. Colloid Interface Sci.* **302**, 102638 (2022). 35299136 10.1016/j.cis.2022.102638

[481] Ryu, Y. C., Lee, Y. E. & Hwang, B. H. Efficient and safe small RNA delivery to macrophage using peptide-based nanocomplex. *Biotechnol. Bioeng.* **119**, 482–492 (2022). 34761810 10.1002/bit.27988

[482] Gulley, J. L. et al. Dual inhibition of TGF-beta and PD-L1: a novel approach to cancer treatment. *Mol. Oncol.* **16**, 2117–2134 (2022). 34854206 10.1002/1878-0261.13146 PMC9168966

[483] Wu, L. P. et al. Crossing the blood-brain-barrier with nanoligand drug carriers self-assembled from a phage display peptide. *Nat. Commun.* **10**, 4635 (2019). 31604928 10.1038/s41467-019-12554-2 PMC6789111

[484] Yan, H. et al. Peptide-siRNA nanoparticles targeting NF-kappaB p50 mitigate experimental abdominal aortic aneurysm progression and rupture. *Biomater. Adv.* **139**, 213009 (2022). 35891603 10.1016/j.bioadv.2022.213009 PMC9378586

[485] Yan, H. et al. Induction of WNT16 via peptide-mRNA nanoparticle-based delivery maintains cartilage homeostasis. *Pharmaceutics* **12**, 73 (2020). 31963412 10.3390/pharmaceutics12010073 PMC7022671

[486] Jin, Y. et al. Histone demethylase JMJD3 downregulation protects against aberrant force-induced osteoarthritis through epigenetic control of NR4A1. *Int. J. Oral. Sci.* **14**, 34 (2022). 35831280 10.1038/s41368-022-00190-4 PMC9279410

[487] Zhou, H. F. et al. Peptide-siRNA nanocomplexes targeting NF-kappaB subunit p65 suppress nascent experimental arthritis. *J. Clin. Investig.* **124**, 4363–4374 (2014). 25157820 10.1172/JCI75673 PMC4191028

[488] Ceccanti, M. & Inghilleri, M. RNA interference and neuromuscular diseases: a focus on hereditary transthyretin amyloidosis. *Curr. Gene Ther.* **24**, 6–7 (2024). 37710997 10.2174/1566523223666230913110011

[489] Adams, D., Algalarrondo, V. & Echaniz-Laguna, A. Hereditary transthyretin amyloidosis in the era of RNA interference, antisense oligonucleotide, and CRISPR-Cas9 treatments. *Blood* **142**, 1600–1612 (2023). 37624911 10.1182/blood.2023019884

[490] Keam, S. J. Inotersen: first global approval. *Drugs* **78**, 1371–1376 (2018). 30120737 10.1007/s40265-018-0968-5

[491] Benson, M. D. et al. Inotersen treatment for patients with hereditary transthyretin amyloidosis. *N. Engl. J. Med.* **379**, 22–31 (2018). 29972757 10.1056/NEJMoa1716793 PMC12611561

[492] Coelho, T. et al. Eplontersen for hereditary transthyretin amyloidosis with polyneuropathy. *JAMA* **330**, 1448–1458 (2023). 37768671 10.1001/jama.2023.18688 PMC10540057

[493] Keam, S. J. Vutrisiran: first approval. *Drugs* **82**, 1419–1425 (2022). 35997942 10.1007/s40265-022-01765-5

[494] Gillmore, J. D. et al. CRISPR-Cas9 in vivo gene editing for transthyretin amyloidosis. *N. Engl. J. Med.* **385**, 493–502 (2021). 34215024 10.1056/NEJMoa2107454

[495] Happi Mbakam, C. & Tremblay, J. P. Gene therapy for duchenne muscular dystrophy: an update on the latest clinical developments. *Expert Rev. Neurother.* **23**, 905–920 (2023). 37602688 10.1080/14737175.2023.2249607

[496] Wagner, K. R. et al. Safety, tolerability, and pharmacokinetics of casimersen in patients with duchenne muscular dystrophy amenable to exon 45 skipping: a randomized, double-blind, placebo-controlled, dose-titration trial. *Muscle Nerve* **64**, 285–292 (2021). 34105177 10.1002/mus.27347 PMC9290993

[497] Syed, Y. Y. Eteplirsen: first global approval. *Drugs* **76**, 1699–1704 (2016). 27807823 10.1007/s40265-016-0657-1

[498] Cirak, S. et al. Exon skipping and dystrophin restoration in patients with duchenne muscular dystrophy after systemic phosphorodiamidate morpholino oligomer treatment: an open-label, phase 2, dose-escalation study. *Lancet* **378**, 595–605 (2011). 21784508 10.1016/S0140-6736(11)60756-3 PMC3156980

[499] Wilton-Clark, H. & Yokota, T. Recent trends in antisense therapies for duchenne muscular dystrophy. *Pharmaceutics* **15**, 778 (2023). 36986639 10.3390/pharmaceutics15030778 PMC10054484

[500] Sheikh, O. & Yokota, T. Pharmacology and toxicology of eteplirsen and SRP-5051 for DMD exon 51 skipping: an update. *Arch. Toxicol.* **96**, 1–9 (2022). 34797383 10.1007/s00204-021-03184-z

[501] Mellion, M. et al. PGN-EDO51, an enhanced delivery oligonucleotide (EDO) for the treatment of duchenne muscular dystrophy (DMD): results of a phase 1 study in healthy volunteers (P3-8.004). *Neurology* **100**, 4396 (2023).

[502] Van Daele, S. H., Masrori, P., Van Damme, P. & Van Den Bosch, L. The sense of antisense therapies in ALS. *Trends Mol. Med.* **30**, 252–262 (2024). 38216448 10.1016/j.molmed.2023.12.003

[503] Suzuki, N., Nishiyama, A., Warita, H. & Aoki, M. Genetics of amyotrophic lateral sclerosis: seeking therapeutic targets in the era of gene therapy. *J. Hum. Genet.* **68**, 131–152 (2023). 35691950 10.1038/s10038-022-01055-8 PMC9968660

[504] Fang, T. et al. Gene therapy in amyotrophic lateral sclerosis. *Cells* **11**, 2066 (2022). 35805149 10.3390/cells11132066 PMC9265980

[505] Miller, T. M. et al. Trial of antisense oligonucleotide tofersen for SOD1 ALS. *N. Engl. J. Med.* **387**, 1099–1110 (2022). 36129998 10.1056/NEJMoa2204705

[506] Mccartan, R., Khorkova, O., Volmar, C. H. & Wahlestedt, C. Nucleic acid-based therapeutics for the treatment of central nervous system disorders. *Front. Genet.* **14**, 1250276 (2023). 37662844 10.3389/fgene.2023.1250276 PMC10468602

[507] Nieto-Romero, V. et al. Restored glyoxylate metabolism after AGXT gene correction and direct reprogramming of primary hyperoxaluria type 1 fibroblasts. *iScience* **27**, 109530 (2024). 38577102 10.1016/j.isci.2024.109530 PMC10993186

[508] Groothoff, J. W. et al. Clinical practice recommendations for primary hyperoxaluria: an expert consensus statement from ERKNet and OxalEurope. *Nat. Rev. Nephrol.* **19**, 194–211 (2023). 36604599 10.1038/s41581-022-00661-1

[509] Fargue, S. & Acquaviva Bourdain, C. Primary hyperoxaluria type 1: pathophysiology and genetics. *Clin. Kidney J.* **15**, i4–i8 (2022). 35592619 10.1093/ckj/sfab217 PMC9113437

[510] Scott, L. J. & Keam, S. J. Lumasiran: first approval. *Drugs* **81**, 277–282 (2021). 33405070 10.1007/s40265-020-01463-0

[511] Lakhina, Y., Boulis, N. M. & Donsante, A. Current and emerging targeted therapies for spinal muscular atrophy. *Expert Rev. Neurother.* **23**, 1189–1199 (2023). 37843301 10.1080/14737175.2023.2268276

[512] Ottesen, E. W. et al. Diverse targets of SMN2-directed splicing-modulating small molecule therapeutics for spinal muscular atrophy. *Nucleic Acids Res* **51**, 5948–5980 (2023). 37026480 10.1093/nar/gkad259 PMC10325915

[513] Gowda, V. L., Fernandez-Garcia, M. A., Jungbluth, H. & Wraige, E. New treatments in spinal muscular atrophy. *Arch. Dis. Child.* **108**, 511–517 (2023). 36316089 10.1136/archdischild-2021-323605

[514] Nishio, H. et al. Spinal muscular atrophy: the past, present, and future of diagnosis and treatment. *Int. J. Mol. Sci.* **24**, 11939 (2023). 37569314 10.3390/ijms241511939 PMC10418635

[515] Singh, N. N., Howell, M. D., Androphy, E. J. & Singh, R. N. How the discovery of ISS-N1 led to the first medical therapy for spinal muscular atrophy. *Gene Ther.* **24**, 520–526 (2017). 28485722 10.1038/gt.2017.34 PMC5623086

[516] De Vivo, D. C. et al. Nusinersen initiated in infants during the presymptomatic stage of spinal muscular atrophy: interim efficacy and safety results from the phase 2 nurture study. *Neuromuscul. Disord.* **29**, 842–856 (2019). 31704158 10.1016/j.nmd.2019.09.007 PMC7127286

[517] Montes, J. et al. Nusinersen improves walking distance and reduces fatigue in later-onset spinal muscular atrophy. *Muscle Nerve* **60**, 409–414 (2019). 31298747 10.1002/mus.26633 PMC6771553

[518] Finkel, R. S. et al. Treatment of infantile-onset spinal muscular atrophy with nusinersen: final report of a phase 2, open-label, multicentre, dose-escalation study. *Lancet Child Adolesc. Health* **5**, 491–500 (2021). 34089650 10.1016/S2352-4642(21)00100-0

[519] Neuzillet, C. et al. Targeting the TGFβ pathway for cancer therapy. *Pharmacol. Ther.* **147**, 22–31 (2015). 25444759 10.1016/j.pharmthera.2014.11.001

[520] Andrews, D. W. et al. Phase Ib clinical trial of IGV-001 for patients with newly diagnosed glioblastoma. *Clin. Cancer Res.* **27**, 1912–1922 (2021). 33500356 10.1158/1078-0432.CCR-20-3805

[521] Lee, I. Y. et al. Autologous cell immunotherapy (IGV-001) with IGF-1R antisense oligonucleotide in newly diagnosed glioblastoma patients. *Future Oncol.* **20**, 579–591 (2024). 38060340 10.2217/fon-2023-0702

[522] Weber, J. S. et al. Individualised neoantigen therapy mRNA-4157 (V940) plus pembrolizumab versus pembrolizumab monotherapy in resected melanoma (KEYNOTE-942): a randomised, phase 2b study. *Lancet* **403**, 632–644 (2024). 38246194 10.1016/S0140-6736(23)02268-7

[523] Yao, R., Xie, C. & Xia, X. Recent progress in mRNA cancer vaccines. *Hum. Vaccines Immunother.* **20**, 2307187 (2024). 10.1080/21645515.2024.2307187 PMC10826636 38282471

[524] Nashine, S. Potential therapeutic candidates for age-related macular degeneration (AMD). *Cells* **10**, 2483 (2021). 34572131 10.3390/cells10092483 PMC8464988

[525] Nakamura, Y. Multiple therapeutic applications of RBM-007, an anti-FGF2 aptamer. *Cells* **10**, 1617 (2021). 34203430 10.3390/cells10071617 PMC8305614

[526] Jimenez, A. et al. SYL1801: preclinical efficacy and safety of a sirna-based eye drops treatment for age related macular degeneration. *Investig. Ophthalmol. Vis. Sci.* **60**, 5389 (2019).

[527] Jimenez, A. I., Ruz, V., Vargas, B. & Bleau, A. M. Phase I of SYL1801, a new siRNA delivered in eye drops for age-related macular degeneration. *Investig. Ophthalmol. Vis. Sci.* **63**, 337–F0168 (2022).

[528] Moreno-Montanes, J., Bleau, A. M. & Jimenez, A. I. Tivanisiran, a novel siRNA for the treatment of dry eye disease. *Expert Opin. Investig. Drugs* **27**, 421–426 (2018). 29569947 10.1080/13543784.2018.1457647

[529] Valdes-Arias, D. et al. Recent United States developments in the pharmacological treatment of dry eye disease. *Drugs* **84**, 549–563 (2024). 38652355 10.1007/s40265-024-02031-6 PMC11189955

[530] Kuo, Y. K. et al. Dry eye disease: a review of epidemiology in taiwan, and its clinical treatment and merits. *J. Clin. Med.* **8**, 1227 (2019). 31443274 10.3390/jcm8081227 PMC6722537

[531] Dulla, K. et al. Splice-modulating oligonucleotide QR-110 restores CEP290 mRNA and function in human c.2991+1655A>G LCA10 models. *Mol. Ther. Nucl. Acids* **12**, 730–740 (2018). 10.1016/j.omtn.2018.07.010 PMC6092551 30114557

[532] Cideciyan, A. V. et al. Durable vision improvement after a single intravitreal treatment with antisense oligonucleotide in CEP290-LCA: replication in two eyes. *Am. J. Ophthalmol. Case Rep.* **32**, 101873 (2023). 37388818 10.1016/j.ajoc.2023.101873 PMC10302566

[533] Dulla, K. et al. Antisense oligonucleotide-based treatment of retinitis pigmentosa caused by USH2A exon 13 mutations. *Mol. Ther.* **29**, 2441–2455 (2021). 33895329 10.1016/j.ymthe.2021.04.024 PMC8353187

[534] Shi, Y. et al. Epigenetic regulation in cardiovascular disease: mechanisms and advances in clinical trials. *Signal Transduct. Target Ther.* **7**, 200 (2022). 35752619 10.1038/s41392-022-01055-2 PMC9233709

[535] Mhaimeed, O. et al. The importance of LDL-C lowering in atherosclerotic cardiovascular disease prevention: lower for longer is better. *Am. J. Prev. Cardiol.* **18**, 100649 (2024). 38576462 10.1016/j.ajpc.2024.100649 PMC10992711

[536] Sawhney, J. P. et al. CSI clinical practice guidelines for dyslipidemia management: Executive summary. *Indian Heart J.* **76**, S6–S19 (2024). 38052658 10.1016/j.ihj.2023.11.271 PMC11019331

[537] Hummelgaard, S. et al. Targeting PCSK9 to tackle cardiovascular disease. *Pharmacol. Ther.* **249**, 108480 (2023). 37331523 10.1016/j.pharmthera.2023.108480

[538] Ferri, N. et al. Proprotein convertase subtilisin kexin type 9 (PCSK9) secreted by cultured smooth muscle cells reduces macrophages LDLR levels. *Atherosclerosis* **220**, 381–386 (2012). 22176652 10.1016/j.atherosclerosis.2011.11.026

[539] Lamb, Y. N. Inclisiran: first approval. *Drugs* **81**, 389–395 (2021). 33620677 10.1007/s40265-021-01473-6 PMC7900795

[540] Ray, K. K. et al. Two phase 3 trials of inclisiran in patients with elevated LDL cholesterol. *N. Engl. J. Med.* **382**, 1507–1519 (2020). 32187462 10.1056/NEJMoa1912387

[541] Ray, K. K. et al. Inclisiran and cardiovascular events: a patient-level analysis of phase III trials. *Eur. Heart J.* **44**, 129–138 (2023). 36331326 10.1093/eurheartj/ehac594 PMC9825807

[542] Tsimikas, S. et al. Lipoprotein(a) reduction in persons with cardiovascular disease. *N. Engl. J. Med.* **382**, 244–255 (2020). 31893580 10.1056/NEJMoa1905239

[543] Vinci, P. et al. Lipoprotein(a) as a risk factor for cardiovascular diseases: pathophysiology and treatment perspectives. *Int. J. Environ. Res. Public Health* **20**, 6721 (2023). 37754581 10.3390/ijerph20186721 PMC10531345

[544] Wang, S. et al. The relationship between lipoprotein(a) and risk of cardiovascular disease: a mendelian randomization analysis. *Eur. J. Med. Res.* **27**, 211 (2022). 36303257 10.1186/s40001-022-00825-6 PMC9608881

[545] Malick, W. A., Goonewardena, S. N., Koenig, W. & Rosenson, R. S. Clinical trial design for lipoprotein(a)-lowering therapies: JACC focus seminar 2/3. *J. Am. Coll. Cardiol.* **81**, 1633–1645 (2023). 37076218 10.1016/j.jacc.2023.02.033

[546] Nissen, S. E. et al. Lepodisiran, an extended-duration short interfering RNA targeting lipoprotein(a): a randomized dose-ascending clinical trial. *JAMA* **330**, 2075–2083 (2023). 37952254 10.1001/jama.2023.21835 PMC10641766

[547] O’donoghue, M. L. et al. Small interfering RNA to reduce lipoprotein(a) in cardiovascular disease. *N. Engl. J. Med.* **387**, 1855–1864 (2022). 36342163 10.1056/NEJMoa2211023

[548] Koren, M. J. et al. Preclinical development and phase 1 trial of a novel siRNA targeting lipoprotein(a). *Nat. Med.* **28**, 96–103 (2022). 35027752 10.1038/s41591-021-01634-w

[549] Khan, R. S. & Frishman, W. H. Zilebesiran: a promising antihypertensive therapy inhibiting angiotensinogen synthesis. *Cardiol. Rev*. 10.1097/CRD.0000000000000645 (2024). 10.1097/CRD.0000000000000645 38385680

[550] Desai, A. S. et al. Zilebesiran, an RNA interference therapeutic agent for hypertension. *N. Engl. J. Med.* **389**, 228–238 (2023). 37467498 10.1056/NEJMoa2208391

[551] Bakris, G. L. et al. RNA interference with zilebesiran for mild to moderate hypertension: the KARDIA-1 randomized clinical trial. *JAMA* **331**, 740–749 (2024). 38363577 10.1001/jama.2024.0728 PMC10873804

[552] Szabo, G. T., Mahiny, A. J. & Vlatkovic, I. COVID-19 mRNA vaccines: platforms and current developments. *Mol. Ther.* **30**, 1850–1868 (2022). 35189345 10.1016/j.ymthe.2022.02.016 PMC8856755

[553] Fang, E. et al. Advances in COVID-19 mRNA vaccine development. *Signal Transduct. Target Ther.* **7**, 94 (2022). 35322018 10.1038/s41392-022-00950-y PMC8940982

[554] Baden, L. R. et al. Efficacy and safety of the mRNA-1273 SARS-CoV-2 vaccine. *N. Engl. J. Med.* **384**, 403–416 (2021). 33378609 10.1056/NEJMoa2035389 PMC7787219

[555] Polack, F. P. et al. Safety and efficacy of the BNT162b2 mRNA COVID-19 vaccine. *N. Engl. J. Med.* **383**, 2603–2615 (2020). 33301246 10.1056/NEJMoa2034577 PMC7745181

[556] Grudda, T. et al. Integrated hepatitis B virus DNA maintains surface antigen production during antiviral treatment. *J. Clin. Investig.* **132**, e161818 (2022). 35797115 10.1172/JCI161818 PMC9473722

[557] Mak, L. Y. et al. Bepirovirsen (GSK3228836) in chronic hepatitis B infection: an evaluation of phase II progress. *Expert Opin. Investig. Drugs* **32**, 971–983 (2023). 37902953 10.1080/13543784.2023.2277389

[558] Yuen, M.-F. et al. Efficacy and safety of bepirovirsen in chronic hepatitis b infection. *N. Engl. J. Med.* **387**, 1957–1968 (2022). 36346079 10.1056/NEJMoa2210027

[559] Yuen, M. F. et al. Safety, tolerability and antiviral activity of the antisense oligonucleotide bepirovirsen in patients with chronic hepatitis B: a phase 2 randomized controlled trial. *Nat. Med.* **27**, 1725–1734 (2021). 34642494 10.1038/s41591-021-01513-4 PMC8516644

[560] Yuen, M. F. et al. Efficacy and safety of the siRNA JNJ-73763989 and the capsid assembly modulator JNJ-56136379 (bersacapavir) with nucleos(t)ide analogues for the treatment of chronic hepatitis B virus infection (REEF-1): a multicentre, double-blind, active-controlled, randomised, phase 2b trial. *Lancet Gastroenterol. Hepatol.* **8**, 790–802 (2023). 37442152 10.1016/S2468-1253(23)00148-6

[561] Gupta, S. V. et al. Clinical and preclinical single-dose pharmacokinetics of VIR-2218, an RNAi therapeutic targeting HBV infection. *Drugs RD* **21**, 455–465 (2021). 10.1007/s40268-021-00369-w PMC8602582 34741731

[562] Soriano, V. Hepatitis B gene therapy coming to age. *AIDS Rev.* **20**, 125–127 (2018). 29938706

[563] Jain, S., Kaur, J., Prasad, S. & Roy, I. Nucleic acid therapeutics: a focus on the development of aptamers. *Expert Opin. Drug Discov.* **16**, 255–274 (2021). 32990095 10.1080/17460441.2021.1829587

[564] Sabir, F. et al. DNA based and stimuli-responsive smart nanocarrier for diagnosis and treatment of cancer: applications and challenges. *Cancers (Basel)* **13**, 3396 (2021). 34298610 10.3390/cancers13143396 PMC8307033

[565] Augustine, R. et al. pH-responsive polypeptide-based smart nano-carriers for theranostic applications. *Molecules* **24**, 2961 (2019). 31443287 10.3390/molecules24162961 PMC6719039

[566] Dirin, M. & Winkler, J. Influence of diverse chemical modifications on the ADME characteristics and toxicology of antisense oligonucleotides. *Expert Opin. Biol. Ther.* **13**, 875–888 (2013). 23451977 10.1517/14712598.2013.774366

[567] Amantana, A. et al. Pharmacokinetics, biodistribution, stability and toxicity of a cell-penetrating peptide-morpholino oligomer conjugate. *Bioconjugate Chem.* **18**, 1325–1331 (2007). 10.1021/bc070060v 17583927

[568] Gao, X. et al. The association of autophagy with polyethylenimine-induced cytotoxicity in nephritic and hepatic cell lines. *Biomaterials* **32**, 8613–8625 (2011). 21903261 10.1016/j.biomaterials.2011.07.047

[569] Takakusa, H. et al. Drug Metabolism and pharmacokinetics of antisense oligonucleotide therapeutics: typical profiles, evaluation approaches, and points to consider compared with small molecule drugs. *Nucleic Acid Ther.* **33**, 83–94 (2023). 36735616 10.1089/nat.2022.0054 PMC10066781

[570] Hashida, M. Role of pharmacokinetic consideration for the development of drug delivery systems: A historical overview. *Adv. Drug Deliv. Rev.* **157**, 71–82 (2020). 32565225 10.1016/j.addr.2020.06.015

[571] Jiang, R. et al. Factors influencing ADME properties of therapeutic antisense oligonucleotides: physicochemical characteristics and beyond. *Curr. Drug Metab.* **24**, 536–552 (2023). 37076460 10.2174/1389200224666230418092626

[572] Bosgra, S. et al. The pharmacokinetics of 2’-O-methyl phosphorothioate antisense oligonucleotides: experiences from developing exon skipping therapies for duchenne muscular dystrophy. *Nucleic Acid Ther.* **29**, 305–322 (2019). 31429628 10.1089/nat.2019.0805

[573] Gonzalez-Barriga, A. et al. Intracellular distribution and nuclear activity of antisense oligonucleotides after unassisted uptake in myoblasts and differentiated myotubes in vitro. *Nucleic Acid Ther.* **27**, 144–158 (2017). 28375678 10.1089/nat.2016.0641 PMC5467152

[574] Wang, L. & Ji, C. Advances in quantitative bioanalysis of oligonucleotide biomarkers and therapeutics. *Bioanalysis* **8**, 143–155 (2016). 26652713 10.4155/bio.15.234

[575] Xiao, X. et al. Multi-functional peptide-microRNA nanocomplex for targeted microRNA delivery and function imaging. *Chemistry* **24**, 2277–2285 (2018). 29226432 10.1002/chem.201705695

[576] Migliorati, J. M. et al. Absorption, distribution, metabolism, and excretion of US food and drug administration-approved antisense oligonucleotide drugs. *Drug Metab. Dispos.* **50**, 888–897 (2022). 35221287 10.1124/dmd.121.000417 PMC11022858

[577] Miao, Y. et al. Current status and trends in small nucleic acid drug development: leading the future. *Acta Pharm. Sin. B* **14**, 3802–3817 (2024). 39309508 10.1016/j.apsb.2024.05.008 PMC11413693

[578] Perry, C. M. & Balfour, J. A. Fomivirsen. *Drugs* **57**, 375–381 (1999). 10193689 10.2165/00003495-199957030-00010

[579] Stein, E. A. et al. Apolipoprotein B synthesis inhibition with mipomersen in heterozygous familial hypercholesterolemia: results of a randomized, double-blind, placebo-controlled trial to assess efficacy and safety as add-on therapy in patients with coronary artery disease. *Circulation* **126**, 2283–2292 (2012). 23060426 10.1161/CIRCULATIONAHA.112.104125

[580] Hoy, S. M. Nusinersen: first global approval. *Drugs* **77**, 473–479 (2017). 28229309 10.1007/s40265-017-0711-7

[581] Finkel, R. S. et al. Nusinersen versus sham control in infantile-onset spinal muscular atrophy. *N. Engl. J. Med.* **377**, 1723–1732 (2017). 29091570 10.1056/NEJMoa1702752

[582] Witztum, J. L. et al. Volanesorsen and triglyceride levels in familial chylomicronemia syndrome. *N. Engl. J. Med.* **381**, 531–542 (2019). 31390500 10.1056/NEJMoa1715944

[583] Clemens, P. R. et al. Safety, tolerability, and efficacy of viltolarsen in boys with duchenne muscular dystrophy amenable to exon 53 skipping: a phase 2 randomized clinical trial. *JAMA Neurol.* **77**, 982–991 (2020). 32453377 10.1001/jamaneurol.2020.1264 PMC7251505

[584] Syed, Y. Y. Givosiran: a review in acute hepatic porphyria. *Drugs* **81**, 841–848 (2021). 33871817 10.1007/s40265-021-01511-3

[585] Kang, C. Avacincaptad pegol: first approval. *Drugs* **83**, 1447–1453 (2023). 37814173 10.1007/s40265-023-01948-8

[586] Grana, C. et al. Efficacy and safety of COVID-19 vaccines. *Cochrane Database Syst. Rev.* **12**, CD015477 (2022). 36473651 10.1002/14651858.CD015477 PMC9726273

[587] Britton, A. et al. Use of respiratory syncytial virus vaccines in adults aged ≥60 years: updated recommendations of the advisory committee on immunization practices - United States. *Morb. Mortal. Wkly. Rep.* **73**, 696–702 (2024). 10.15585/mmwr.mm7332e1 39146277

[588] Maurer, M. S. Overview of current and emerging therapies for amyloid transthyretin cardiomyopathy. *Am. J. Cardiol.* **185**, S23–S34 (2022). 36371281 10.1016/j.amjcard.2022.10.014

[589] Helm, J., Schöls, L. & Hauser, S. Towards personalized allele-specific antisense oligonucleotide therapies for toxic gain-of-function neurodegenerative diseases. *Pharmaceutics* **14**, 1708 (2022). 36015334 10.3390/pharmaceutics14081708 PMC9416334

[590] Young, G. et al. Efficacy and safety of fitusiran prophylaxis in people with haemophilia A or haemophilia B with inhibitors (ATLAS-INH): a multicentre, open-label, randomised phase 3 trial. *Lancet* **401**, 1427–1437 (2023). 37003287 10.1016/S0140-6736(23)00284-2

[591] Riedl, M. A. et al. Efficacy and safety of donidalorsen for hereditary angioedema. *N. Engl. J. Med.* **391**, 21–31 (2024). 38819395 10.1056/NEJMoa2402478

[592] Riedl, M. A. et al. Clinical progress in hepatic targeting for novel prophylactic therapies in hereditary angioedema. *J. Allergy Clin. Immunol. Pract.* **12**, 911–918 (2024). 38142864 10.1016/j.jaip.2023.12.025

[593] Badri, P. et al. Pharmacokinetic and pharmacodynamic properties of cemdisiran, an RNAi therapeutic targeting complement component 5, in healthy subjects and patients with paroxysmal nocturnal hemoglobinuria. *Clin. Pharmacokinet.* **60**, 365–378 (2021). 33047216 10.1007/s40262-020-00940-9 PMC9203406

[594] Caravaca-Fontan, F., Gutierrez, E., Sevillano, A. M. & Praga, M. Targeting complement in IgA nephropathy. *Clin. Kidney J.* **16**, ii28–ii39 (2023). 38053977 10.1093/ckj/sfad198 PMC10695513

[595] Woodcock, I. R. et al. A phase 2 open-label study of the safety and efficacy of weekly dosing of ATL1102 in patients with non-ambulatory duchenne muscular dystrophy and pharmacology in mdx mice. *PLoS One* **19**, e0294847 (2024). 38271438 10.1371/journal.pone.0294847 PMC10810432

[596] Wengert, E. R. et al. Targeted augmentation of nuclear gene output (TANGO) of scn1a rescues parvalbumin interneuron excitability and reduces seizures in a mouse model of dravet syndrome. *Brain Res* **1775**, 147743 (2022). 34843701 10.1016/j.brainres.2021.147743

[597] Longhurst, H. J. et al. CRISPR-Cas9 in vivo gene editing of KLKB1 for hereditary angioedema. *N. Engl. J. Med.* **390**, 432–441 (2024). 38294975 10.1056/NEJMoa2309149

[598] Baek, R. et al. Characterizing the mechanism of action for mRNA therapeutics for the treatment of propionic acidemia, methylmalonic acidemia, and phenylketonuria. *Nat. Commun.* **15**, 3804 (2024). 38714648 10.1038/s41467-024-47460-9 PMC11076592

[599] Jeffrey, S. et al. Individualized neoantigen therapy mRNA-4157 (V940) plus pembrolizumab in resected melanoma: 3-year update from the mRNA-4157-P201 (KEYNOTE-942) trial. *J. Clin. Oncol.* **42**, LBA9512 (2024).

[600] Rojas, L. A. et al. Personalized RNA neoantigen vaccines stimulate T cells in pancreatic cancer. *Nature* **618**, 144–150 (2023). 37165196 10.1038/s41586-023-06063-y PMC10171177

[601] Steurer, M. et al. Olaptesed pegol (NOX-A12) with bendamustine and rituximab: a phase IIa study in patients with relapsed/refractory chronic lymphocytic leukemia. *Haematologica* **104**, 2053–2060 (2019). 31097627 10.3324/haematol.2018.205930 PMC6886437

[602] Russell, S. R. et al. Intravitreal antisense oligonucleotide sepofarsen in leber congenital amaurosis type 10: a phase 1b/2 trial. *Nat. Med.* **28**, 1014–1021 (2022). 35379979 10.1038/s41591-022-01755-w PMC9117145

[603] Dreismann, A. K. et al. Gene targeting as a therapeutic avenue in diseases mediated by the complement alternative pathway. *Immunol. Rev.* **313**, 402–419 (2023). 36369963 10.1111/imr.13149 PMC10099504

[604] Tselepis, A. D. Treatment of Lp(a): is it the future or are we ready today? *Curr. Atheroscler. Rep.* **25**, 679–689 (2023). 37668953 10.1007/s11883-023-01141-y PMC10564831

[605] Tardif, J. C. et al. Apolipoprotein C-III reduction in subjects with moderate hypertriglyceridaemia and at high cardiovascular risk. *Eur. Heart J.* **43**, 1401–1412 (2022). 35025993 10.1093/eurheartj/ehab820 PMC8986458

[606] Gouni-Berthold, I., Schwarz, J. & Berthold, H. K. Updates in drug treatment of severe hypertriglyceridemia. *Curr. Atheroscler. Rep.* **25**, 701–709 (2023). 37642858 10.1007/s11883-023-01140-z PMC10564803

[607] Rosenson, R. S. et al. Zodasiran, an RNAi therapeutic targeting ANGPTL3, for mixed hyperlipidemia. *N. Engl. J. Med.* **391**, 913–925 (2024). 38809174 10.1056/NEJMoa2404147

[608] Huang, S. A. et al. Abstract 14387: dose-related reductions in blood pressure with a RNA interference (RNAi) therapeutic targeting angiotensinogen in hypertensive patients: interim results from a first-in-human phase 1 study of ALN-AGT01. *Circulation* **142**, A14387 (2020).

[609] Wilson, E. et al. Efficacy and safety of an mRNA-based RSV PreF vaccine in older adults. *N. Engl. J. Med.* **389**, 2233–2244 (2023). 38091530 10.1056/NEJMoa2307079

[610] Hu, X. et al. Human cytomegalovirus mRNA-1647 vaccine candidate elicits potent and broad neutralization and higher antibody-dependent cellular cytotoxicity responses than the gB/MF59 vaccine. *J. Infect. Dis.* **230**, 455–466 (2024). 38324766 10.1093/infdis/jiad593 PMC11326847

[611] Zanardi, T. A. et al. Safety, pharmacokinetic, and pharmacodynamic evaluation of a 2’-(2-methoxyethyl)-d-ribose antisense oligonucleotide-triantenarry *n*-acetyl-galactosamine conjugate that targets the human transmembrane protease serine 6. *J. Pharmacol. Exp. Ther.* **377**, 51–63 (2021). 33431610 10.1124/jpet.120.000222

[612] Prikhodko, V. A., Bezborodkina, N. N. & Okovityi, S. V. Pharmacotherapy for non-alcoholic fatty liver disease: emerging targets and drug candidates. *Biomedicines* **10**, 274 (2022). 35203484 10.3390/biomedicines10020274 PMC8869100

[613] Bujko, K. et al. Signaling of the complement cleavage product anaphylatoxin C5a through C5aR (CD88) contributes to pharmacological hematopoietic stem cell mobilization. *Stem Cell Rev. Rep.* **13**, 793–800 (2017). 28918528 10.1007/s12015-017-9769-6 PMC5730632

